# SENP3 alleviates osteoporosis via promoting SIRT3 transcription through the increase of DLX2 stability via SUMO2/3

**DOI:** 10.1007/s10565-025-10052-4

**Published:** 2025-06-10

**Authors:** Jie Bu, Xuezheng Xu, Yi Luo, Jianfan Liu, Feng Zhou

**Affiliations:** Department of Orthopedics, The Affiliated Cancer Hospital of Xiangya School of Medicine, Central South University/Hunan Cancer Hospital, Changsha, Hunan Province 410013 P. R. China

**Keywords:** SENP3, SIRT3, DLX2, SUMO2/3, Osteoporosis

## Abstract

**Background:**

Recent studies have indicated a close relationship between SENP3 and osteoporosis. However, the detailed molecular mechanism of SENP3 mediating osteoporosis has not been well studied. The goal of this work was to study the specific mechanism by which SENP3 regulates downstream genes through deSUMOylation and thus affects the progression of osteoporosis.

**Methods:**

Osteogenic differentiation was evaluated through osteogenic marker genes, mineralization, and ALP activity, which were detected by qPCR, western blot, and ALP staining assays. Osteoporosis was assessed in OVX mice assessed using qPCR, Micro-CT, and H&E staining assays. The levels of SENP3, DLX2, and SIRT3 were monitored using qPCR and western blot assays. The SUMOylated modification of DLX2 was evaluated using Co-IP and IP assays. The binding of DLX2 to the SIRT3 promoter was confirmed with ChIP, qPCR, dual-luciferase reporter and western blot assays.

**Results:**

SENP3, DLX2, and SIRT3 expressions were decreased in tissues of OVX mice. Mechanically, SENP3 inhibited SUMOylated modification of DLX2 and augmented DLX2 stability. Addition of SENP3 accelerated osteogenic differentiation via regulating DLX2. Moreover, DLX2 bound to SIRT3 promoter and accelerated SIRT3 transcription. DLX2 depletion-induced impeditive effects on osteogenic differentiation were reversed by SIRT3 overexpression. Moreover, DLX2 addition counteracted sh-SENP3-induced inhibitory effect on osteogenic differentiation, which was partially reversed by SIRT3 knockdown. Furthermore, SENP3 alleviated osteoporosis in OVX mice by regulating DLX2/SIRT3 axis.

**Conclusion:**

Addition of SENP3 accelerated osteogenic differentiation and relieved osteoporosis via increasing SIRT3 transcription by the enhance of DLX2 stability via SUMO2/3.

**Graphical Abstract:**

This graphical abstract illustrated the molecular mechanism by which SENP3 alleviates osteoporosis. SENP3 promoted osteogenic differentiation and alleviated osteoporosis. SENP3 increased SIRT3 transcription by stabilizing DLX2. SENP3 removed SUMO2/3 modifications from DLX2 to enhance its stability. The SENP3–DLX2–SIRT3 axis played a critical role in bone formation.

**Graphical Abstract:**

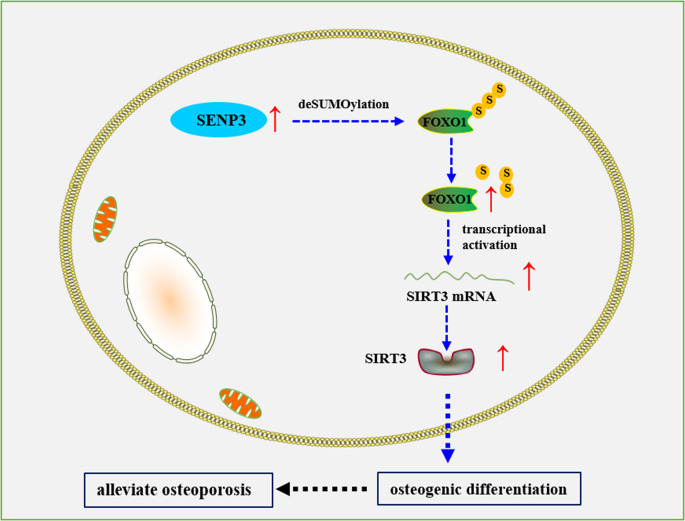

**Supplementary Information:**

The online version contains supplementary material available at 10.1007/s10565-025-10052-4.

## Introduction

Osteoporosis is a common systemic bone disease in clinic (You et al. 2021). It is marked by the destruction of bone microstructure and low bone mass, which gradually results in weak bones and an increased risk of fracture (Guo, Y.c,, et al. 2018). In osteoporosis, the adipogenic differentiation of BMSCs is increased and the osteogenic differentiation is decreased (Shen, et al. 2020; Kim et al. 2018). The imbalance of BMSC differentiation is the major cause of osteoporosis (You et al. 2023). To investigate the mechanism of osteogenic differentiation of BMSCs is a necessary behavior to alleviate osteoporosis.

In almost all eukaryotes, SUMO proteins can embellish proteins via adhering to lysine side chains to produce branched-chain proteins (Yau, et al. 2021). The SUMOylation process is dynamic and modulates many processes that play pivotal functions in progression, such as transcriptional control, signal transduction, and protein aggregation (Yau et al. 2020). SENPs mediate the reverse deSUMOylation process (Chen et al. 2020). SENP3, a member of the SENP family, modulates the occurrence of multiple diseases (Liu et al. 2023). For instance, SENP3 exacerbated apoptosis of renal tubular epithelial cells in LPS-triggered acute kidney injury via Drp1 deSUMOylation (Wang et al. 2022). SENP3 suppressed macrophage foam cell formation via deSUMOylation of NLRP3, which provided a new approach for atherosclerosis treatment (Chen, et al. 2024). Moreover, a former paper unveiled that addition of SENP3 enhances PPAR-γ transcription by upregulate of HIF-1α stability through SUMO2/3 and involves in the development of type II diabetic osteoporosis (Wang et al. 2023). SENP3 activated HOX gene level by catalyzing RbBP5 deSUMOylation to regulate human stem cell osteogenic differentiation (Zhang, et al. 2020). However, the specific mechanism by which SENP3 regulates osteoporosis remains unclear.

DLX2 is one of the genes in the distal-less family, and its role in craniofacial bone development is well known (Depew et al. 2002). Zeng et al. elaborated DLX2 plays its role in osteogenic modulation in a Wnt/β-catenin-dependent manner, revealing as an original target for accelerating bone formation in oral and maxillofacial tissues (Zeng et al. 2020). Zhang et al. unveiled that addition of DLX2 facilitates osteogenic differentiation of mouse BMSCs and MC3 T3-E1 cells, and promotes bone formation through direct increase of ALP and osteocalcin (Zhang et al. 2019). Moreover, DLX2 as a transcription factor activated Wnt1 transcription and regulated Wnt/β-catenin pathway to accelerate osteogenic differentiation of hBMSC (Zeng et al. 2020). Trkb neurotrophic factor receptor expression was transcriptionally regulated by DLX2 during mouse retinal development (Melo et al. 2008). JASPAR predicted that DLX2 had binding sites in the promoter region of SIRT3. Gao et al. uncovered that the alleviating effect of SIRT3 on senile osteoporosis (Guo et al. 2021). However, whether DLX2, as a transcription factor, excites SIRT3 transcription and implicates in the modulation of osteoporosis by binding SIRT3 promoter is unclear.

On this basis, we conjectured that the SENP3/DLX2/SIRT3 axis has a regulatory effect on osteoporosis. This study expands the understanding of the mechanism by which SENP3 regulates osteoporosis and provides a new perspective for its treatment.

## Materials and methods

### Animal experiments

Female C57BL/6 mice (20–25 g, 11–12 weeks; Hunan SJA Laboratory Animal Co., Ltd., Changsha, China) were housed under controlled conditions with a 12-h light/dark cycle at 22–24 °C with 50–60% humidity. After a 7-day adaptation period, mice underwent either dorsal ovariectomy (OVX, n = 18) or sham surgery (sham, n = 6). Then, the ovariectomized mice were randomly divided into 3 groups: OVX (n = 6), OVX + oe-NC (n = 6), OVX + oe-SENP3 (n = 6). AAV of oe-NC and oe-SENP3 were constructed and packaged by Hanbio (Shanghai, China). OVX mice were injected AAV with oe-NC, oe-SENP3, or PBS through tail veins once a week for 4 weeks. The mice in sham operation group were injected with the same amount of PBS via the tail vein once a week for 4 consecutive weeks. After 4 weeks of modeling, mice were euthanized by cervical dislocation. The histopathological structure of the mice femur was analyzed by pathological section. All animal experiments were approved by the Affiliated Cancer Hospital of Xiangya School of Medicine, Central South University (NO. SBQLL-2022–253).

## microCT analysis

Bone trabecular scanning was performed using a micro-CT imaging instrument (Quantum GX, PerkinElemer) with the following scanning parameters: 80 kV voltage, 500 μA current, medium resolution, and an isotropic voxel size of 40.5 μm. After scanning, post-analysis were performed using the Imalytics preclinical software (Gremse-IT, Germany).

Regions of interest (ROIs) were defined in the distal femoral metaphysis, starting 0.5 mm below the growth plate and extending 1 mm in the distal direction. Bone segmentation was performed using a global thresholding algorithm. 3D structural parameters of trabecular bone were analyzed, including BV/TV, Tb.N, Tb.Th, and BMD, based on standard guidelines for microstructural bone analysis.

## H&E staining

The femurs were fixed in 4% paraformaldehyde for 24 h, followed by decalcification in EDTA solution over a 21-day period. After decalcification, the samples were paraffin-embedded, and sagittal sections of 3-μm thickness were prepared. H&E staining was then carried out using standard procedures.

## Cell culture and osteogenic induction

Human BMSCs were obtained from ATCC (PCS-500–012, ATCC, Manassas, VA, USA). All cells were preserved in BMSC whole medium at 37 °C and 5% CO_2_. 4–6 generations of BMSCs were utilized for follow-up assays. For osteogenic differentiation, BMSCs were cultured with osteogenic differentiation medium (DMEM containing 100 U/mL penicillin, 10% FBS, 100 μg/mL streptomycin, 50 μM ascorbic acid, and 10 mM β-glycerol phosphate) for 7 days. Osteogenic differentiation medium was replaced every three days. ALP activity analysis and ARS staining were utilized to analyze osteogenic differentiation.

## ALP analysis

For ALP analysis, BMSCs were seeded in 24-well plates at a density of 2 × 10^4^ cells/well. After the cells reached ~ 70–80% confluence, osteogenic induction was performed using osteogenic differentiation medium (consisting of 10 mM β-glycerophosphate, 50 µg/mL ascorbic acid, and 100 nM dexamethasone in DMEM supplemented with 10% FBS). Medium replacement occurred every 2–3 days. After 7 days of induction, cells stained with BCIP/NBT solution (Beyotime) to detect ALP activity. After staining, the dye solution was discarded, and the cells were rinsed three times with PBS. Images were acquired using a light microscope.

Additionally, a quantitative ALP activity assay was performed using an ALP activity kit (Beyotime). Briefly, the cells were lysed, and the supernatant was collected. The reaction mixture was incubated with the lysate at 37 °C for 15 min, followed by addition of the stop solution. Absorbance was read at 405 nm using a microplate reader.

## ARS staining

For ARS staining, BMSCs were seeded in 24-well plates at a density of 2 × 10^4^ cells/well. After reaching appropriate confluence, osteogenic differentiation was induced as described above. After 7 days of induction, cells were washed with PBS, fixed in 4% paraformaldehyde for 30 min, and stained with 1% Alizarin Red S (ARS, Solarbio) solution (pH 4.2) for 20 min. After thorough washing with distilled water to remove excess dye, mineralized nodules were observed and photographed under a light microscope (Zeng et al. 2023).

## Cell transfection

ShRNAs specific for SENP3 (sh-SENP3), DLX2 (sh-DLX2), and SIRT3 (sh-SIRT3) and associative negative control (sh-NC) were obtained from Genechem (Shanghai, China). For overexpression of SENP3 and DLX2, the overexpression vector (oe-SENP3 and oe-DLX2) and control vector (oe-NC) were also purchased from Genechem. These plasmids transfected into cell using Lipofectamine 2000.

## Western blot

Total proteins were extracted by RIPA buffer and quantified by a BCA kit (Beyotime). Equal amounts of proteins were separated by 10% SDS-PAGE along with a pre-stained protein marker (WJ102, Epizyme Biotech), and then transferred to PVDF membranes (Millipore, U.S.A.). 5% skimmed milk was used to block membranes. The protein bands were immunoblotted with specific primary antibodies: anti-SENP3 (#5591, Cell Signaling Technology, 1:1000), anti-DLX2 (ab272902, Abcam, 1:5000), anti-SUMO2/3 (#4971, Cell Signaling Technology, 1:1000), anti-OPN (ab283656, Abcam, 1:1000), anti-OCN (#ER61846, HUABIO, 1:500), anti-RUNX2 (ab76956, Abcam, 1:1000), anti-SIRT3 (ab189860, Abcam, 1:500), and anti-GAPDH (ab8245, Abcam, 1/500). Then, the membranes were incubated with secondary antibodies. Protein bands were displayed using an enhanced chemiluminescence kit (Applygen, Beijing, China).

## qRT-PCR

Total RNA was extracted with TRIzol reagent (Invitrogen), reverse-transcribed using PrimeScript RT Master Mix (TaKaRa, Dalian, China), and analyzed by qRT-PCR with SYBR Green PCR Master Mix Plus (Takara) on an FTC-3000 System. Each gene was standardized to GAPDH. Relative gene levels were analyzed using the 2^−ΔΔCt^ method. The primers of the gene shown is as follows: SENP3 (F) 5′-ATCCACCTGGAGGTGCATTGGT-3′, (R) 5′-TCTTTACCGCCTCTGCCTGTAG-3’. DLX2 (F) 5′-TACTCCGCCAAGAGCAGCTATG-3′, (R) 5′-CGAATTTCAGGCTCAAGGTCCTC-3’. SIRT3 (F) 5′-CCCTGGAAACTACAAGCCCAAC-3′, (R) 5′-GCAGAGGCAAAGGTTCCATGAG-3’. GAPDH (F) 5′-GTCTCCTCTGACTTCAACAGCG-3′, (R) 5′-ACCACCCTGTTGCTGTAGCCAA-3’.

## ChIP

The ChIP experiment was carried out according to the protocol of EZ ChIP kit (Merck Millipore,). The cells were cross-linked with 1% formaldehyde for 10 min and terminated with glycine. The cell precipitates were cleaved with SDS lysate, and the DNA in the lysate was cleaved into fragments on a sonicator. Anti-lgG and anti-DLX2 were used for the ChIP assay. The purified DNA products were detected by qRT-PCR to affirm the presence and relative enrichment of SIRT3 in antibody-precipitated DNA.

## Co-IP

The cells were added to the cell lysis buffer and cleaved on ice for 30 min. Then, the extract was incubated with anti-SENP3 or anti-DLX2 antibody at 4 °C overnight. Then, protein A&G beads were added and incubated for 4 h. The coprecipitated protein was washed with SDS loading buffer. Western blot was utilized to confirm the relation between SENP3 and DLX2.

## IP assay

Cells were lysed with IP buffer. The lysate is then incubated overnight with an equal amount of the specified primary antibody at 4 °C and then incubated with A/G magnetic beads. The beads were washed with PBS buffer. Finally, the immunoprecipitated proteins were detected by Western blot.

## Dual-luciferase gene reporter assay

We built pGL3-Basic-SIRT3-WT and pGL3-Basic-SIRT3-MUT luciferase reporter vectors. sh-NC and sh-DLX2 were transfected into 293 T cells with the above plasmids for 48 h. Luciferase activity assay kit (Promega) was utilized to assess the data.

## JASPAR prediction

Potential transcription factor binding sites in the target gene promoter were predicted using JASPAR (https://jaspar.elixir.no/) with default parameters.

## Statistical analyses

SPSS version 19.0 was used for statistical analyses. All data are expressed as mean ± SD. Two-group comparisons were made using the two-tailed unpaired Student’s t-test, while multiple group comparisons were analyzed with one-way ANOVA. All experiments were performed with three technical replicates. For biological replicates, cell-based experiments were conducted with three independent biological replicates, while animal experiments were carried out using six biological replicates. p < 0.05 proved that the difference was statistically significant.

## Results

### Overexpression of SENP3 promoted osteogenic differentiation and inhibited SUMO2/3 modification

**Fig. 1:**
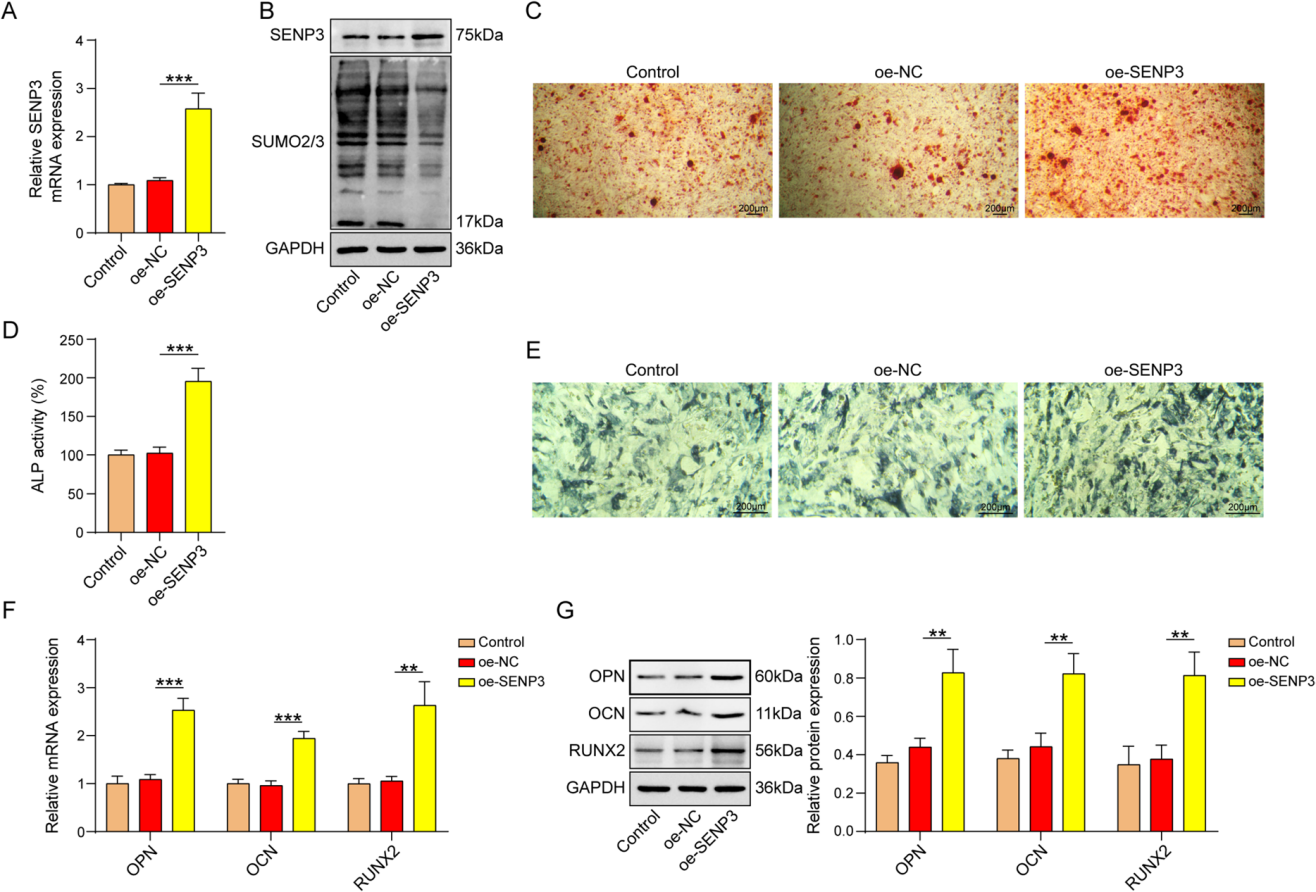
Overexpression of SENP3 promoted osteogenic differentiation and inhibited SUMOylation modification. The experiment was divided into the following groups: Control, oe-NC, and oe-SENP3. **A **qPCR was used to assess SENP3 expression in BMSCs. **B **Western blot was utilized to measure the expression of SENP3 and SUMO2/3 in BMSCs. **C **Alizarin Red staining of BMSCs incubated with osteogenic differentiation medium for 7 days with or without SENP3. **D** and **E** BMSCs were incubated with osteogenic induction medium for 7 days with or without SENP3 for ALP activity detection and ALP staining. **F** and **G** qPCR and western blot evaluation of OPN, OCN, and RUNX2 mRNA and protein levels. *n*=3, **p < 0.01, ***p < 0.001

We induced osteogenic differentiation in BMSCs for 7 days and overexpressed SENP3 in BMSCs to determine whether SENP3 plays a role in the osteogenic differentiation. The overexpression efficiency of SENP3 was validated by qPCR and western blot (Fig. 1A and B). Interestingly, addition of SENP3 declined SUMO2/3 level, indicating that SENP3 might suppress SUMOylation modifications (Fig. 1A and B). Moreover, SENP3 addition accelerated calcium deposit and ALP activity (Fig. 1C-E). Furthermore, OPN, OCN, and RUNX2 mRNA and protein levels were enhanced in SENP3 overexpressed BMSCs (Fig. 1F and G). These data revealed the function of SENP3 in osteogenic differentiation.

## The stability of DLX2 was enhanced by SENP3 through SUMO2/3

**Fig. 2:**
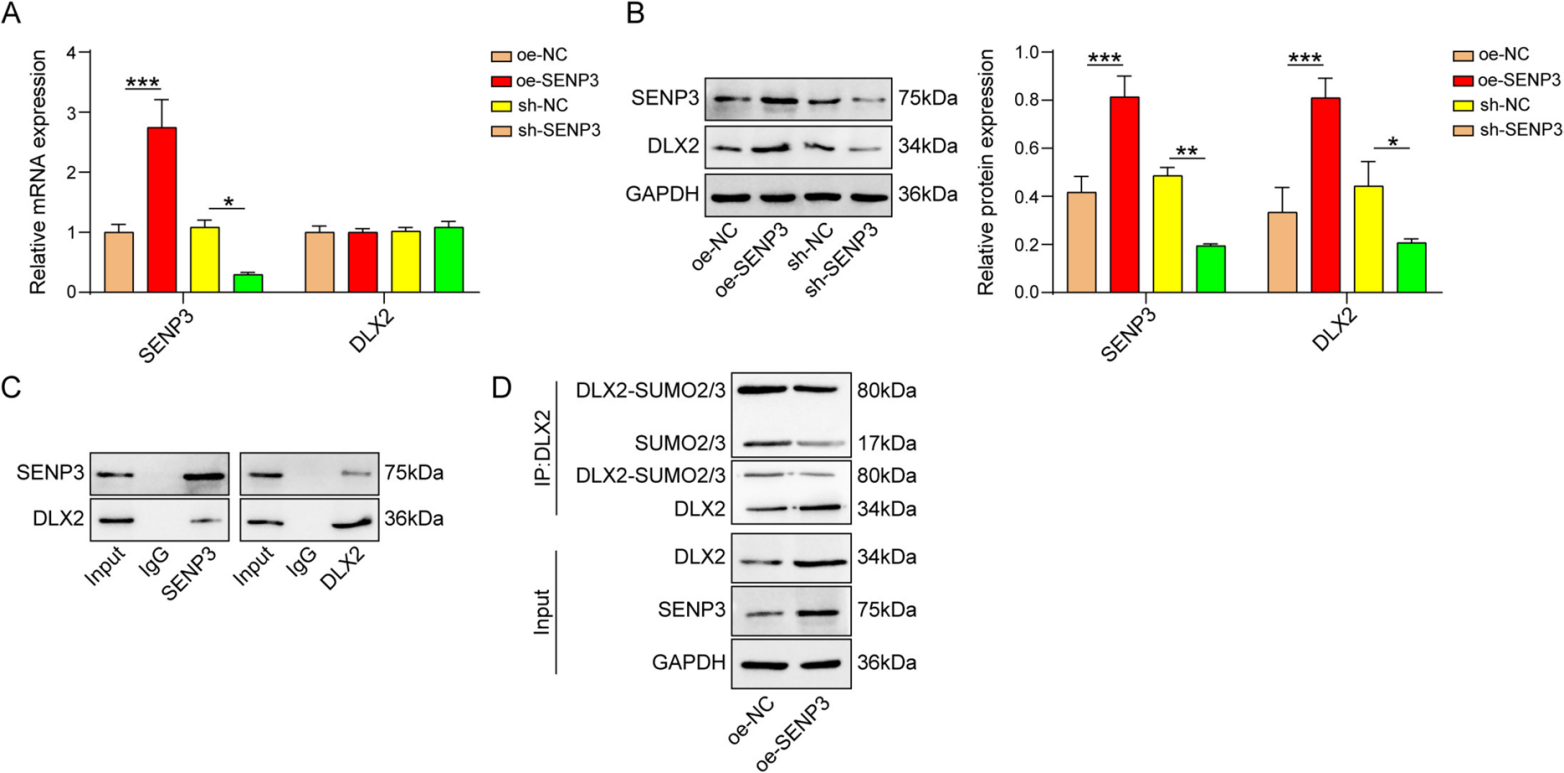
SENP3 influenced the SUMOylation and stability of DLX2. **A** and **B** BMSCs were transfected with oe-NC, oe-SENP3, sh-NC, and sh-SENP3 plasmids. qPCR and western blot were used to measure SENP3 and DLX2 mRNA and protein levels. **C **The binding of SENP3 with DLX2 was testified through Co-IP assay. **D **IP detection of SUMOylated modification levels of DLX2 in oe-NC and oe-SENP3 groups. *n*=3, *p < 0.05, **p < 0.01, ***p < 0.001

Many previous studies have pointed to the contribution of DLX2 to osteogenic differentiation (Zeng et al. 2020; Zhang et al. 2019). Moreover, the presence of multiple sumo sites in DLX2 was predicted by GPS-SUMO (https://sumo.biocuckoo.cn/). Therefore, we examined whether SENP3 could regulate DLX2 expression by altering SUMO modification of DLX2. We first overexpressed and depleted SENP3 in BMSCs and examined the effects on mRNA and protein levels of DLX2. As displayed in Fig. 2A and B, SENP3 addition upregulated and SENP3 knockout downregulated the protein expression of SENP3 and DLX2. Both overexpression and knockdown of SENP3 did not change the mRNA level of DLX2. Then we probed the possible regulation of SENP3 on DLX2 through Co-IP assay. Results indicated that SENP3 interacted with DLX2 in BMSCs (Fig. 2C). Furthermore, we measured post-translational modifications of DLX2 regulated by SENP3 by IP assay. The results showed that in SENP3 overexpressed cells, DLX2 was modified by SUMO 2/3 and the SUMO level of DLX2 was reduced (Fig. 2D). These findings indicated that SENP3 enhanced DLX2 protein level via deSUMOylation of DLX2.

## SENP3 facilitated osteogenic differentiation via regulating DLX2

**Fig. 3:**
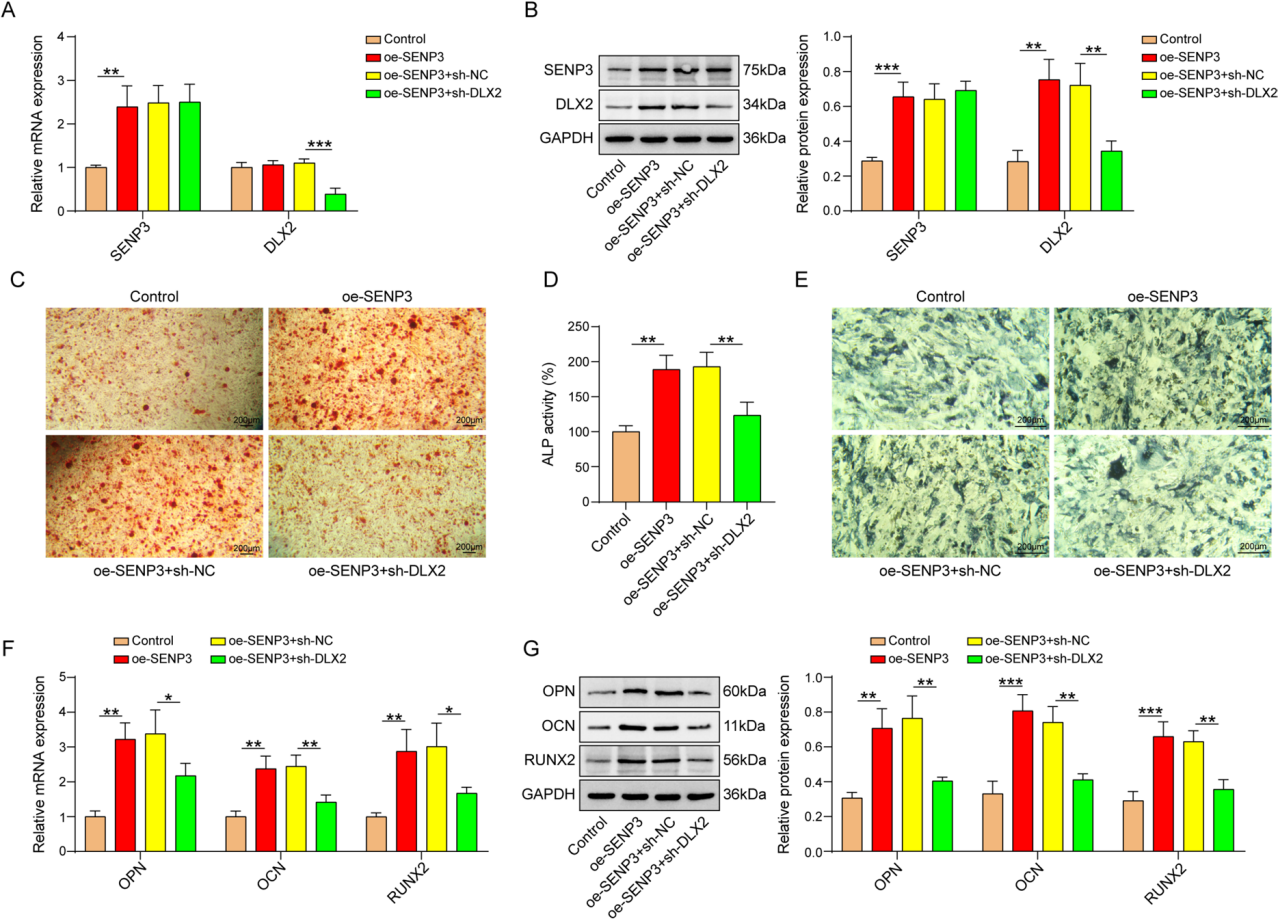
SENP3 facilitated osteogenic differentiation via regulating DLX2. The experiment was divided into the following groups: oe-NC, oe-SENP3, oe-SENP3+shNC, and oe-SENP3+sh-DLX2. **A** and **B** SENP3 and DLX2 mRNA and protein levels were detected by qPCR and western blot. **C **Alizarin Red staining, **D **ALP activity, and **E **ALP staining were performed in BMSCs. **F** and **G** qPCR and western blot evaluation of OPN, OCN, and RUNX2 mRNA and protein levels. *n*=3, *p < 0.05, **p < 0.01, ***p < 0.001

Next, DLX2 was silenced in BMSCs to evaluate its involvement in SENP3-mediated osteogenic differentiation. Overexpression of SENP3 increased SENP3 mRNA level, and did not affect the mRNA level of DLX2. DLX2 knockdown did not affect SENP3 mRNA, but inhibited the mRNA level of DLX2(Fig. 3A). SENP3 overexpression enhanced the protein expression of SENP3 and DLX2, but knockout of DLX2 did not affect the protein level of SENP3 and decreased DLX2 protein level (Fig. 3B). In addition, SENP3 overexpression increased calcium deposit and ALP activity in BMSCs, which was abolished by DLX2 knockdown (Fig. 3C-E). Furthermore, DLX2 reversed the levels of OPN, OCN, and RUNX2 raised by SEBP3 depletion (Fig. 3F and G). Overall, these data demonstrated that SENP3 influenced osteogenic differentiation by regulating DLX2.

## Effect of DLX2 on SIRT3 promoter activity

**Fig. 4:**
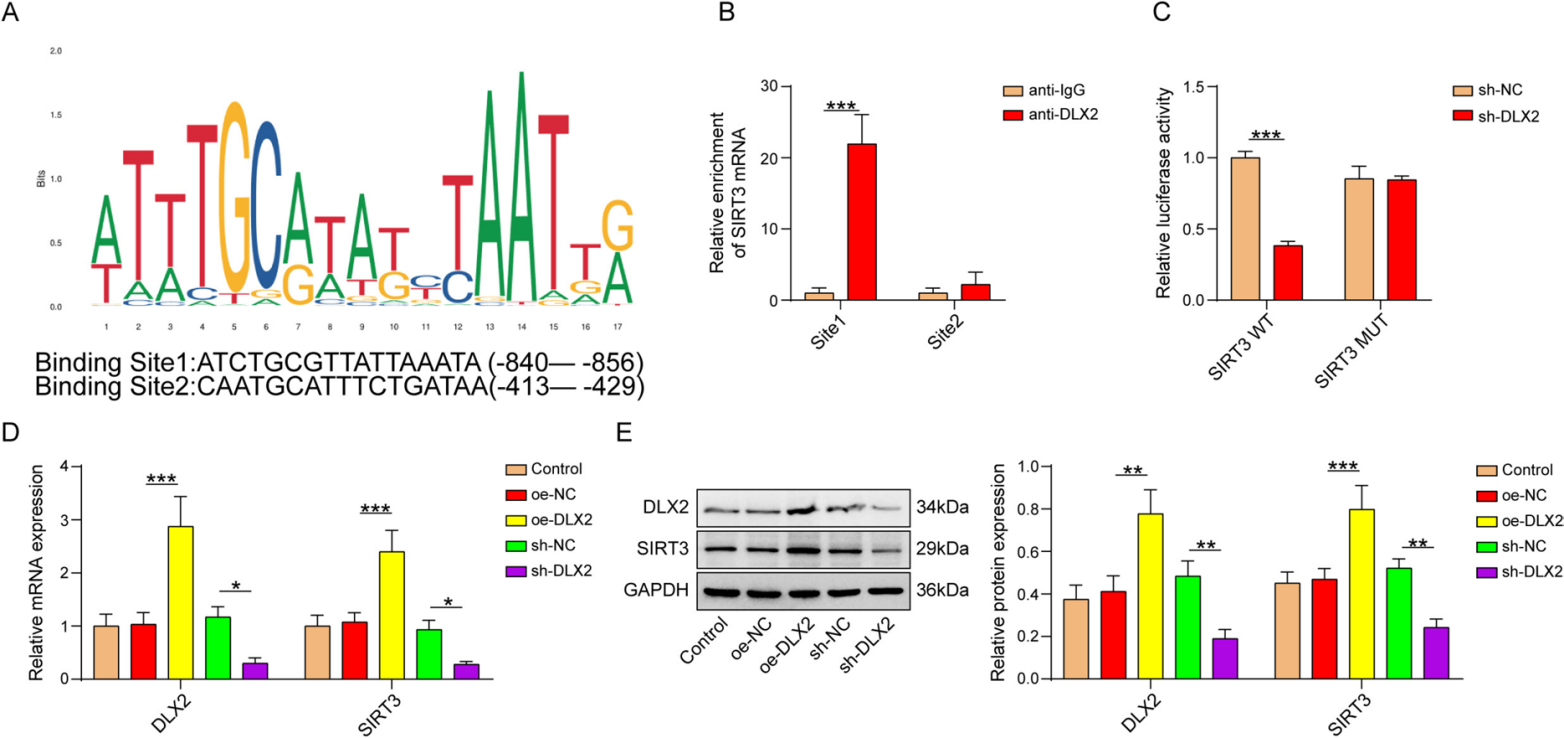
Effect of DLX2 on SIRT3 promoter activity. **A** Jaspar database (https://jaspar.genereg.net/) predicted the binding of DLX2 to the SIRT3 promoter sequence. **B **CHIP detection of the binding of DLX2 to the SIRT3 promoter. **C **Dual-luciferase reporter assay verification of the binding of DLX2 to the SIRT3 promoter; **D** and **E** qPCR and western blot evaluation of DLX2 and SIRT3 mRNA and protein expression in BMSCs transfected with oe-NC, oe-DLX2, sh-NC, and sh-DLX2. *n*=3, *p < 0.05, **p < 0.01, ***p < 0.001

The binding sites (site1: ATCTGCGTTATTAAATA; site2: CAATGCATTTCTGATAA) between DLX2 and SIRT3 promoter predicted by JASPAR database was displayed in Fig. 4A. ChIP assay verified that DLX2 antibodies abundantly enriched in the SIRT3 promoter only at site 1 (Fig. 4B). Moreover, sh-DLX2 markedly suppressed luciferase activity in the SIRT3-WT group, with no change observed in the SIRT3-MUT groups (Fig. 4C). BMSCs were transfected with oe-NC, oe-DLX2, sh-NC or sh-DLX2 plasmids. As qPCR and western blot illustrated, DLX2 overexpression enhanced and DLX2 depletion downregulated DLX2 and SIRT3 mRNA and protein levels (Fig. 4D and E). The results demonstrated that DLX2 directly bound to the SIRT3 promoter to activate SIRT3 transcription.

## DLX2 promoted osteogenic differentiation via regulating SIRT3

**Fig. 5:**
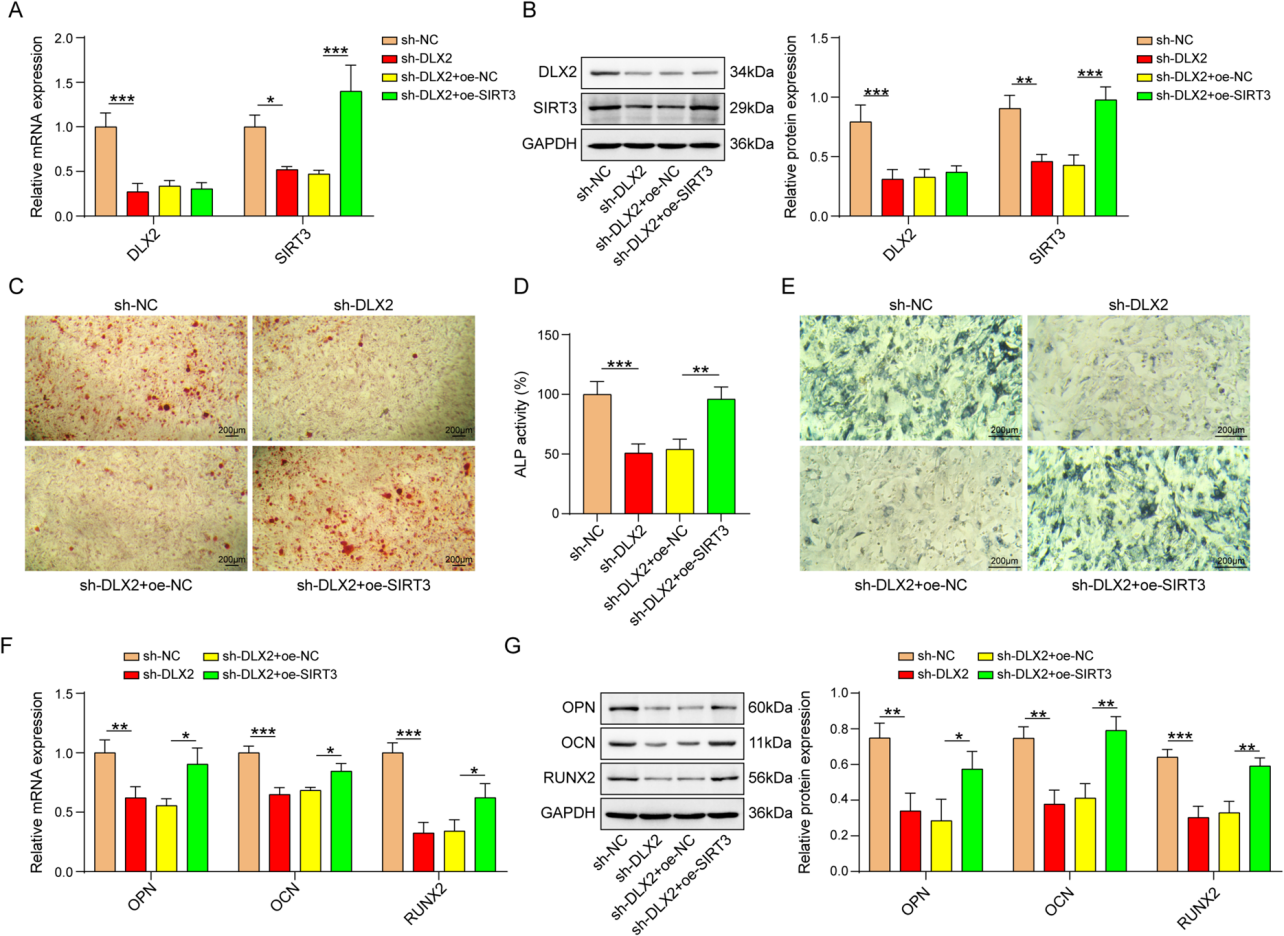
DLX2 promoted osteogenic differentiation via regulating SIRT3. The experiment was divided into the following groups: sh-NC, sh-DLX2, sh-DLX2+oe-SIRT3. **A** and **B** DLX2, and SIRT3 mRNA and protein levels were detected by qPCR and western blot assays. **C** Alizarin Red staining, **D **ALP activity, and **E**ALP staining were performed in BMSCs transfected with plasmids mentioned above. **F** and **G** qPCR and western blot evaluation of OPN, OCN, and RUNX2 mRNA and protein levels. *n*=3, *p < 0.05, **p < 0.01, ***p < 0.001

To investigate whether DLX2 regulates osteogenic differentiation by regulating SIRT3, BMSCs were transfected with sh-DLX2 alone or together with oe-SIRT3. As expounded in Fig. 5A and B, silencing of DLX2 declined mRNA and protein level of DLX2 and SIRT3, and only SIRT3 level was enhanced after transfection with oe-SIRT3. Moreover, DLX2 depletion suppressed calcium deposit and ALP activity, which were reversed by SIRT3 overexpression (Fig. 5C-E). The expression of OPN, OCN, and RUNX2 were decreased after DLX2 knockdown, but the trend were reversed by SIRT3 overexpression (Fig. 5F and G), The results revealed that SIRT3 addition neutralized sh-DLX2-induced repressive effect on osteogenic differentiation.

## SENP3 facilitated osteogenic differentiation by regulating DLX2/SIRT3 in vitro

**Fig. 6:**
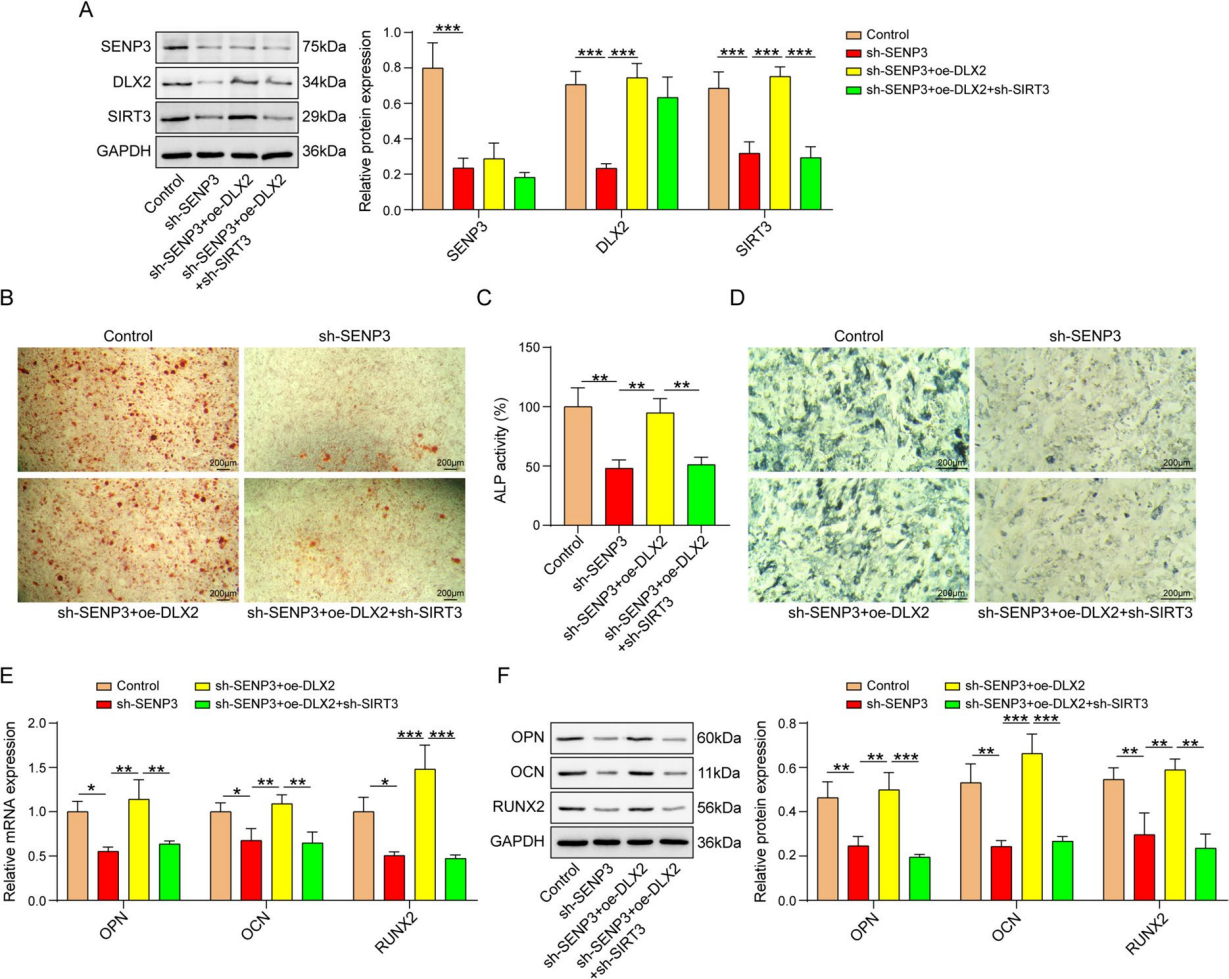
SENP3 was involved in osteogenic differentiation by regulating DLX2/SIRT3 in vitro. The experiment was divided into the following groups: sh-NC, sh-SENP3, sh-SENP3+oe-DLX2, and sh-SENP3+oe-DLX2+sh-SIRT3. **A** and **B** SENP3, DLX2, and SIRT3 mRNA and protein levels were detected by qPCR and western blot assays. **C** Alizarin Red staining, **D **ALP activity, and **E **ALP staining were performed in BMSCs transfected with plasmids mentioned above. **F** and **G** qPCR and western blot evaluation of OPN, OCN, and RUNX2 mRNA and protein levels. *n*=3, *p < 0.05, **p < 0.01, ***p < 0.001

To further affirm whether SENP3 regulated the osteogenic differentiation of BMSCs through DLX2/SIRT3 axis, a rescue experiment was performed by applying DLX2 with or without sh-SIRT3 in SENP3 depleted BMSCs. Western blot results indicated that depletion of SENP3 downregulated SENP3 and SIRT3 protein levels, did not change DLX2 protein level. Overexpression of DLX2 abolished sh-SENP3-induced effect on SIRT3 protein level. SIRT3 knockdown further neutralized DLX2-induced increase on SIRT3 protein level in SENP3 silenced BMSCs (Fig. 6A). SENP3 knockdown inhibited mineralization of extracellular matrix, decreased ALP activity. DLX2 overexpression led to increase in mineralization of extracellular matrix and ALP activity, which were abolished by SIRT3 knockdown (Fig. 6B-D). The levels of osteogenic differentiation markers (OPN, OCN, and RUNX2) were inhibited by SENP3 knockdown. These were raised by DLX2 overexpression, which were reversed by SIRT3 knockdown in BMSCs (Fig. 6E and F). All of the above data attested that SENP3 promoted the osteogenic differentiation of BMSCs through DLX2/SIRT3 axis.

## SENP3 relieved OVX-caused mice osteoporosis

**Fig. 7:**
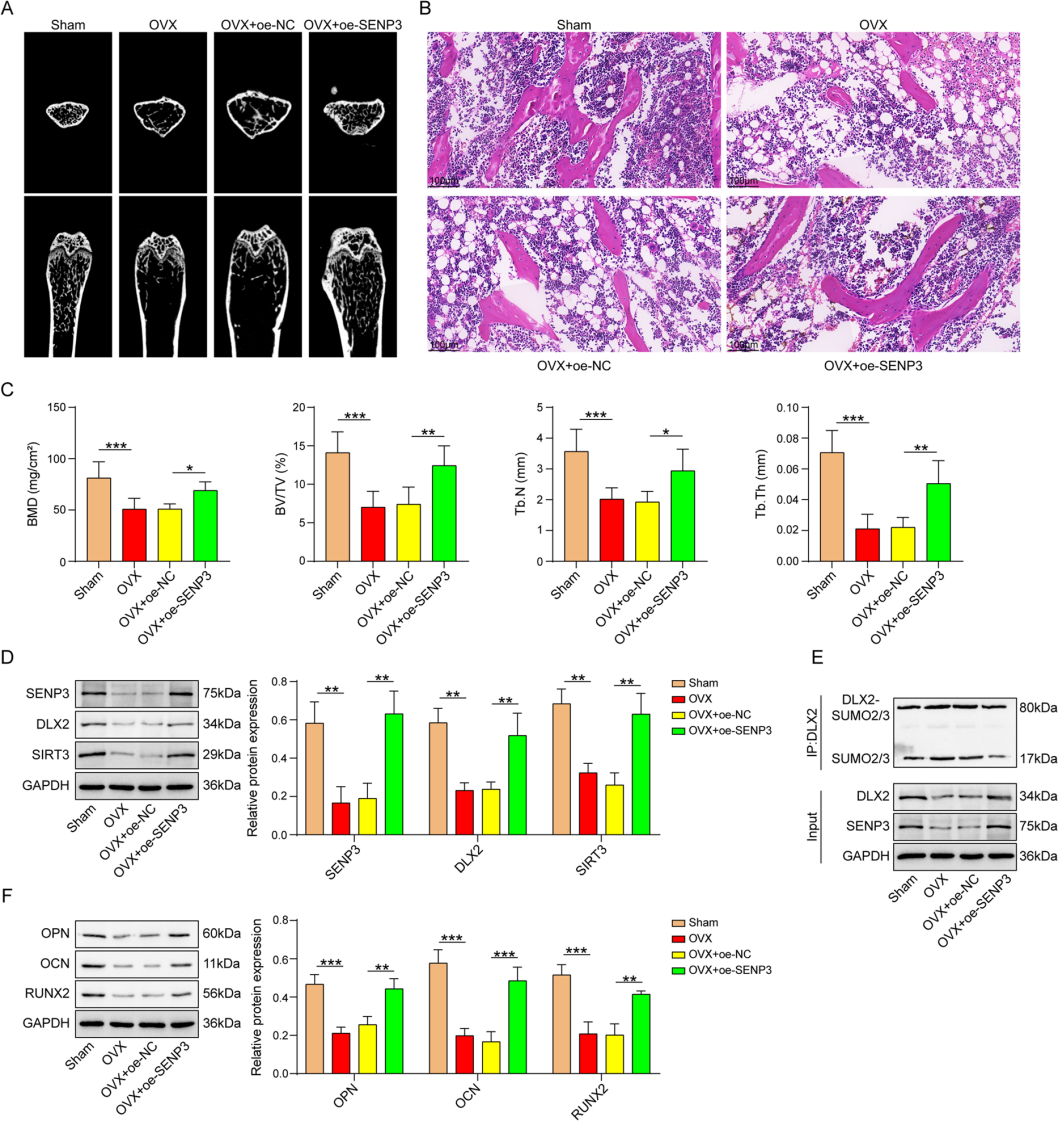
SENP3 relieved OVX-induced mice osteoporosis. The experiment was divided into the following four groups: Sham, OVX, OVX+oe-NC, and OVX+oe-SENP3. **A **Views of mice femur micro-CT. **B **Representative photomicrographs of H&E-stained sections. **C **Microarchitecture analysis of trabecular bone by micro-CT: bone mineral density (BMD), trabecular bone volume fraction (BV/TV), trabecular number (Tb. N), and trabecular thickness (Tb.Th). **D** SENP3, DLX2, and SIRT3 protein levels were detected by western blot. **E** IP assay was used to measure SUMOylated modification levels of DLX2. **F **OPN, OCN, and RUNX2 protein levels were measured by western blot. *n*=6, *p < 0.05, **p < 0.01, ***p < 0.001

To probe the influence of SENP3 on bone loss in OP mice, we conducted an OP mouse model by OVX surgery and injected AVV-SENP3 through the tail vein. Results of Micro-CT (Fig. 7A) and HE (Fig. 7B) of femur uncovered that the bone trabeculae of Sham group were evenly distributed, arranged regularly, and interwoven. The shape of bone trabeculae in group OVX is slender, the arrangement is loose, and the number and density of bone trabeculae are significantly reduced. Compared with OVX group, the number and shape of bone trabeculae were significantly improved due to overexpression of SENP3. Moreover, Microarchitecture analysis indicated BMD, BV/TV, Tb.N, and Tb.Th were evidently declined in OVX mice compared with sham group. After injected with SENP3, most of these modified parameters, which reflect changes in bone mass or bone microstructure, were substantially reversed (Fig. 7C). Furthermore, SENP3, DLX2, and SIRT3 protein levels were decreased in OVX group, which were abolished by SENP3 (Fig. 7D). Moreover, SUMO-modified DLX2 was dramatically enhanced in OVX mice, which was recovered by SENP3 overexpression (Fig. 7E). After injected AVV-SENP3, the reduced levels of OPN, OCN, and RUNX2 protein were reversed in OVX mice (Fig. 7F). These results suggested that SENP3 improves bone biomechanical properties and bone microenvironment. Altogether, these results implied that SENP3 could prevent bone loss via regulating DLX2/SIRT3 axis in OVX mice.

## Discussion

Bone undergoes continuous remodeling, with osteoblasts mediating bone formation (Li et al. 2022). BMSCs in bone marrow stroma serve as the primary osteoblast source, capable of differentiating into multiple functional cell types under specific conditions (You et al. 2023). Accelerating BMSC osteogenic differentiation of is very important for the treatment of metabolic bone diseases such as osteoporosis (Guo, Y.c,, et al. 2020). However, further investigation is needed to explain specific mechanisms regulating osteogenic differentiation of BMSCs. In this work, we performed in vitro and in vivo assays to evaluate the potential effect of SENP3 in osteogenic differentiation and the pathogenesis of osteoporosis. Our data expounded addition of SENP3 accelerated osteogenic differentiation in vitro and alleviated osteoporosis in vivo by modulating the DLX2/SIRT3 axis.

SENP3, alters protein modifications by coupling target proteins. Under pathological conditions, changes in SENP3 levels affect the SUMOlyation of proteins, leading to abnormal cell activity and cellular responses, as well as the occurrence of human diseases, such as neurological disorders (Wang et al. 2017), cardiovascular and cerebrovascular diseases (Guo et al. 2013; Rawlings et al. 2019), and cancers (Wang et al. 2024). Moreover, SENP3 accelerated osteogenic differentiation by enhancing HOX level via de-SUMOylating RbBP5 in dental follicle stem cells (Nayak et al. 2014). SENP3 regulated osteogenic differentiation by regulating MLL1/MLL2 methyltransferase complexes (Nayak et al. 2014). However, there is little evidence to directly discuss the function of SENP3 in osteoporosis. This work revealed that SENP3 expression was declined in OVX mice. Besides, the study of Wang et al. illustrated that SENP3 addition alleviates bone loss in type II diabetes-caused osteoporotic rats (Wang et al. 2023). Similarly, this study elucidated that SENP3 prevents bone loss, alters bone metabolism, promotes bone formation in OVX mice. Moreover, SENP3 addition accelerated osteogenic differentiation of BMSCs, reflecting the stimulative effects on calcium deposit, ALP activity, and level of osteogenic genes.

Distal-less gene and its homologous gene Dlx, as homologous domain transcription factors, play an important role in distal limb development in animal kingdom (Hiruta et al. 2014). The Dlx gene family consists of six members (DLX1-6). Among them, it has been illustrated that DLX2 addition promotes early osteogenic differentiation by directly enhancing ALP, and promotes late osteogenic differentiation by directly increasing OCN (Zhang et al. 2019). Zeng et al. revealed that DLX2 facilitates BMSCs osteogenic differentiation via activating Wnt/β-catenin pathway (Zeng et al. 2020). There are similarities with previous studies. We observed that DLX2 depletion suppressed osteogenic differentiation, reflecting the repressive effects on calcium deposit, ALP activity, and expression of osteogenic genes. Moreover, DLX2 level was declined in OVX mice. Interestingly, Duverger et al. found that SUMOylation of DLX3 via SUMO1 enhances its transcriptional activity, which plays a key role in bone and tooth development (Duverger et al. 2011). In this work, we predicted from the SUMO website that DLX2 has SUMO sites. We first uncovered that SENP3 increased DLX2 stability by SUMO2/3. Addition of SENP3 increased DLX2 protein levels and has not change in mRNA level of DLX2. Moreover, DLX2 silencing did not alter the mRNA and protein levels of SENP3. Furthermore, DLX2 knockdown neutralized sh-SENP3-caused prohibitive influences on BMSCs osteogenic differentiation.

SIRT3, a member of the sirtuin family, has been confirmed as a positive factor in bone remodeling (Zheng et al. 2021). For instance, Huh et al. discovered that SIRT3 keeps bone homeostasis via modulating AMPK-PGC-1β signaling in mice (Ding et al. 2017). Zheng et al. elucidated that SIRT3 treatment can slow the bone destruction stimulated by titanium particles and enhance bone formation via GSK-3β/β-catenin signaling (Zheng et al. 2021). Liu et al. revealed that SIRT3 improves osteoporosis and combats BMSC aging by stabilizing mitochondrial homeostasis and heterochromatin. Similarly, we also revealed a beneficial effect of SIRT3 on osteoporosis. We found that SIRT3 was decreased in OVX mice in vivo and facilitated BMSC osteogenic differentiation in vitro. Moreover, we identified a novel mechanism by which SIRT3 is implicated in the progression of osteoporosis. Previous studies have pointed out DLX2 activates Wnt1 transcription promote osteogenic differentiation of hBMSCs (Zeng et al. 2020). Here, DLX2 served as a transcription factor of SIRT3 and accelerated SIRT3 transcription. SIRT3 addition neutralized sh-DLX2-induced suppressive effect on osteogenic differentiation.

In this study, hBMSCs were used for in vitro assays, whereas a mouse model was employed for in vivo validation. This experimental design was chosen based on both practical considerations and scientific rationale. hBMSCs possess well-characterized osteogenic differentiation potential and are widely used to explore osteogenesis-related molecular mechanisms due to their closer relevance to human bone biology. Using hBMSCs enhances the translational value of our findings and allows us to investigate the role of SENP3 in a context that better reflects potential clinical applications. On the other hand, the mouse model provides an essential in vivo platform to evaluate the systemic and physiological effects of SENP3 modulation on bone mass and architecture, which cannot be fully assessed in vitro. Although there are species-specific differences, the key regulatory pathways involved in osteogenic differentiation, including the SENP3–DLX2–SIRT3 axis and SUMOylation mechanisms, are evolutionarily conserved between humans and mice. Thus, we believe that this combined approach is appropriate and enables a comprehensive understanding of SENP3 function in both cellular and organismal contexts.

There are several limitations in this study. First, although we used human BMSCs for in vitro experiments and a mouse model for in vivo validation, interspecies variations could limit translational relevance. Second, while we demonstrated the regulatory role of SENP3 in osteogenic differentiation via the DLX2/SIRT3 axis and SUMO2/3-mediated stabilization, other downstream pathways or interacting proteins may also be involved and remain to be explored.

In summary, the present work illustrated that SENP3 mitigates osteoporosis through promoting SIRT3 transcription by increase of DLX2 stability via SUMO2/3. This work provides a new perspective for the treatment of osteoporosis.

## Supplementary Information

Below is the link to the electronic supplementary material.

Supplementary file1 (DOCX 20246 KB)

Supplementary file2 (XLSX 20 KB)

## Data Availability

The datasets used or analyzed during the current study are available from the corresponding author on reasonable request.

## References

[CR1] Chen F, Ma D, Li A. SENP3 regulates high glucose-induced endothelial dysfunction via ROS dependent signaling. Diab Vasc Dis Res. 2020;17(6):1479164120970895. 33231124 10.1177/1479164120970895 PMC7919223

[CR2] Chen, J., et al., *SENP3 attenuates foam cell formation by deSUMOylating NLRP3 in macrophages stimulated with ox-LDL.* Cellular Signalling, 2024: p. 111092. 10.1016/j.cellsig.2024.111092 38331013

[CR3] de Melo J, et al. Dlx2 homeobox gene transcriptional regulation of Trkb neurotrophin receptor expression during mouse retinal development. Nucleic Acids Res. 2008;36(3):872–84. 18086710 10.1093/nar/gkm1099 PMC2241891

[CR4] Depew MJ, Lufkin T, Rubenstein JL. Specification of jaw subdivisions by genes. Science. 2002;298(5592):381. 12193642 10.1126/science.1075703

[CR5] Ding Y, et al. Sirtuin 3 is required for osteogenic differentiation through maintenance of PGC-1ɑ-SOD2-mediated regulation of mitochondrial function. Int J Biol Sci. 2017;13(2):254. 28255277 10.7150/ijbs.17053 PMC5332879

[CR6] Duverger O, et al. SUMOylation of DLX3 by SUMO1 promotes its transcriptional activity. J Cell Biochem. 2011;112(2):445–52. 21268066 10.1002/jcb.22891 PMC3180851

[CR7] Guo C, et al. SENP3-mediated deSUMOylation of dynamin-related protein 1 promotes cell death following ischaemia. EMBO J. 2013;32(11):1514–28. 23524851 10.1038/emboj.2013.65 PMC3671254

[CR8] Guo Y, et al. Sirt3-mediated mitophagy regulates AGEs-induced BMSCs senescence and senile osteoporosis. Redox Biol. 2021;41:101915. 33662874 10.1016/j.redox.2021.101915 PMC7930642

[CR9] Guo YC, et al. Ubiquitin-specific protease USP 34 controls osteogenic differentiation and bone formation by regulating BMP 2 signaling. The EMBO journal. 2018;37(20):e99398. 30181118 10.15252/embj.201899398 PMC6187217

[CR10] Guo YC, et al. Ubiquitin-specific protease USP 34 controls osteogenic differentiation and bone formation by regulating BMP 2 signaling. The EMBO Journal. 2020;39(20):e105578. 33058248 10.15252/embj.2020105578 PMC7560212

[CR11] Hiruta C, et al. Targeted gene disruption by use of transcription activator-like effector nuclease (TALEN) in the water flea Daphnia pulex. BMC Biotechnol. 2014;14:1–8. 10.1186/s12896-014-0095-7 PMC4239399 25404042

[CR12] Kim B-J, et al. Osteoclast-secreted SLIT3 coordinates bone resorption and formation. J Clin Investig. 2018;128(4):1429–41. 29504949 10.1172/JCI91086 PMC5873876

[CR13] Li C, et al. The osteoprotective role of USP26 in coordinating bone formation and resorption. Cell Death Differ. 2022;29(6):1123–36. 35091692 10.1038/s41418-021-00904-x PMC9177963

[CR14] Liu Y, et al. Role of non-canonical post-translational modifications in gastrointestinal tumors. Cancer Cell Int. 2023;23(1):225. 37777749 10.1186/s12935-023-03062-x PMC10544213

[CR15] Nayak A, et al. The SUMO-specific isopeptidase SENP3 regulates MLL1/MLL2 methyltransferase complexes and controls osteogenic differentiation. Mol Cell. 2014;55(1):47–58. 24930734 10.1016/j.molcel.2014.05.011

[CR16] Rawlings N, et al. Protective role of the deSUMOylating enzyme SENP3 in myocardial ischemia-reperfusion injury. PLoS ONE. 2019;14(4): e0213331. 30973885 10.1371/journal.pone.0213331 PMC6459529

[CR17] Shen G, et al. Foxf1 knockdown promotes BMSC osteogenesis in part by activating the Wnt/β-catenin signalling pathway and prevents ovariectomy-induced bone loss. EBioMedicine, 2020. 52. 10.1016/j.ebiom.2020.102626 PMC6992955 31981979

[CR18] Wang C, et al. Overexpression of SENP3 promotes PPAR-γ transcription through the increase of HIF-1α stability via SUMO2/3 and participates in molecular mechanisms of osteoporosis. Mol Cell Endocrinol. 2023;577: 112014. 37473957 10.1016/j.mce.2023.112014

[CR19] Wang D-P, et al. Inhibition of SENP3 by URB597 ameliorates neurovascular unit dysfunction in rats with chronic cerebral hypoperfusion. Biomed Pharmacother. 2017;91:872–9. 28501776 10.1016/j.biopha.2017.05.021

[CR20] Wang L, Li J, Yu C. SENP3 aggravates renal tubular epithelial cell apoptosis in lipopolysaccharide-induced acute kidney injury via deSUMOylation of Drp1. Kidney Diseases. 2022;8(5):424–36. 36466072 10.1159/000525308 PMC9710481

[CR21] Wang P, et al. SENP3 mediates the activation of the Wnt/β-catenin signaling pathway to accelerate the growth and metastasis of oesophagal squamous cell carcinoma in mice. Funct Integr Genomics. 2024;24(2):1–10. 10.1007/s10142-024-01321-2 38383667

[CR22] Yau T-Y, et al. SUMO interacting motifs: structure and function. Cells. 2021;10(11):2825. 34831049 10.3390/cells10112825 PMC8616421

[CR23] Yau T-Y, Molina O, Courey AJ. SUMOylation in development and neurodegeneration. Development. 2020;147(6):dev175703. 32188601 10.1242/dev.175703 PMC7097199

[CR24] You Y, et al. Ortho-silicic acid enhances osteogenesis of osteoblasts through the upregulation of miR-130b which directly targets PTEN. Life Sci. 2021;264:118680. 33130075 10.1016/j.lfs.2020.118680

[CR25] You Y, et al. WTAP-mediated m6A modification modulates bone marrow mesenchymal stem cells differentiation potential and osteoporosis. Cell Death Dis. 2023;14(1):33. 36650131 10.1038/s41419-023-05565-x PMC9845239

[CR26] Zeng C, et al. Galangin mitigates glucocorticoid-induced osteoporosis by activating autophagy of BMSCs via triggering the PKA/CREB signaling pathway: Galangin mitigates glucocorticoid-induced osteoporosis. Acta Biochim Biophys Sin. 2023;55(8):1275. 37365870 10.3724/abbs.2023063 PMC10448057

[CR27] Zeng X, et al. DLX2 activates Wnt1 transcription and mediates Wnt/β-catenin signal to promote osteogenic differentiation of hBMSCs. Gene. 2020;744: 144564. 32165291 10.1016/j.gene.2020.144564

[CR28] Zhang J, et al. Overexpression of Dlx2 enhances osteogenic differentiation of BMSCs and MC3T3-E1 cells via direct upregulation of Osteocalcin and Alp. Int J Oral Sci. 2019;11(2):12. 30880332 10.1038/s41368-019-0046-1 PMC6421343

[CR29] Zhang Y, et al. SENP3 suppresses osteoclastogenesis by de-conjugating SUMO2/3 from IRF8 in bone marrow-derived monocytes. Cell Reports. 2020;30(6):1951-1963. e4. 32049023 10.1016/j.celrep.2020.01.036

[CR30] Zheng K, et al. Protective effects of sirtuin 3 on titanium particle-induced osteogenic inhibition by regulating the NLRP3 inflammasome via the GSK-3β/β-catenin signalling pathway. Bioactive Materials. 2021;6(10):3343–57. 33817415 10.1016/j.bioactmat.2021.02.039 PMC8005659

