# Supplementary material for: SENP3 alleviates osteoporosis via promoting SIRT3 transcription through the increase of DLX2 stability via SUMO2/3
Source: Cell Biol Toxicol. 2025 Jun 10;41(1):99. doi: 10.1007/s10565-025-10052-4 (PMC12152034; doi:10.1007/s10565-025-10052-4)
Supplement: Supplementary file 1 — Supplementary file1 (DOCX 20246 KB) [file 10565_2025_10052_MOESM1_ESM.docx]

FIG 1B

SENP3


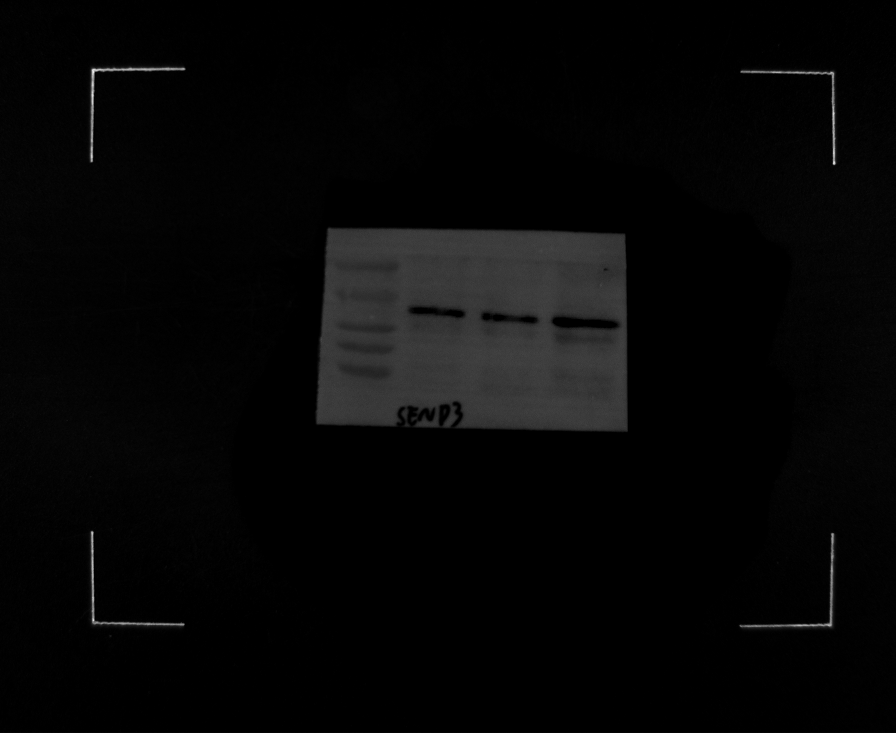

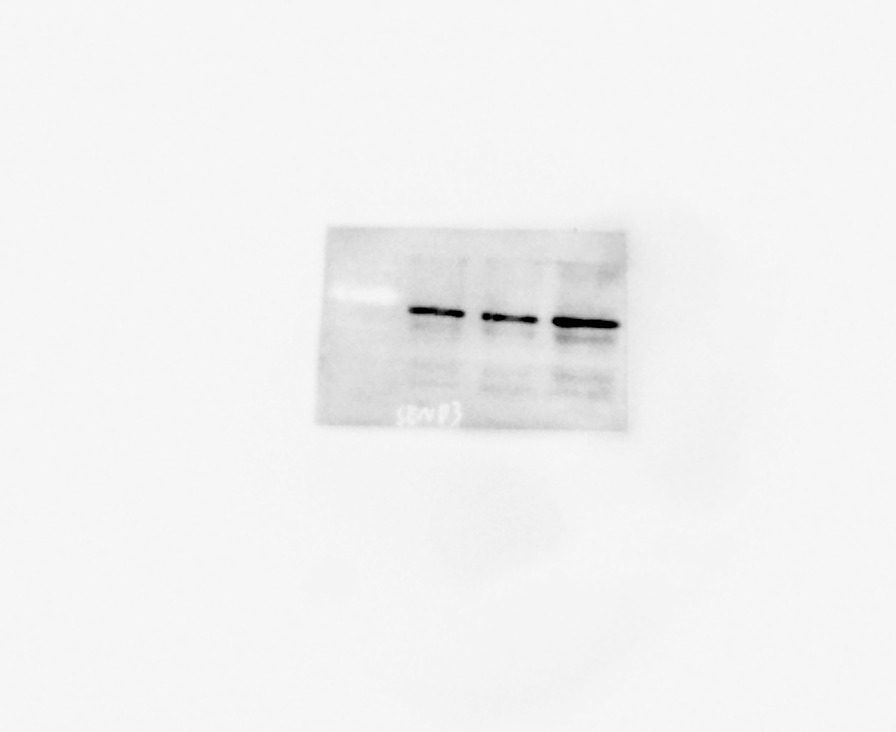


SUMO2/3


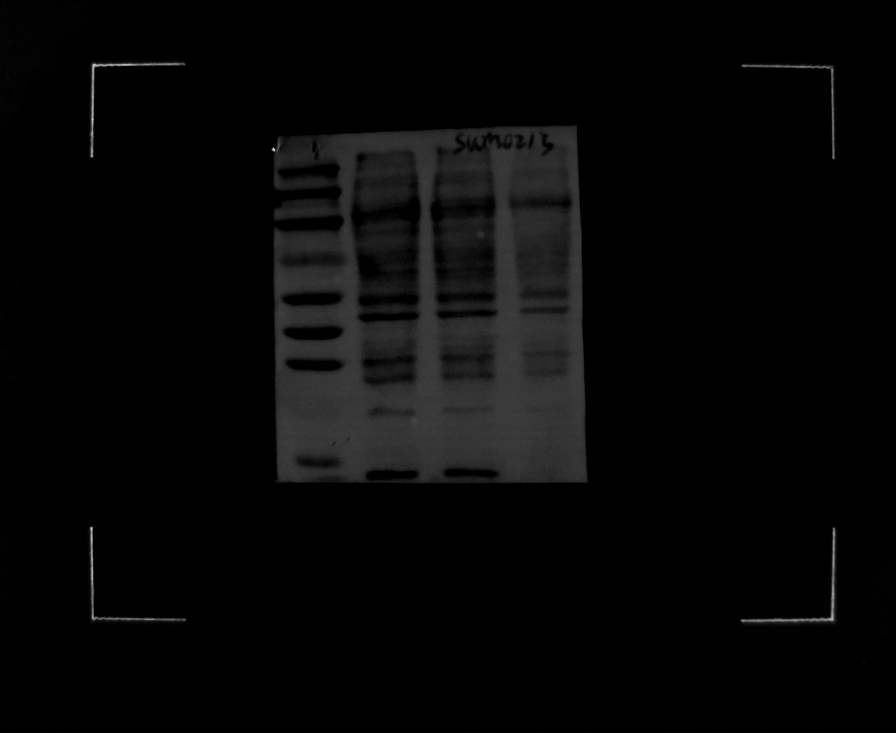

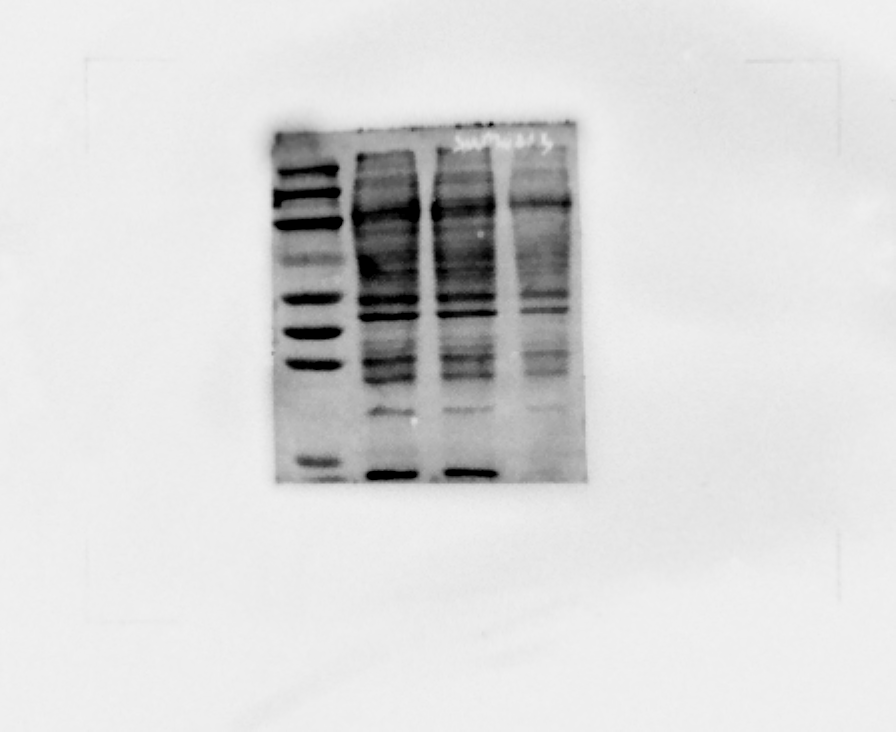


GAPDH


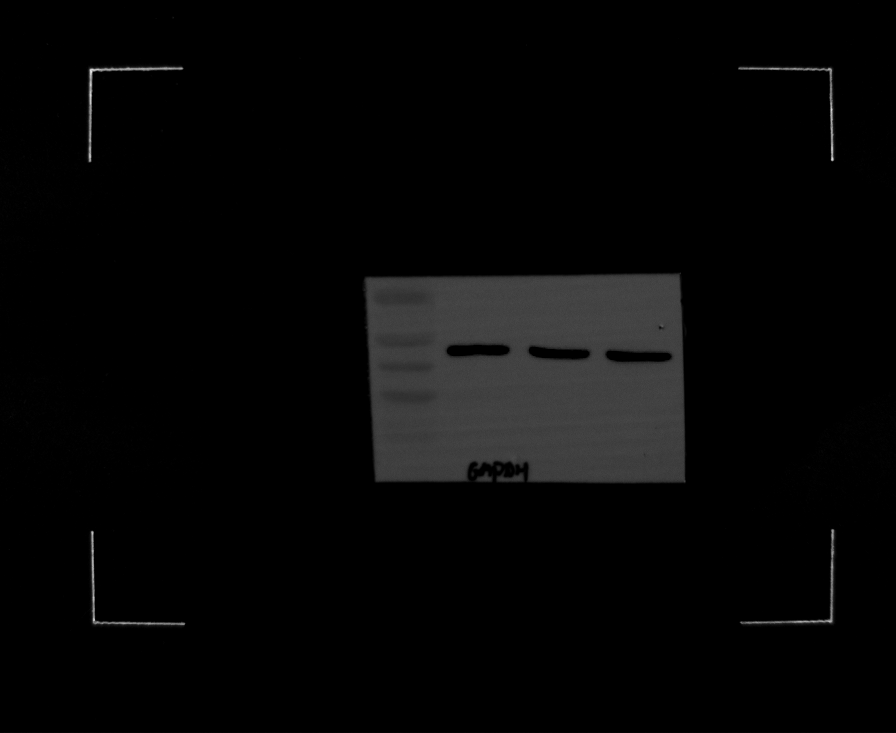

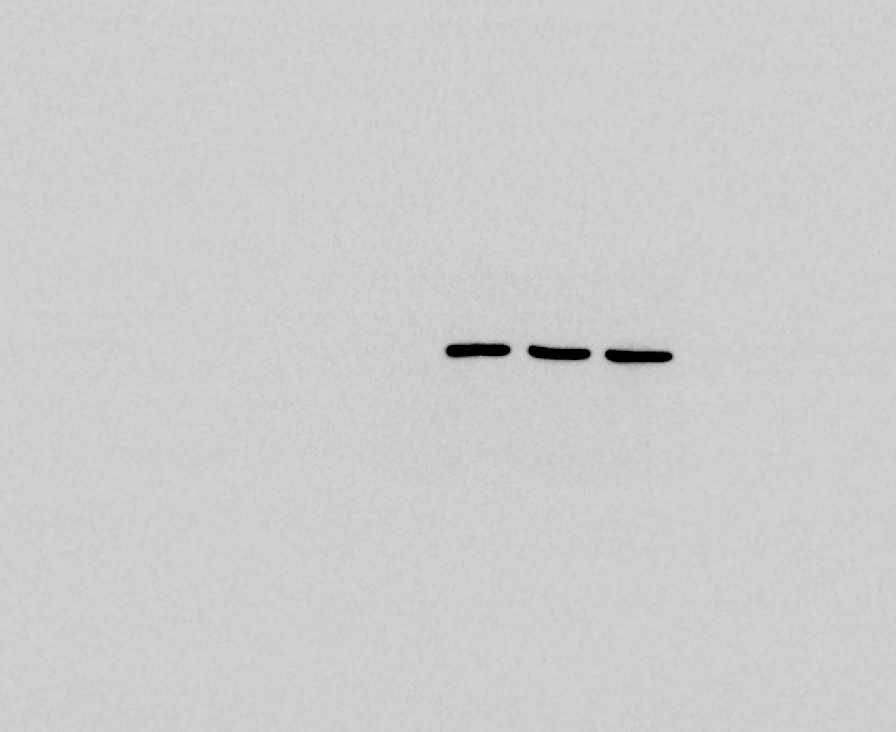


FIG 1G

OPN


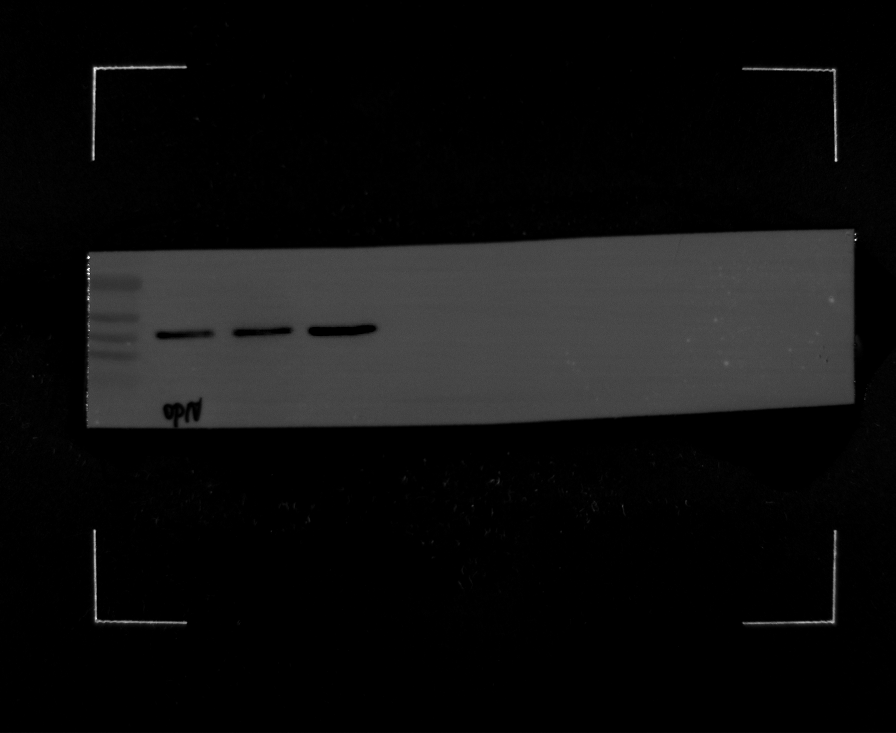

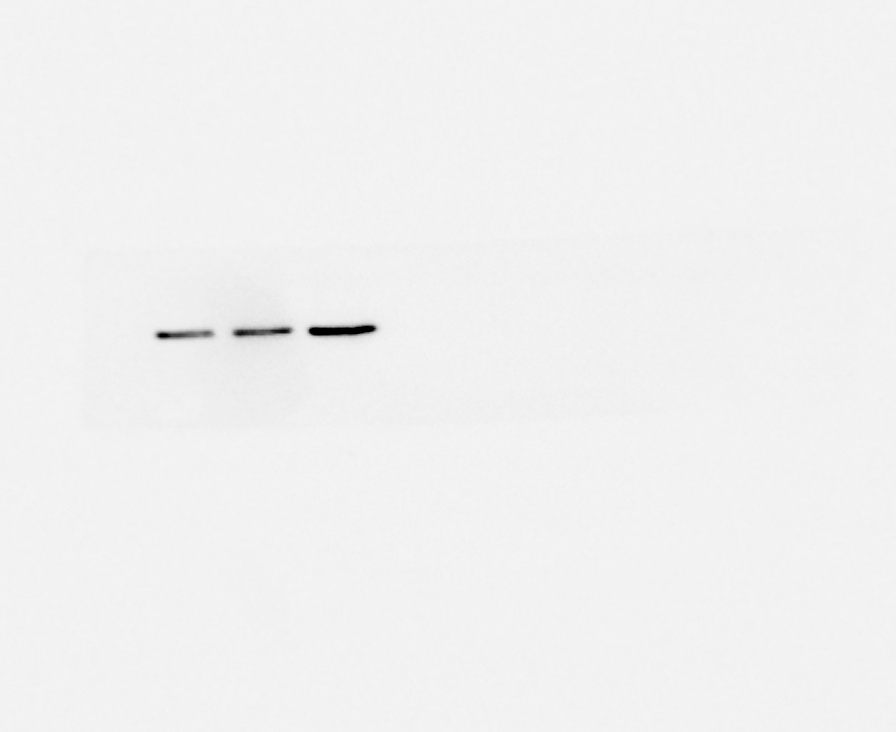


OCN


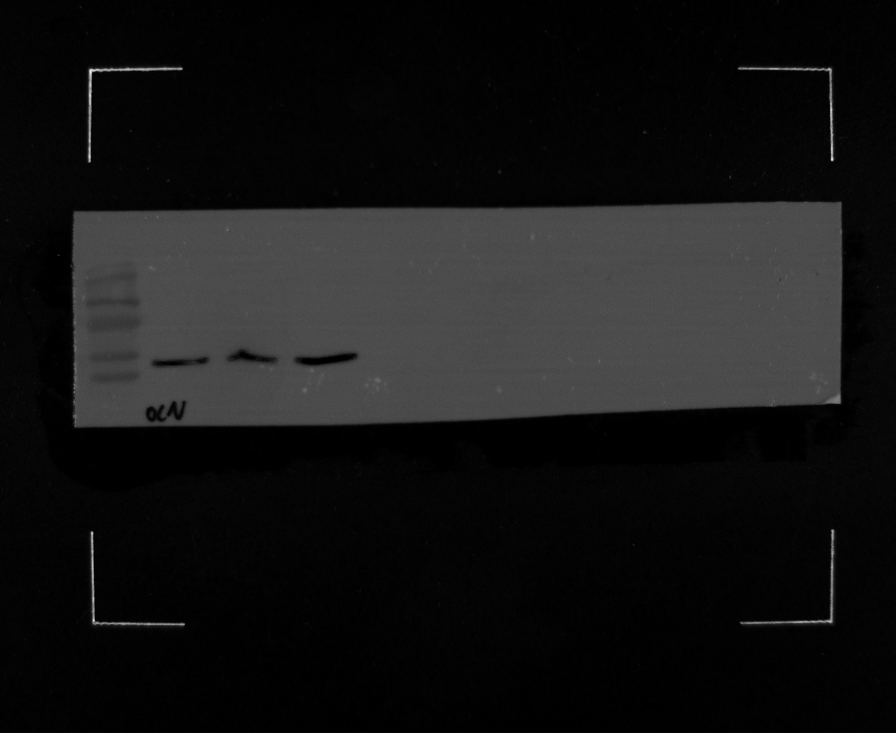

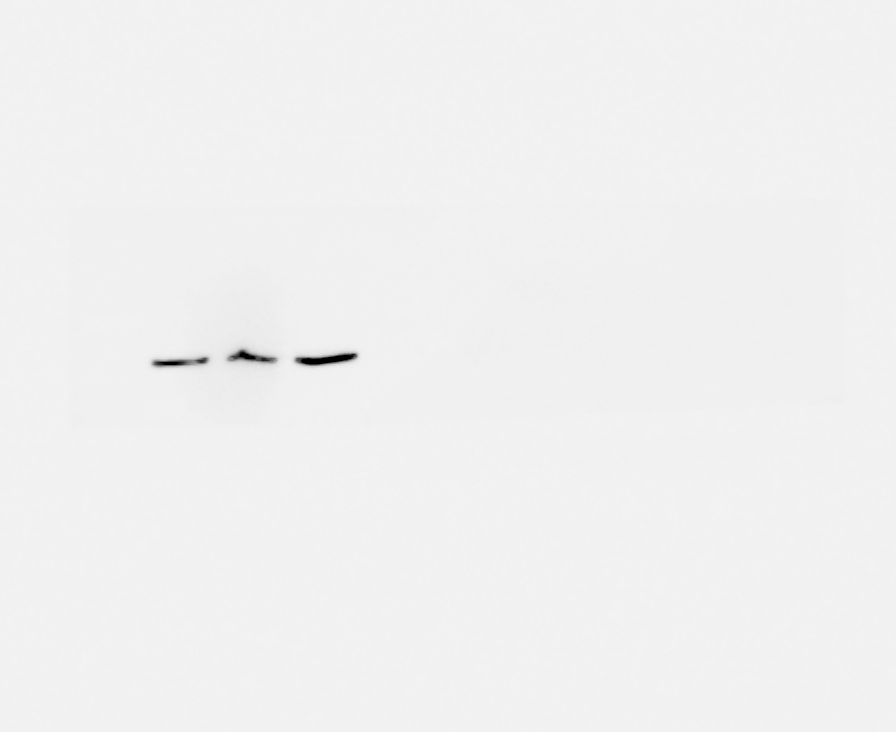


RUNX2


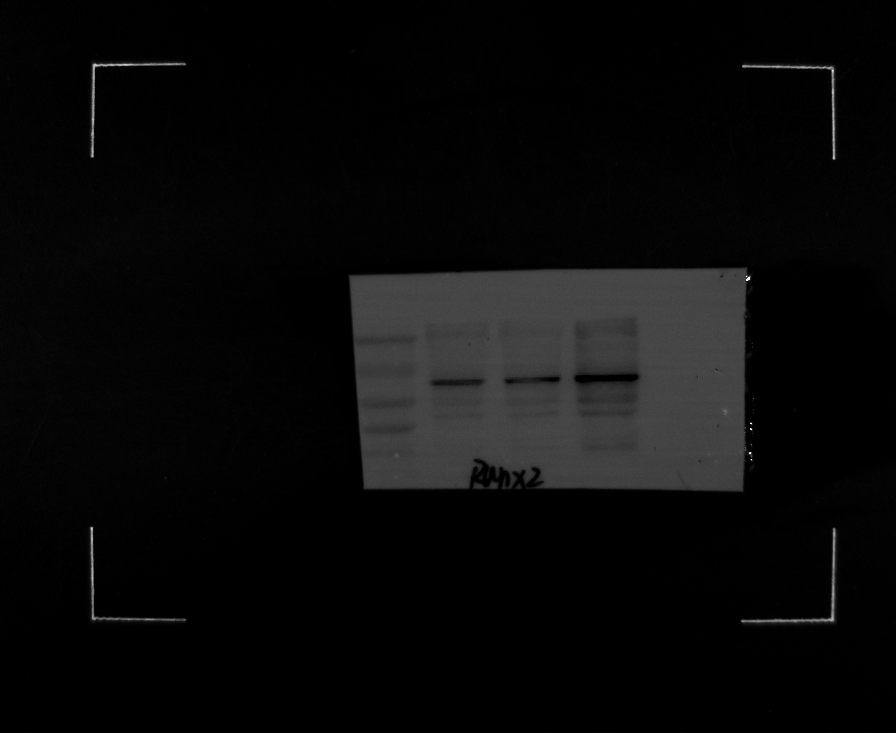

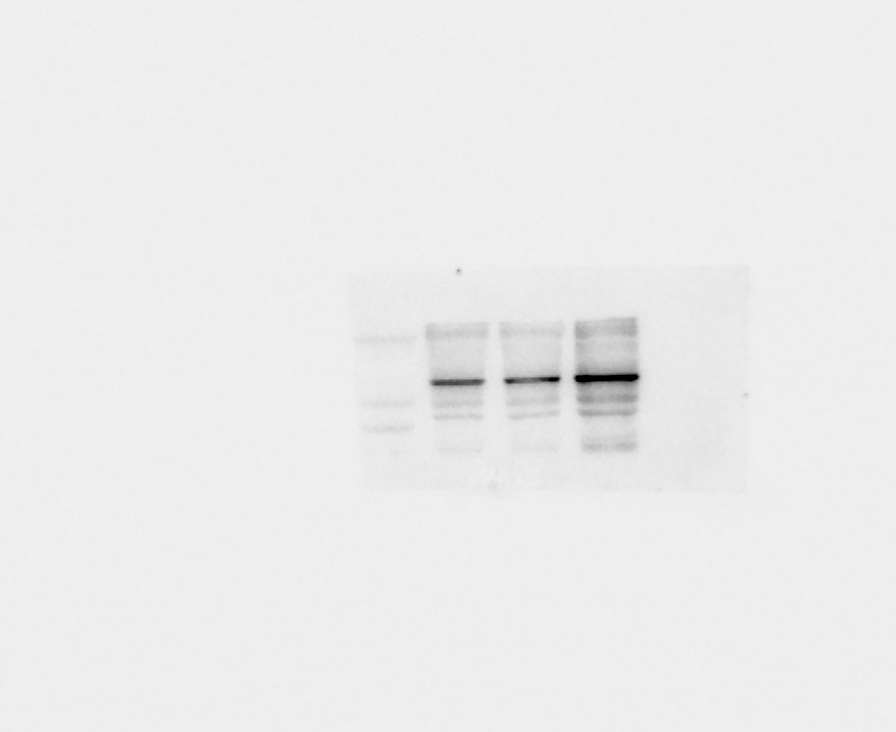


GAPDH


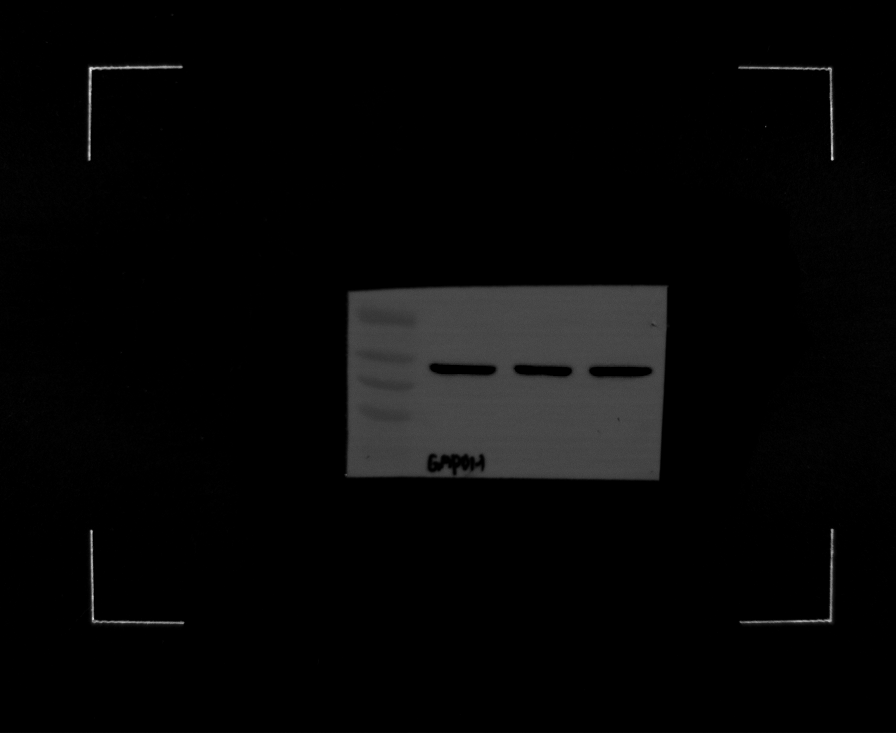

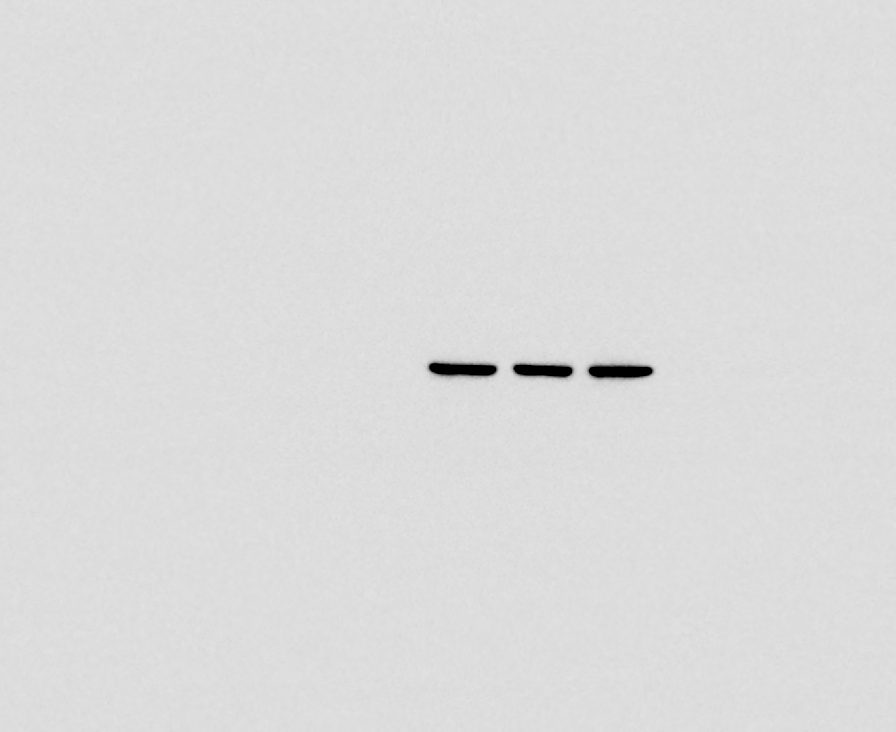


FIG 2B

SENP3


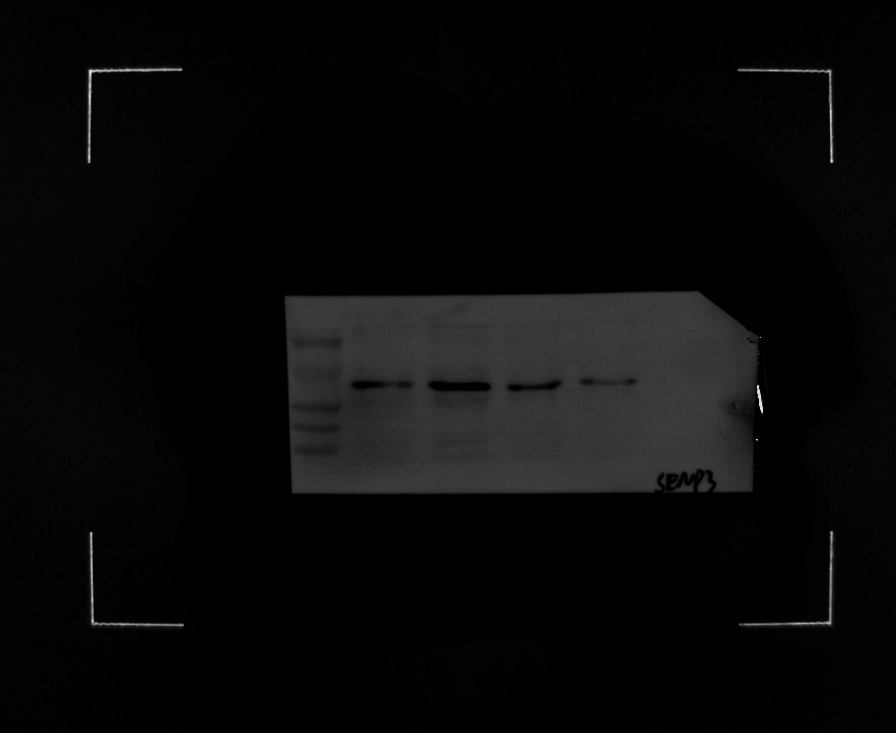

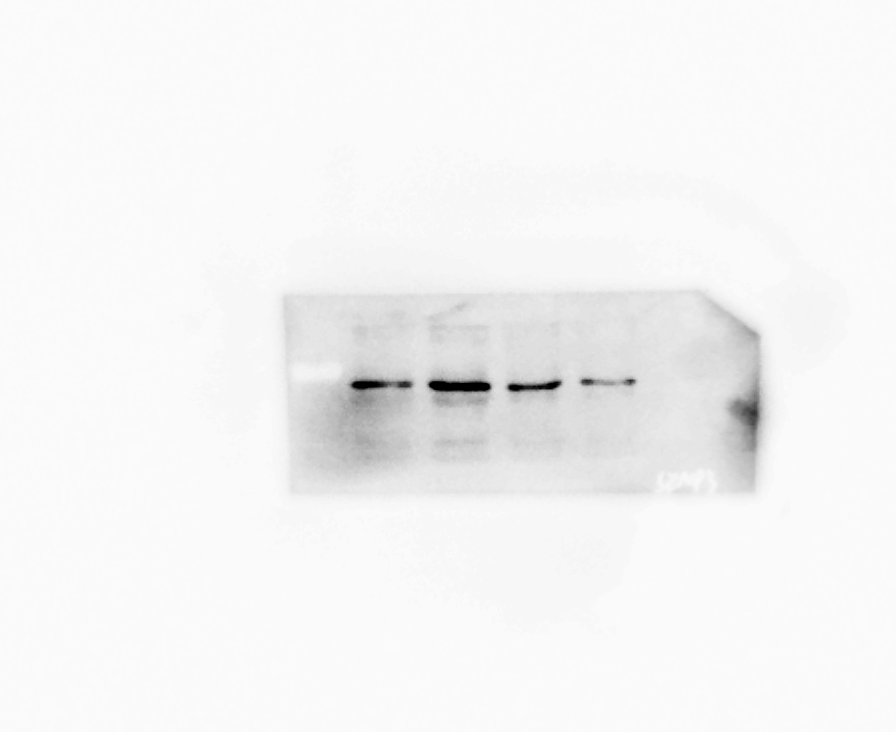


DLX2


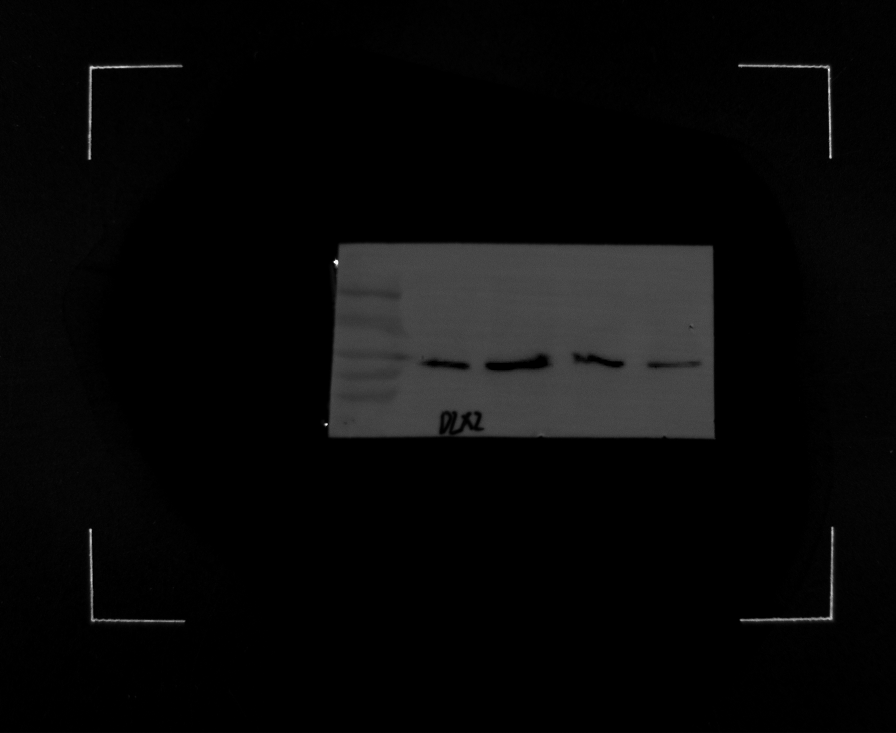

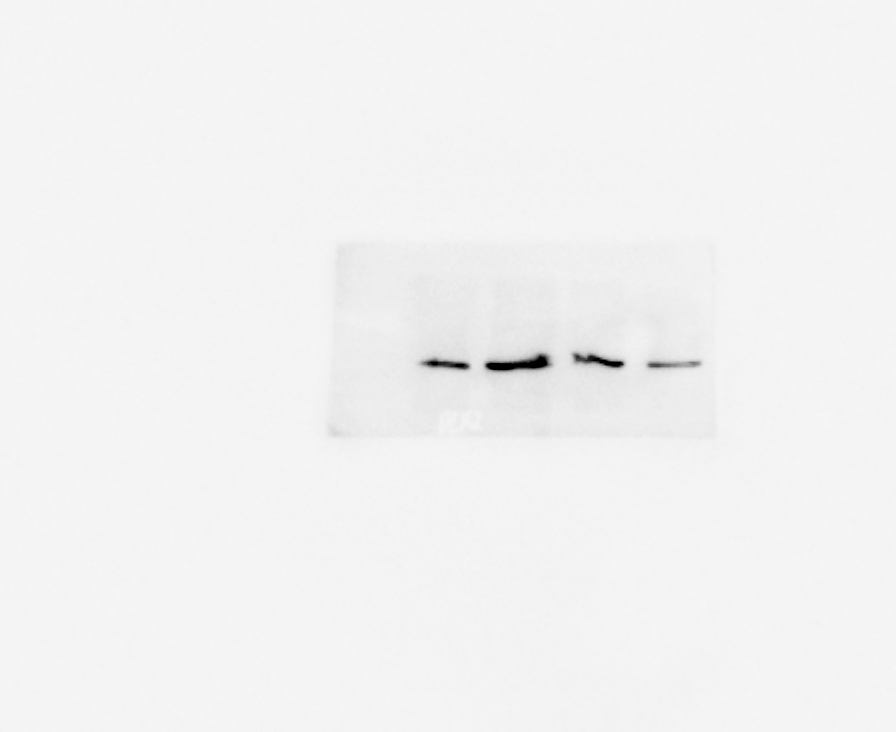


GAPDH


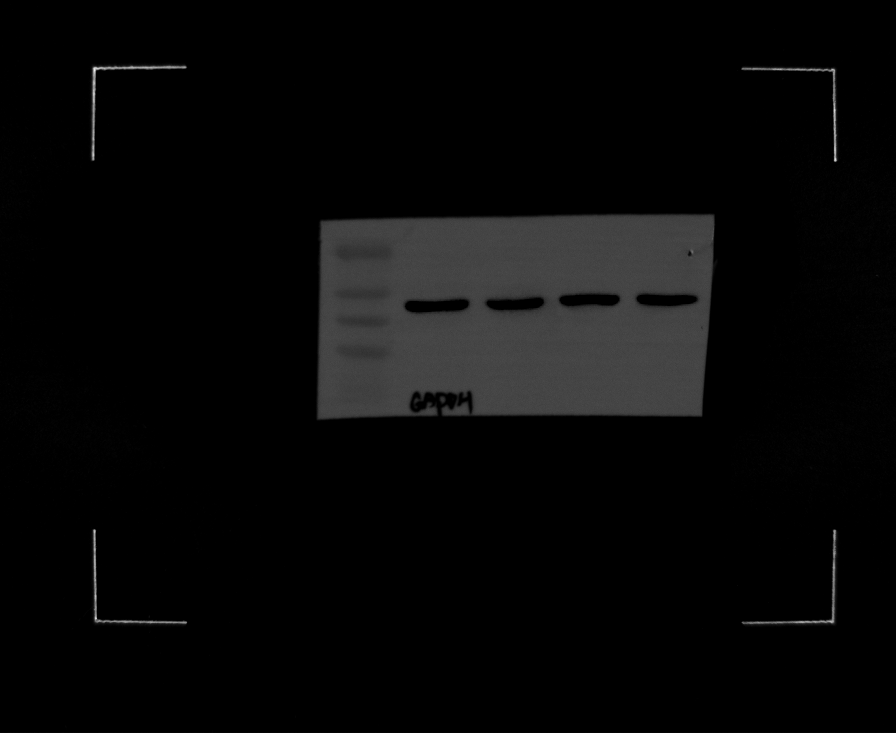

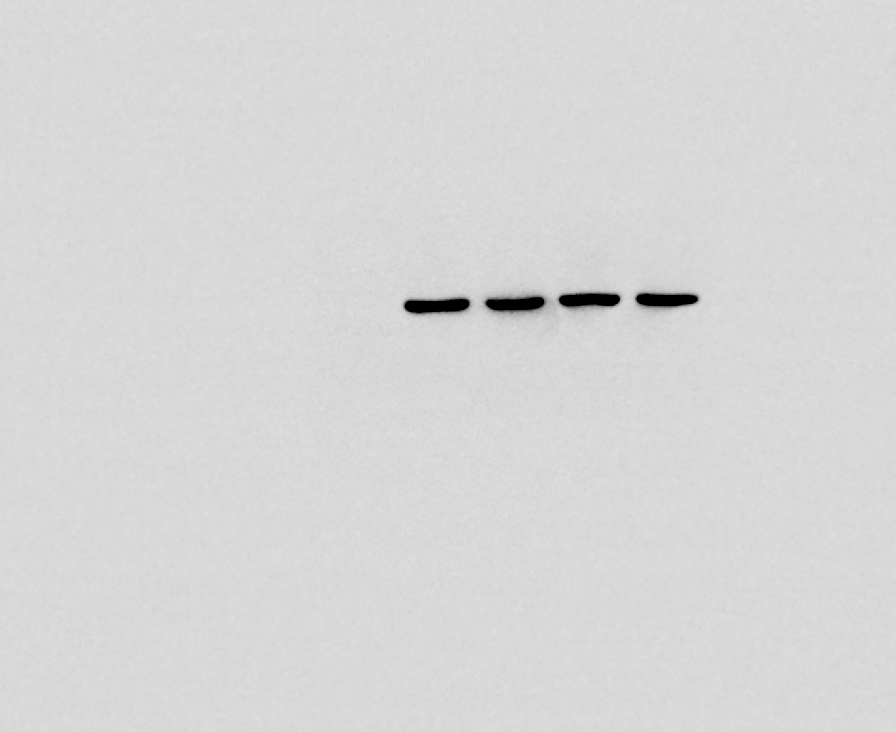


FIG2C

SENP3


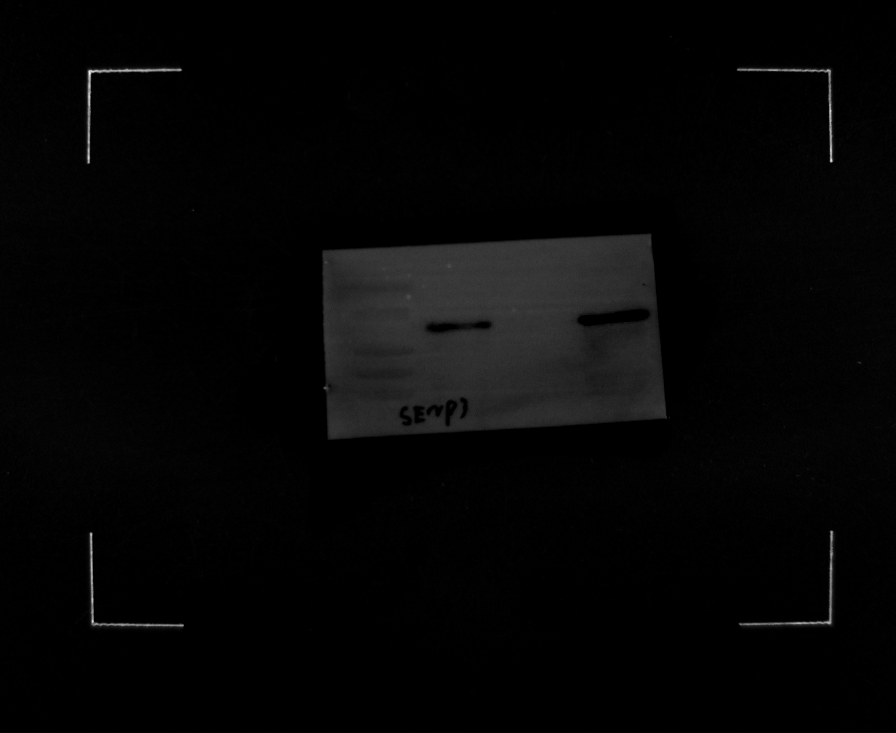

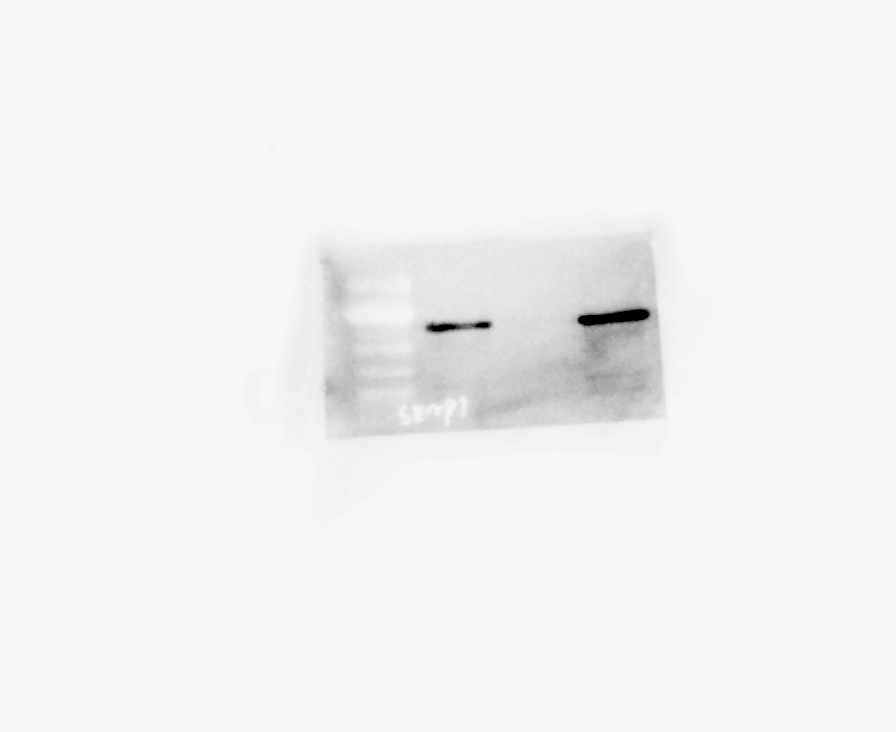


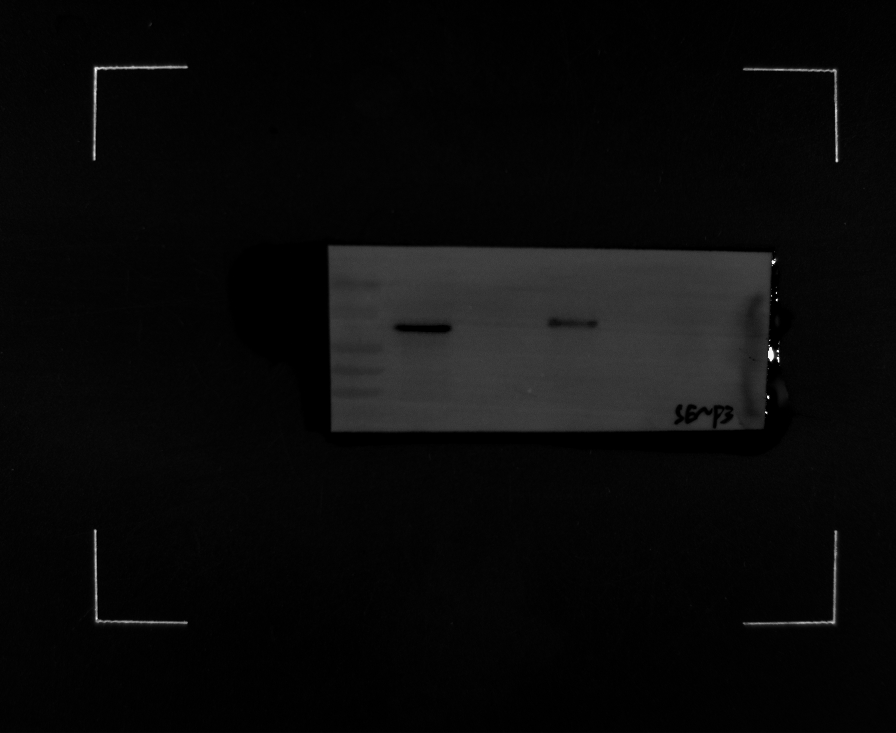

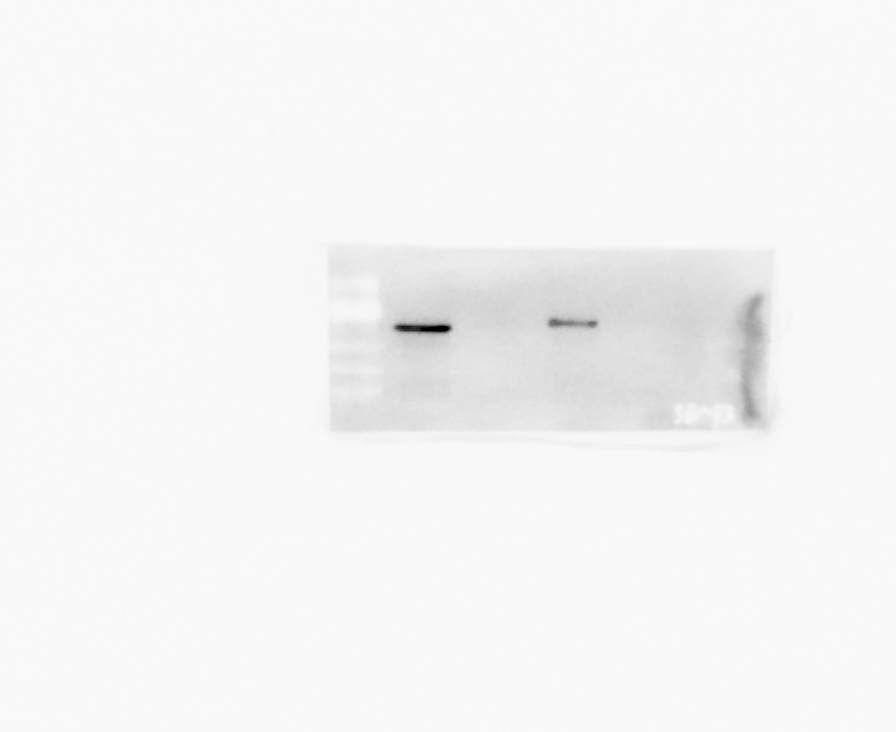


DLX2


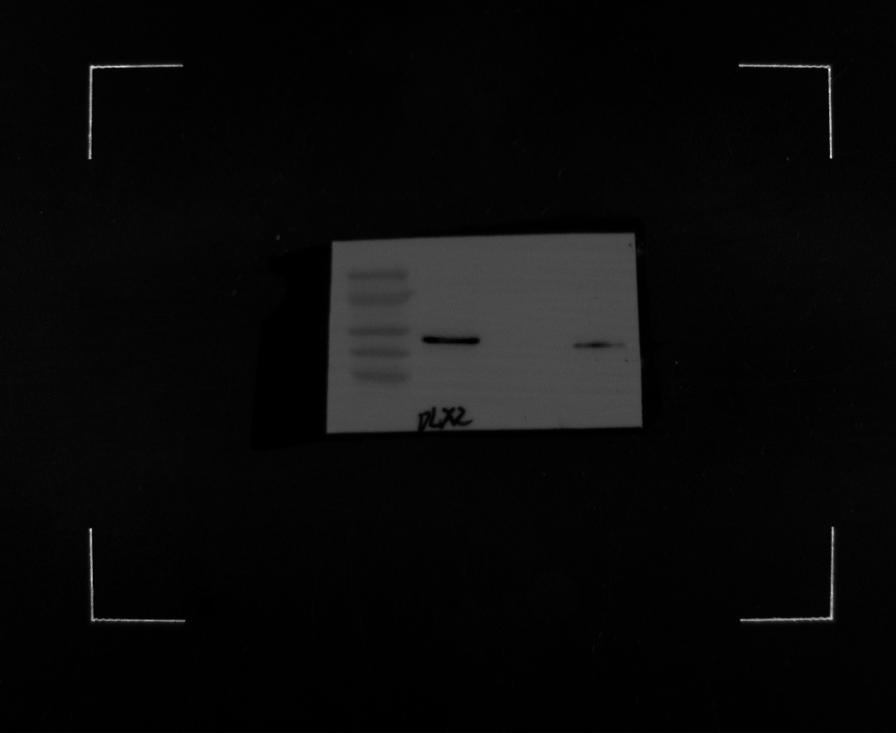

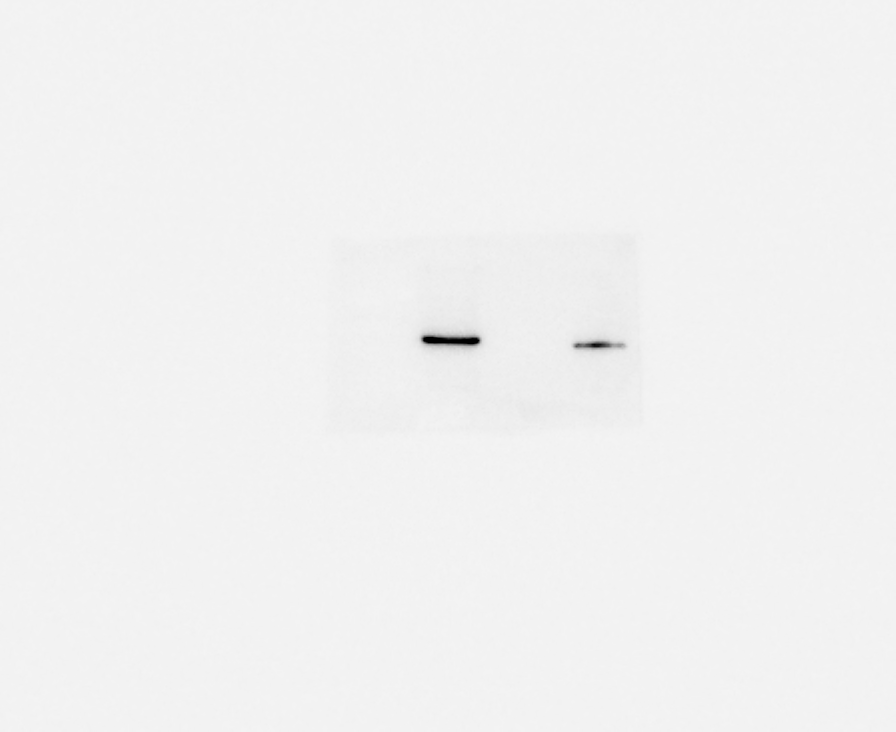


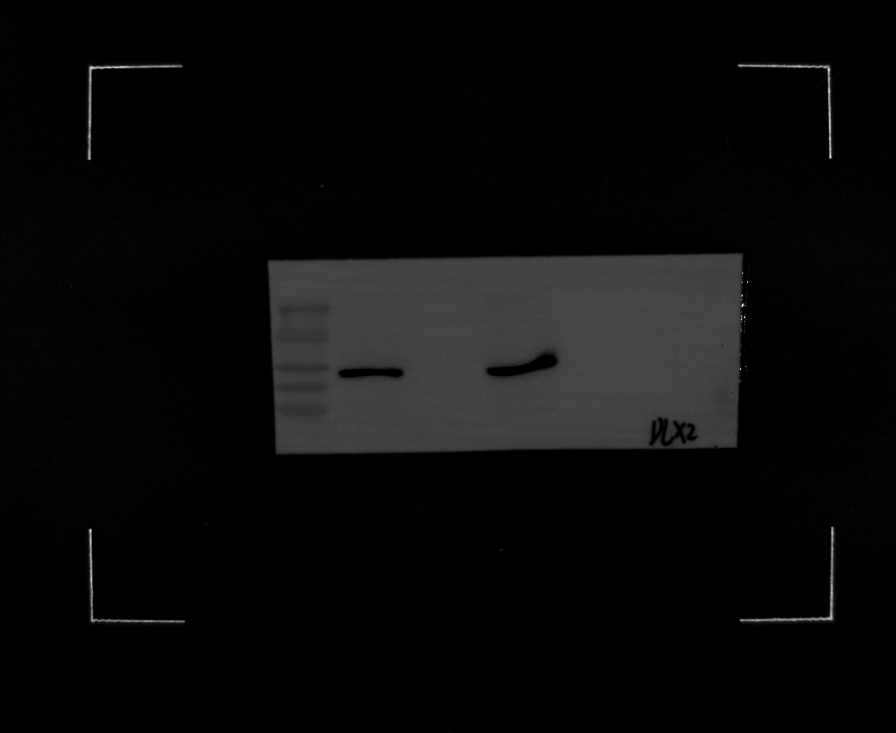

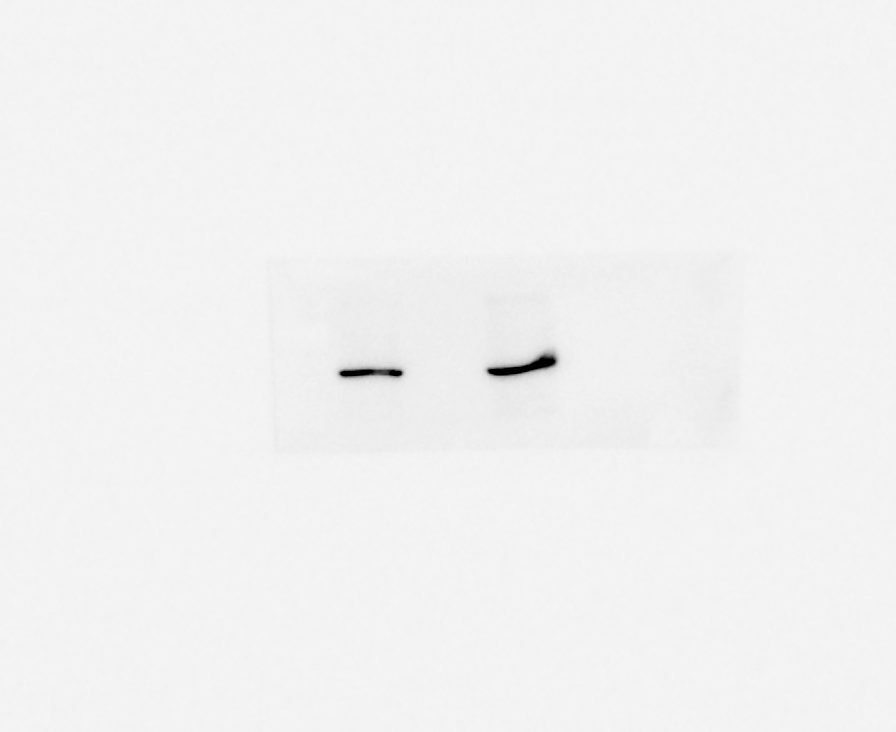


FIG2D

Input

DLX2


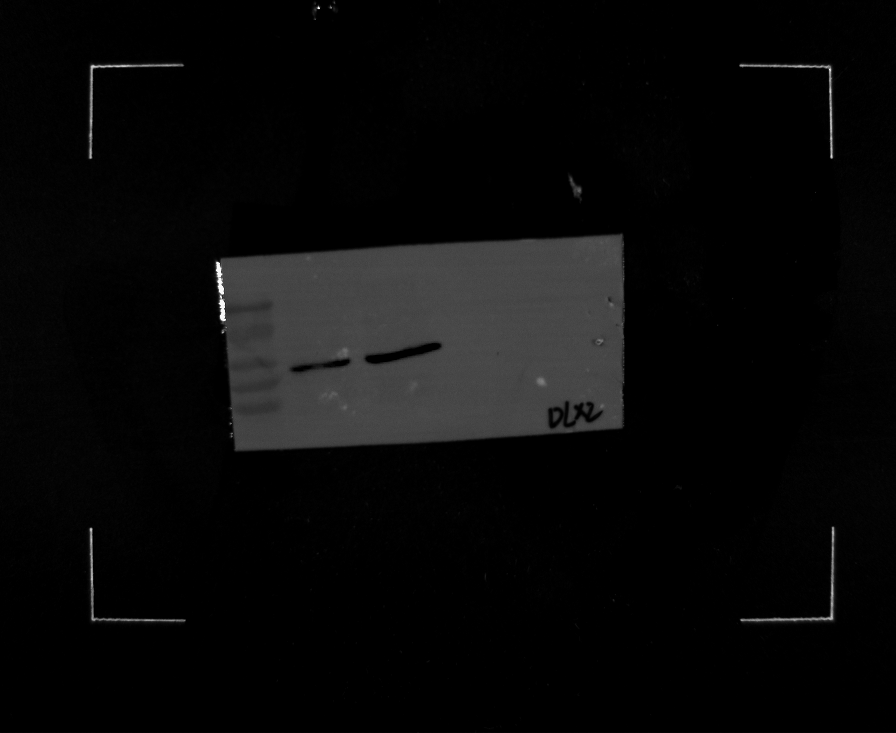

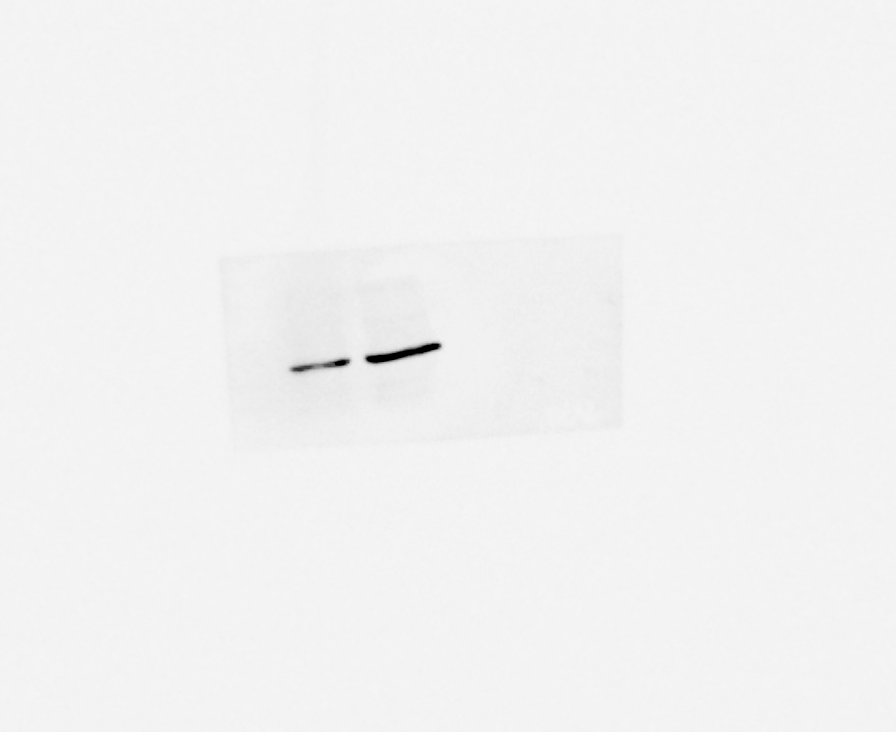


SENP3


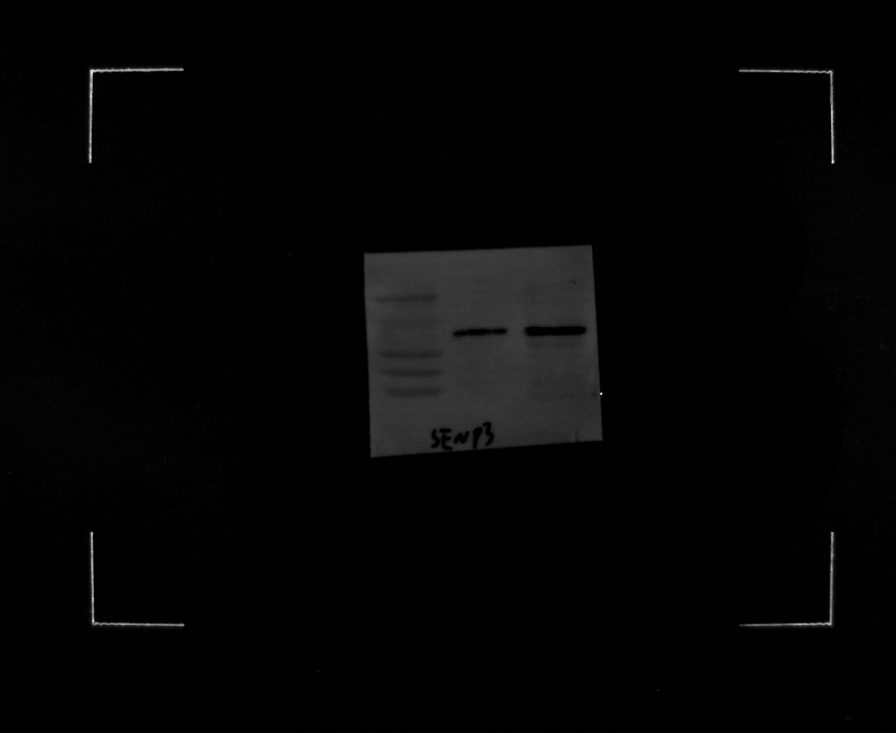

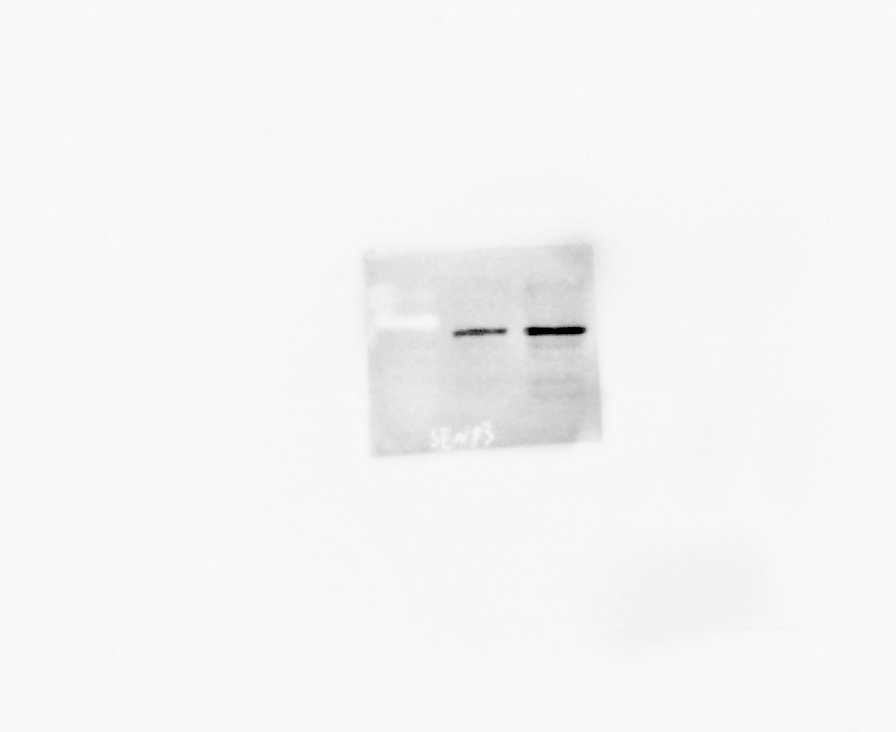


GAPDH


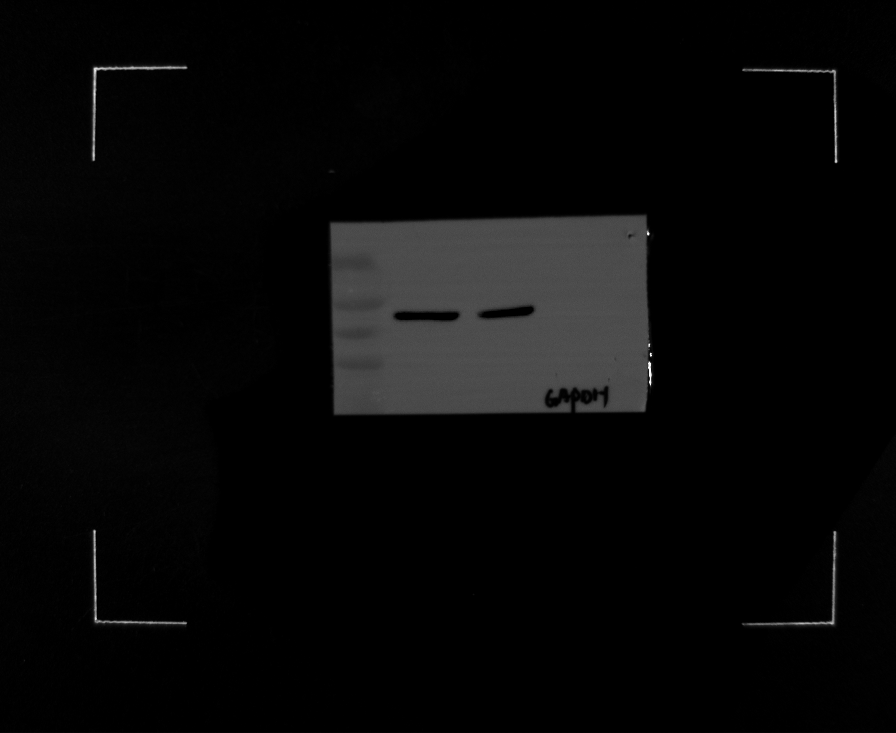

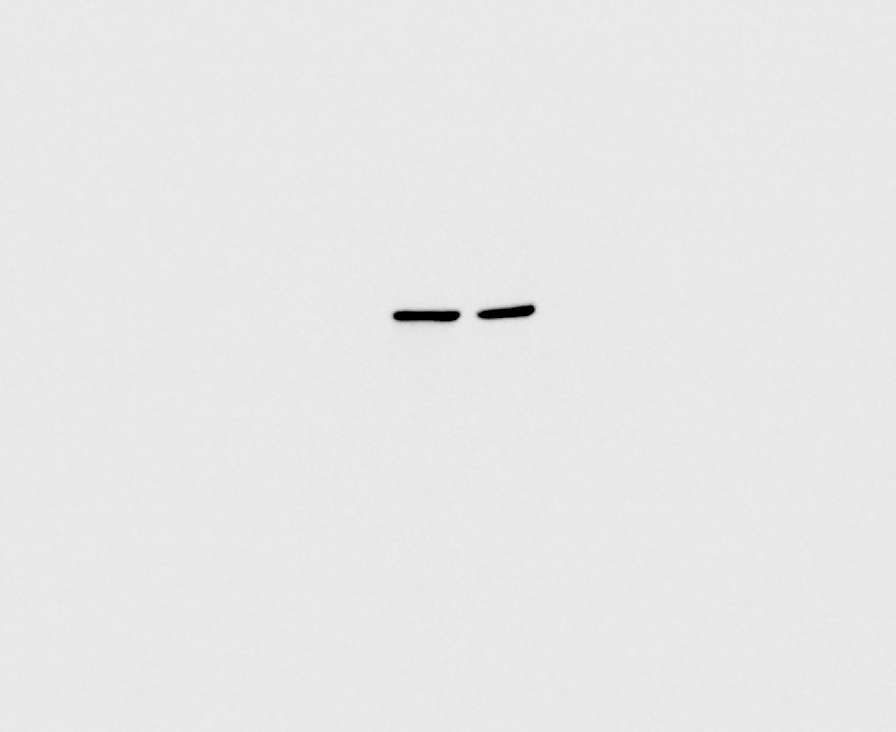


IP

DLX2-SUMO2/3-sumo2/3


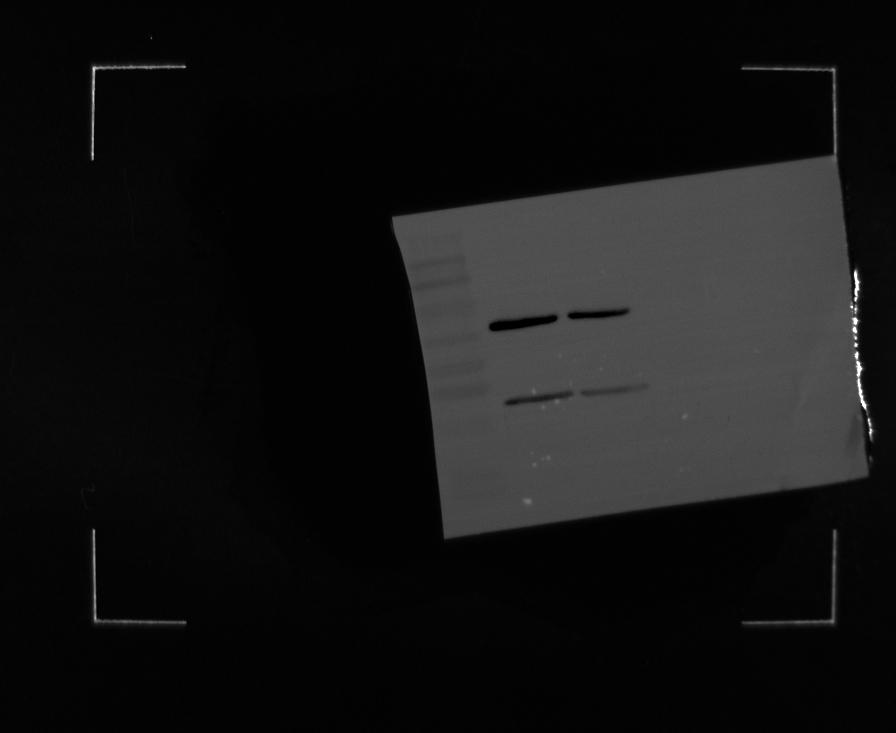

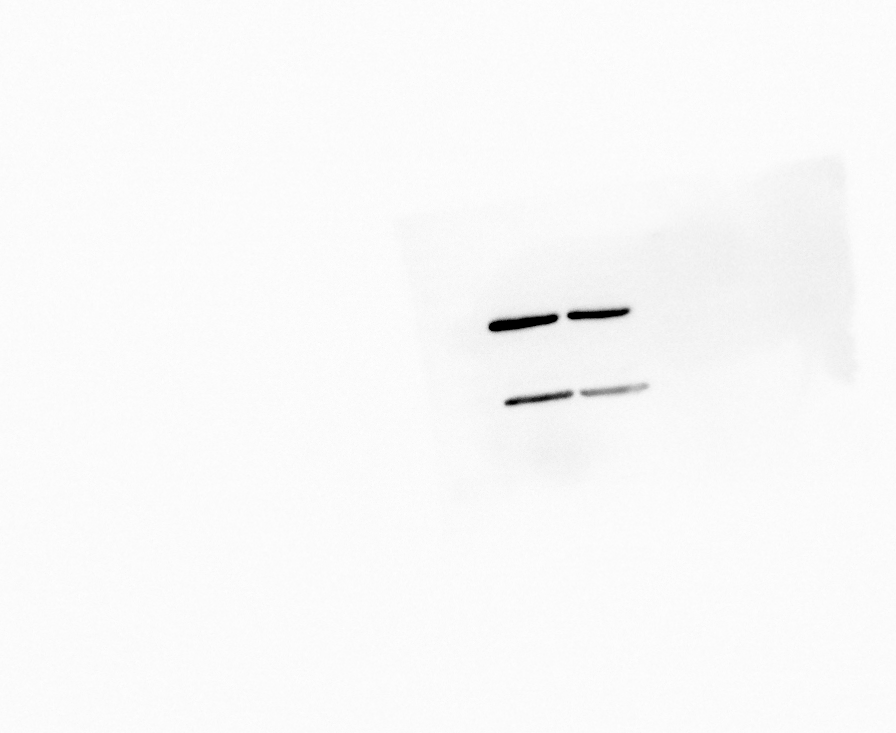


DLX2-SUMO2/3-DLX2


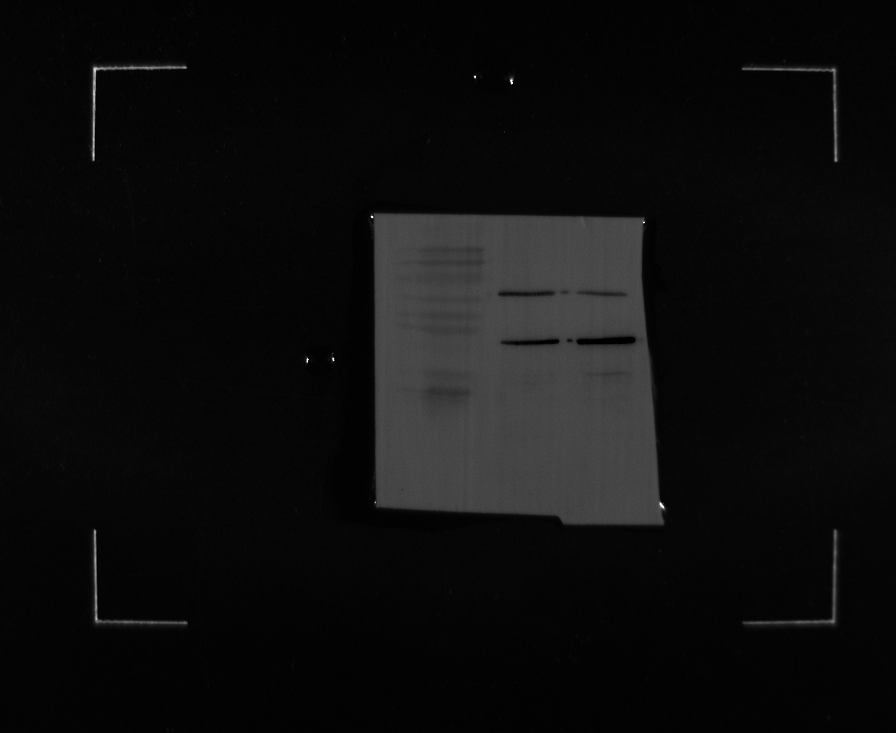

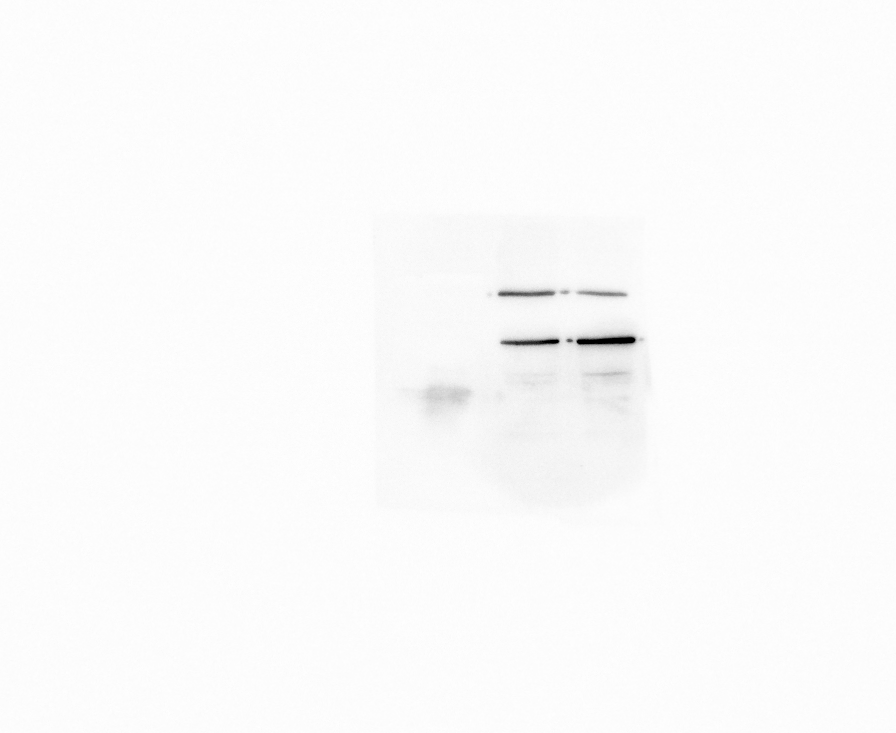


FIG3B

SENP3


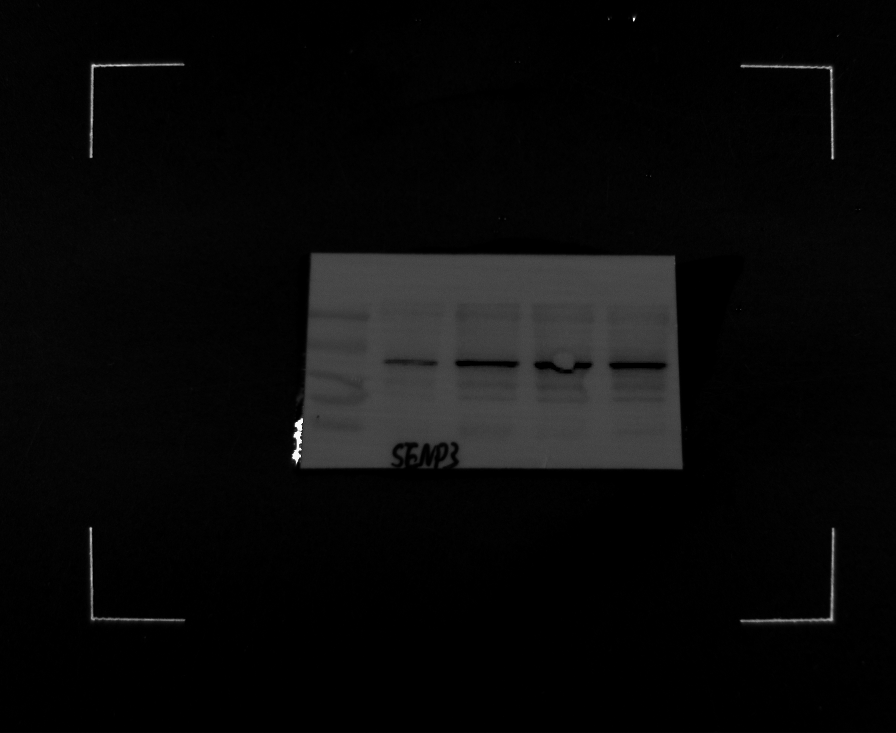

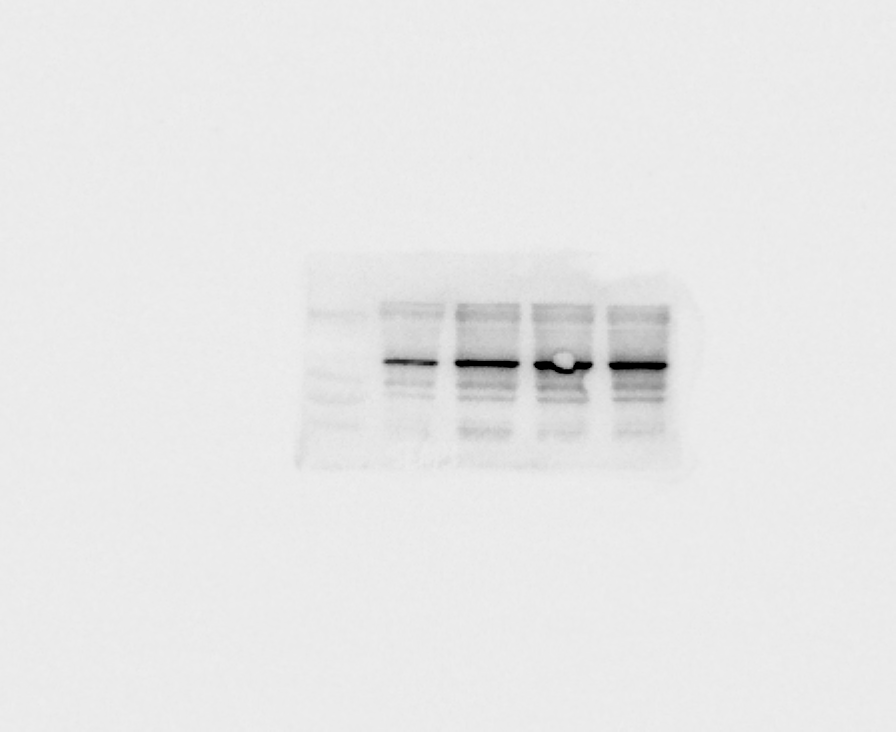


DLX2


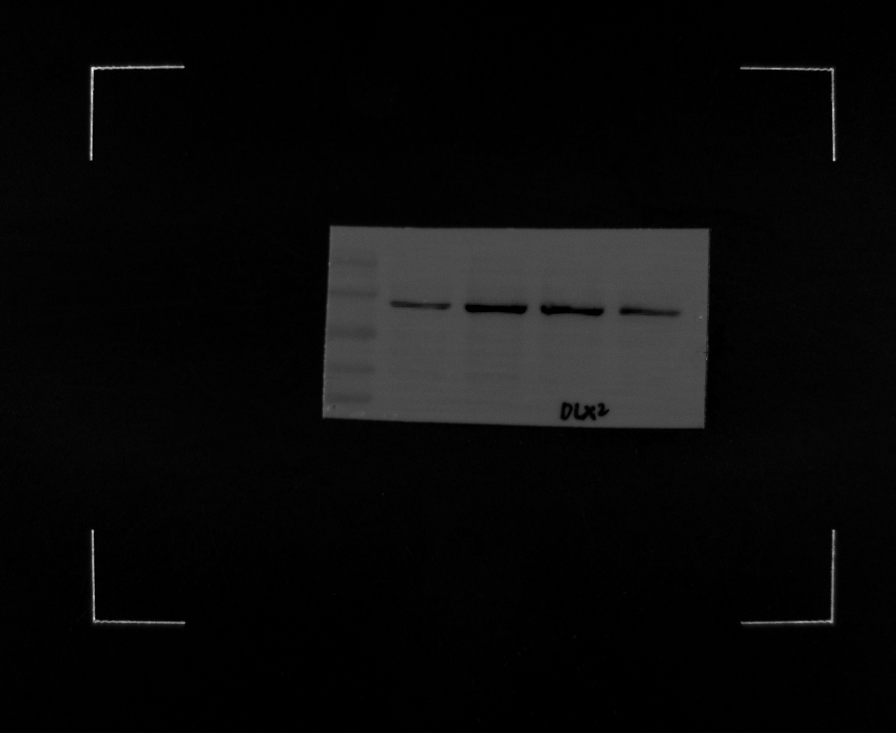

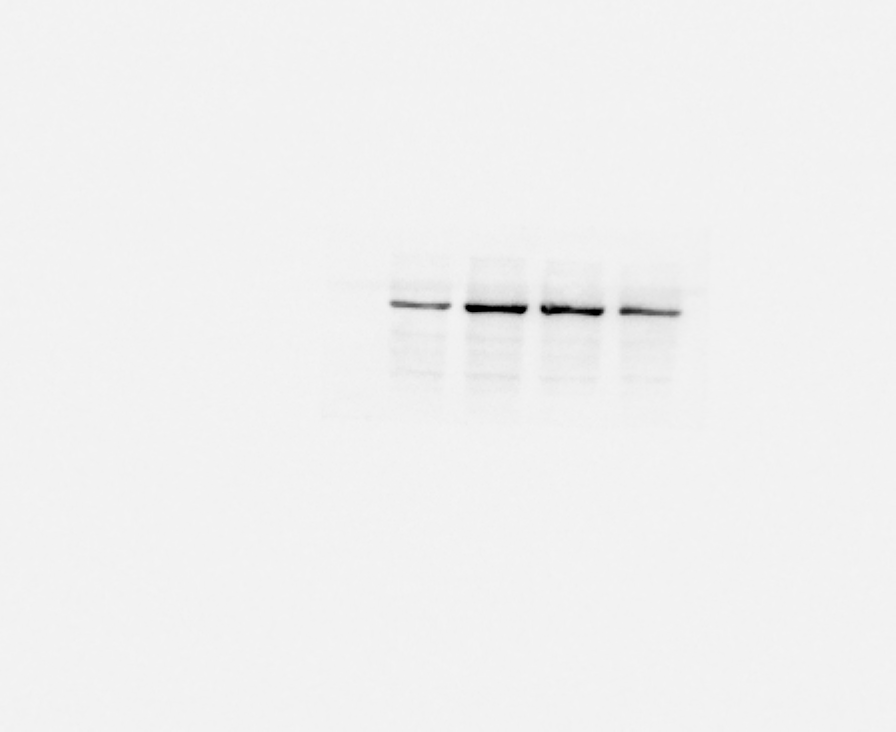


GAPDH


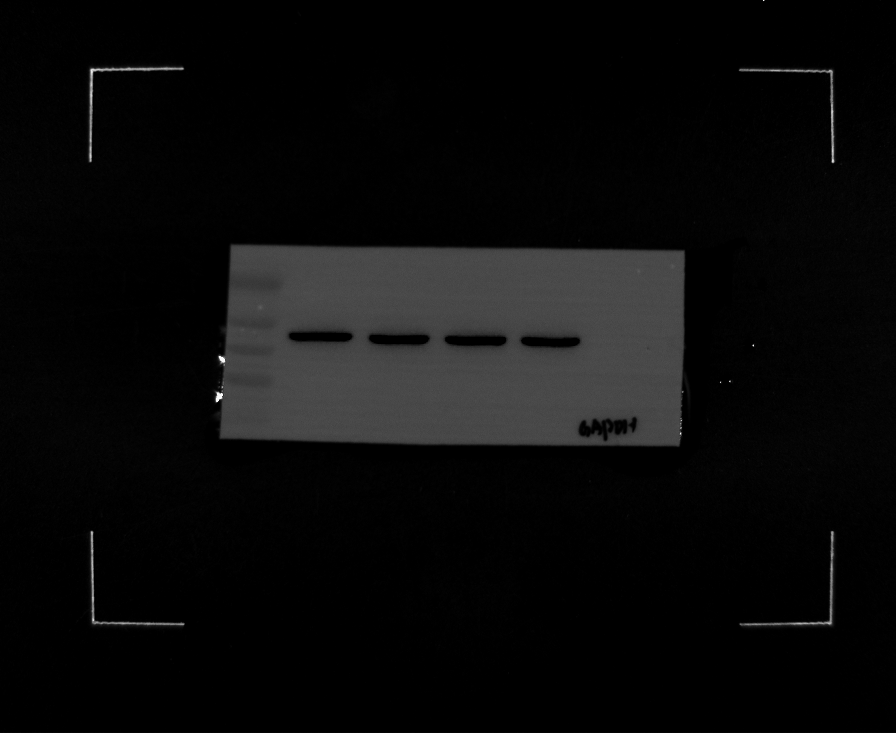

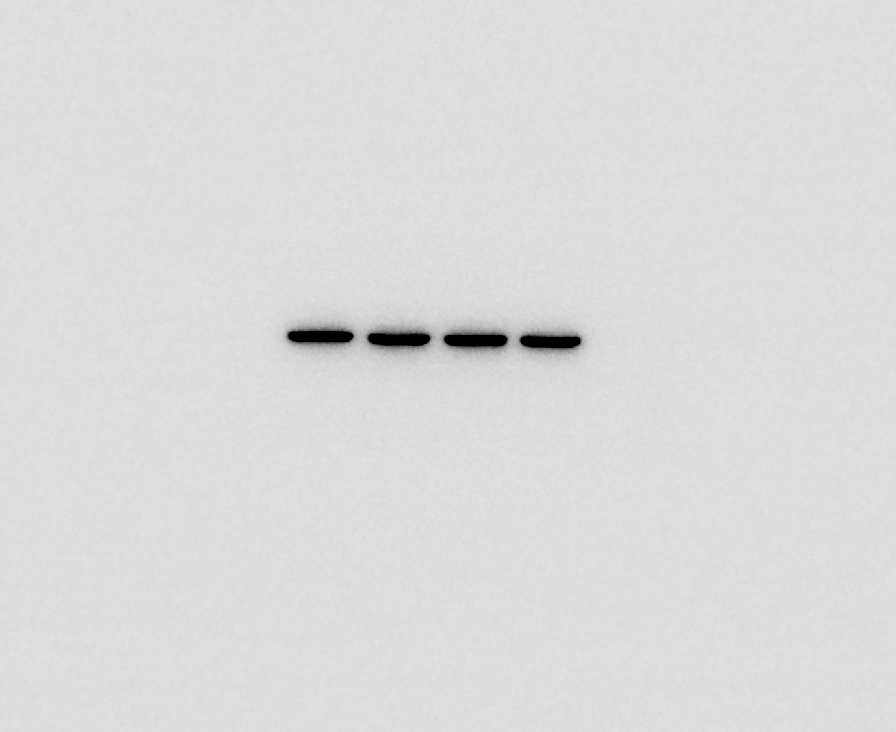


FIG 3G

OPN


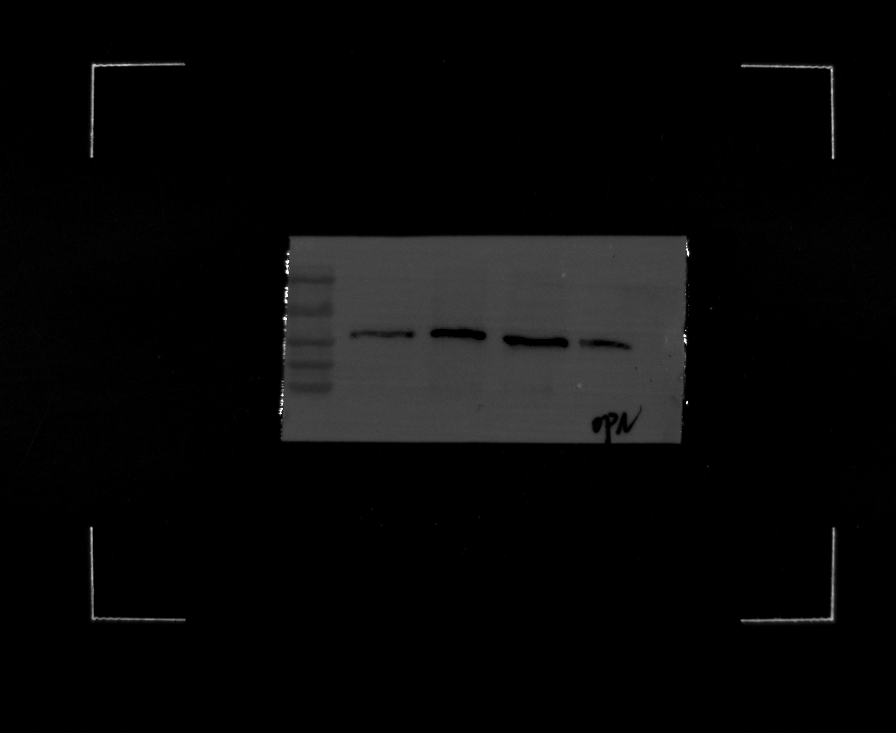

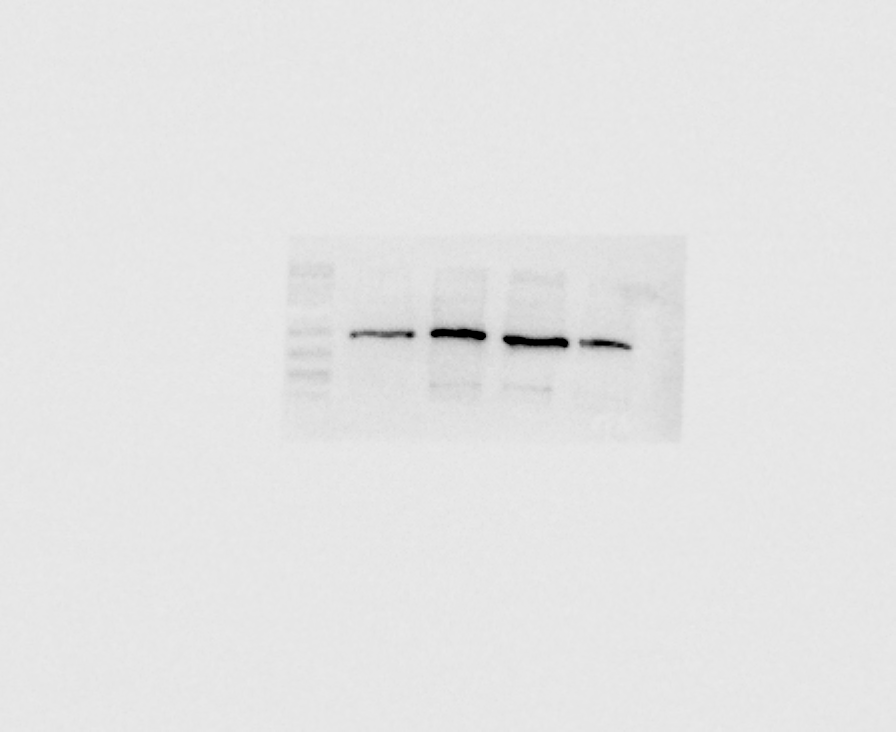


OCN


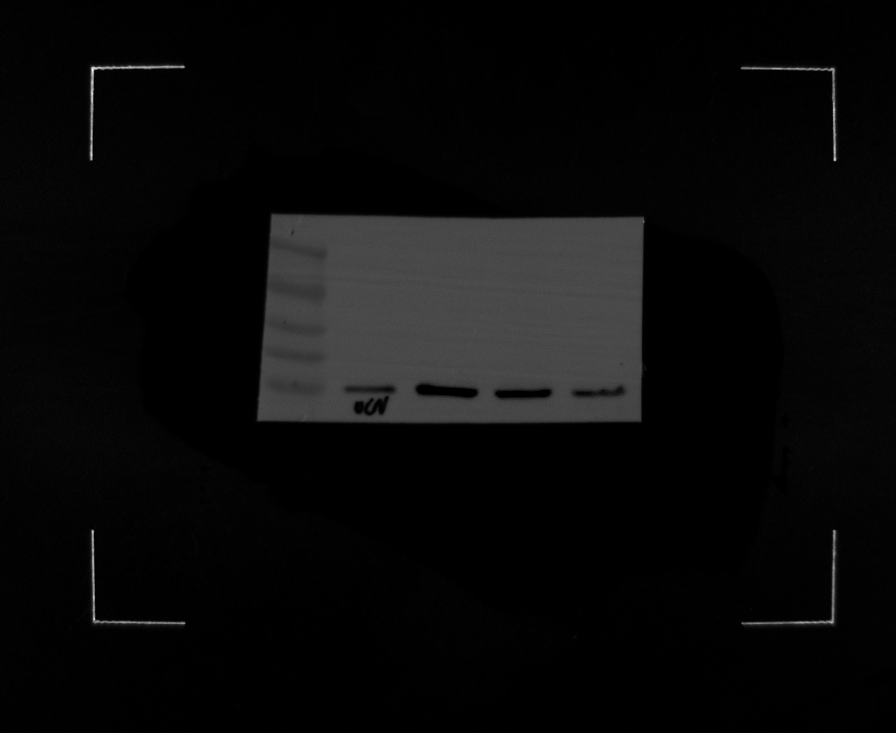

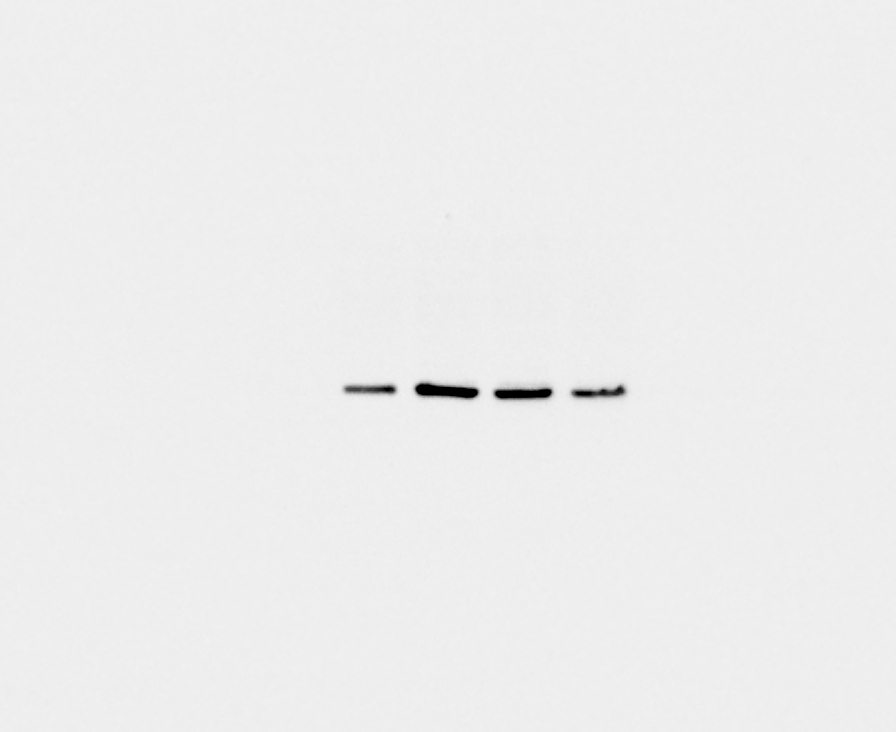


RUNX2


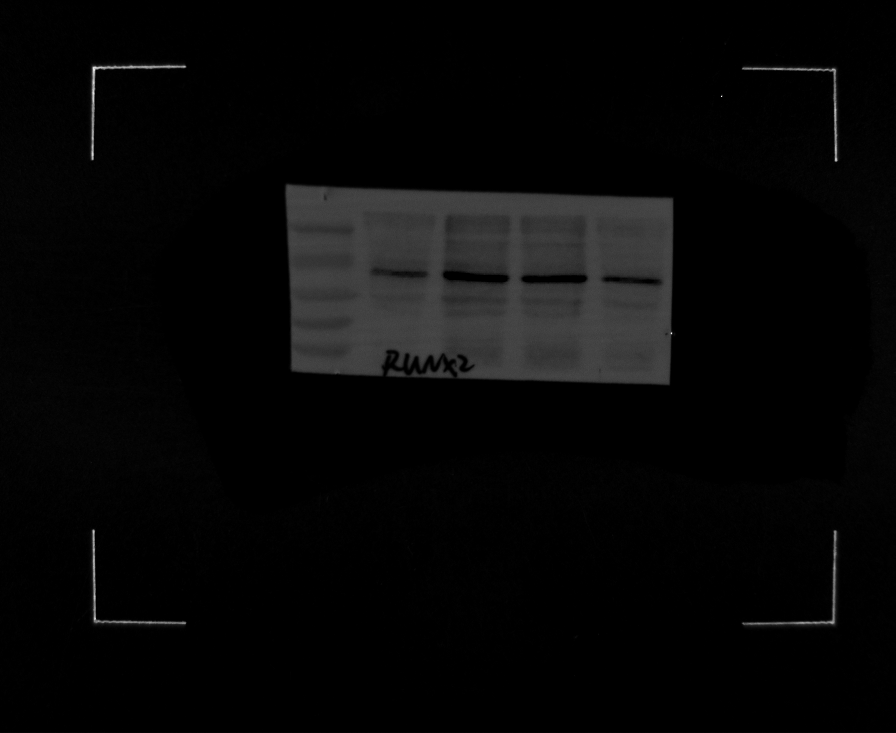

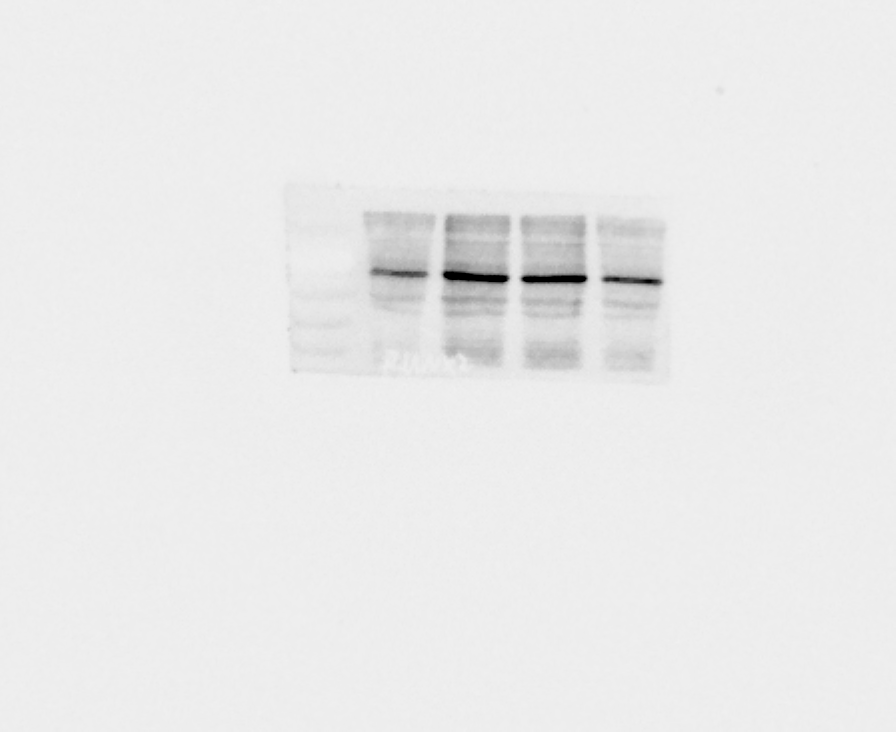


GAPDH


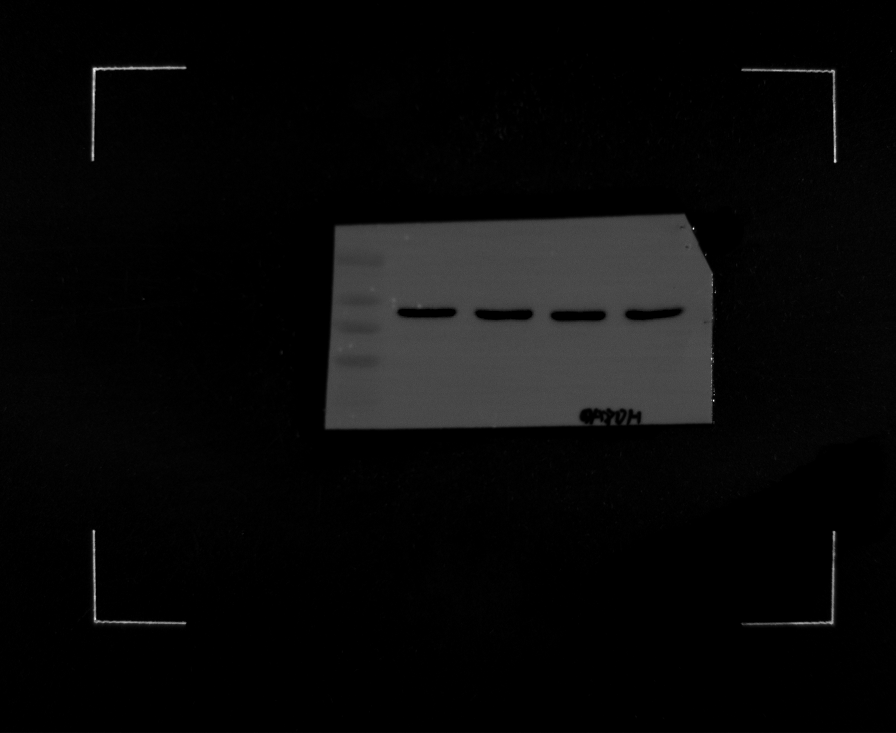

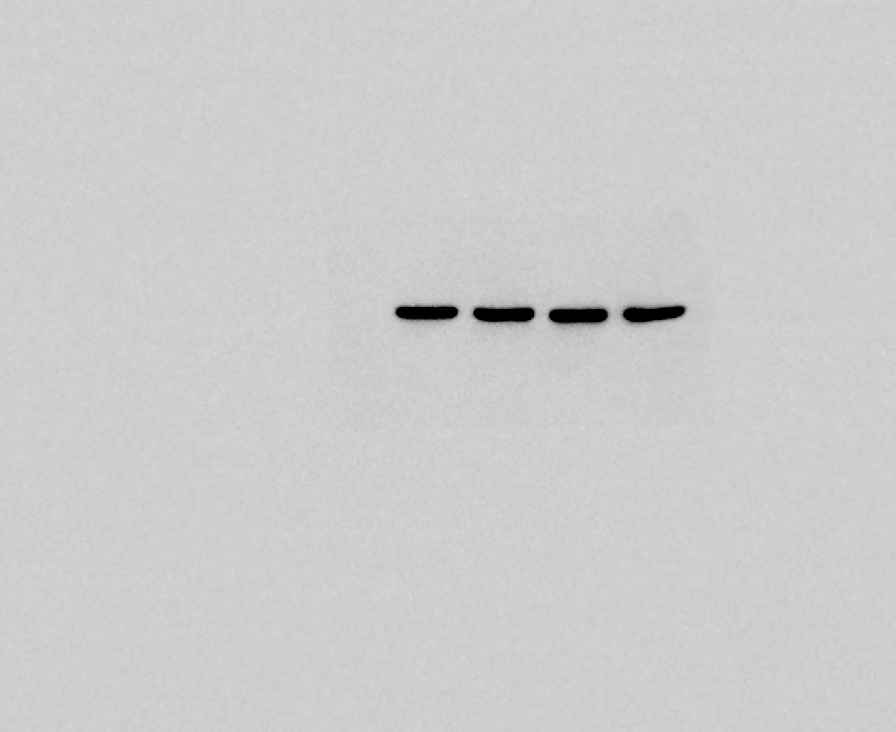


FIG 4E

DLX2


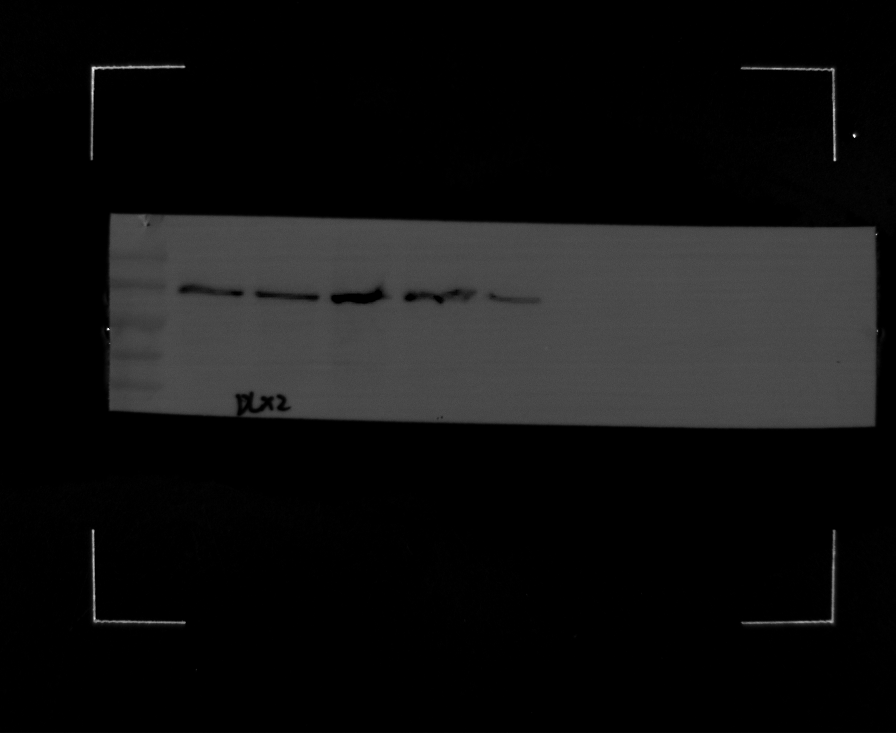

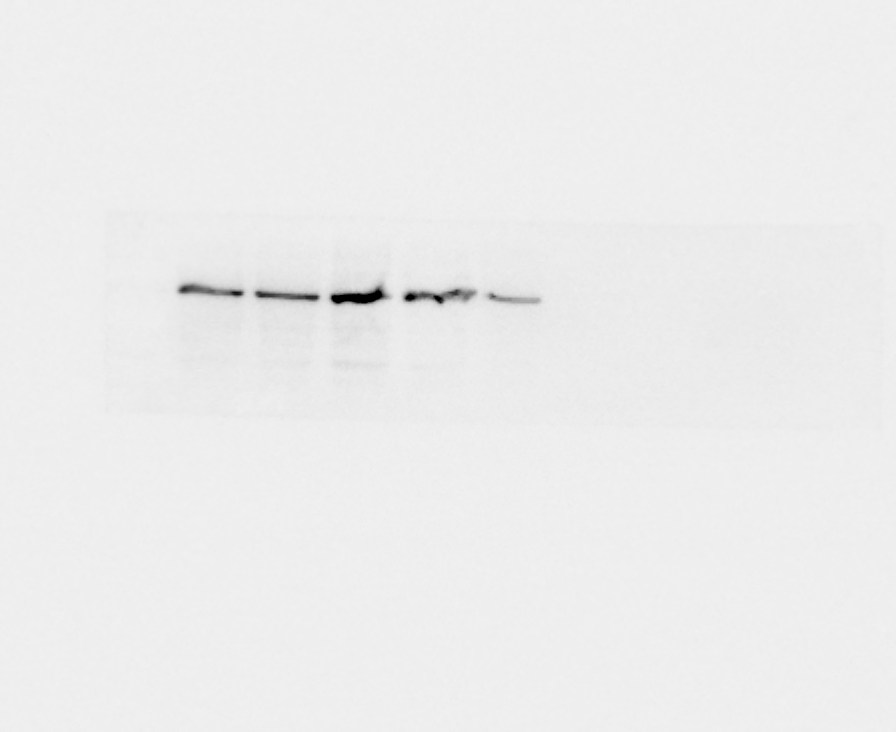


SIRT3


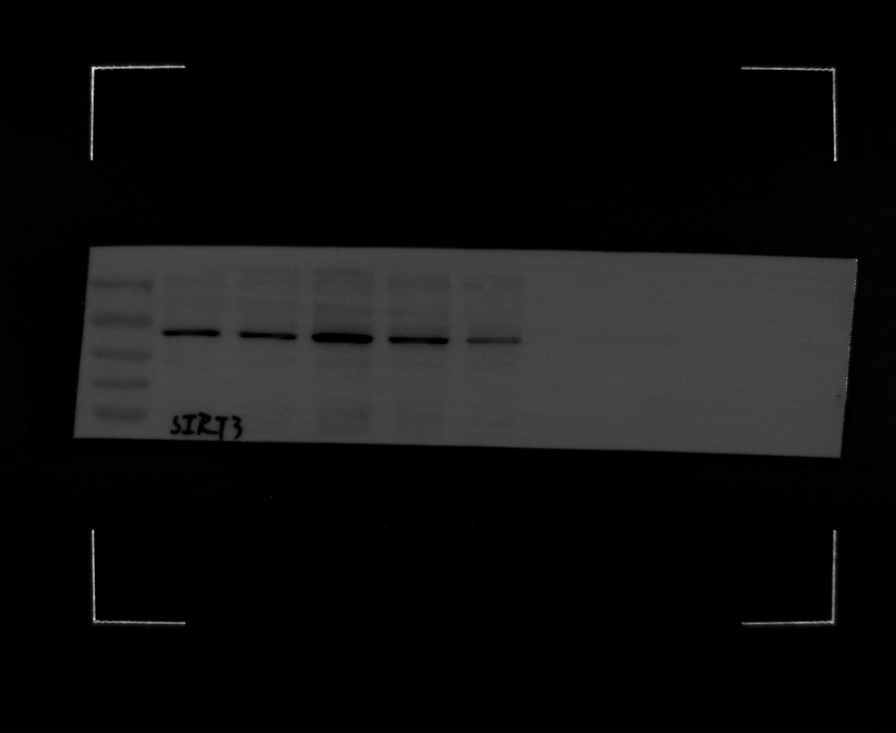

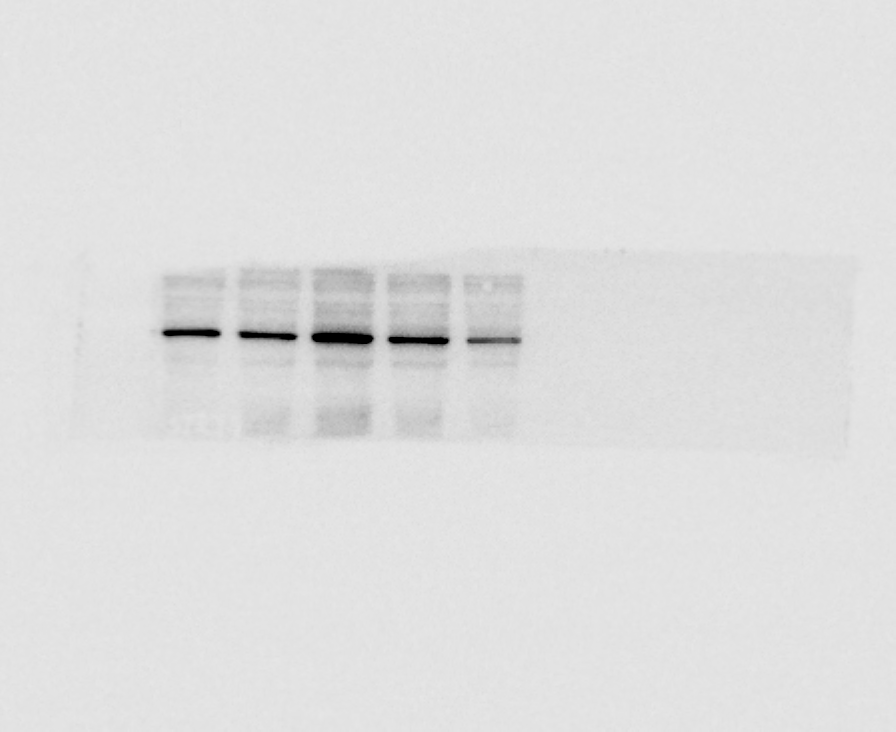


GAPDH


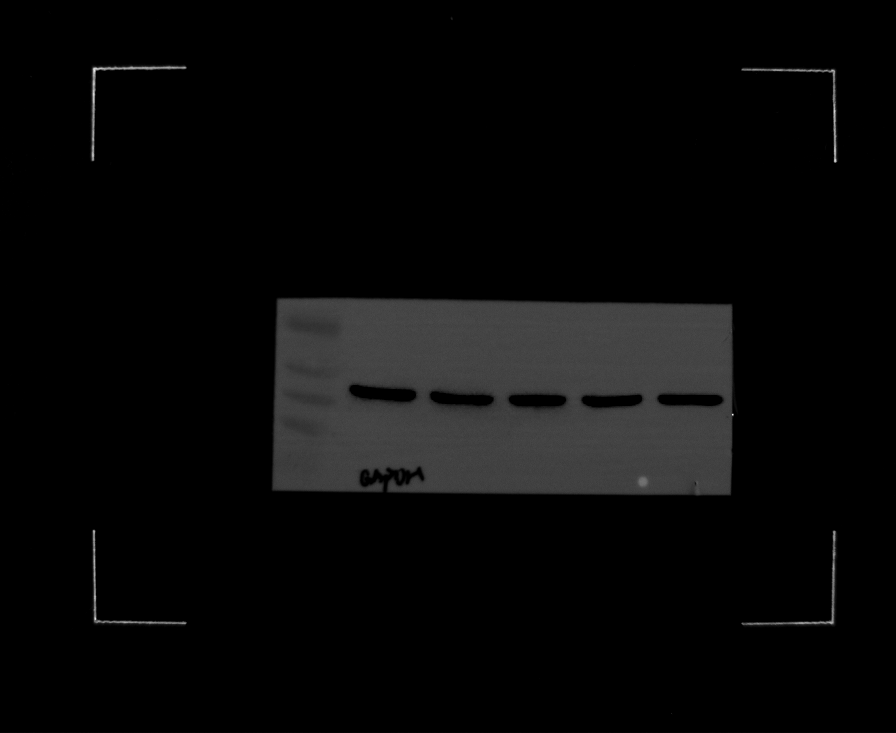

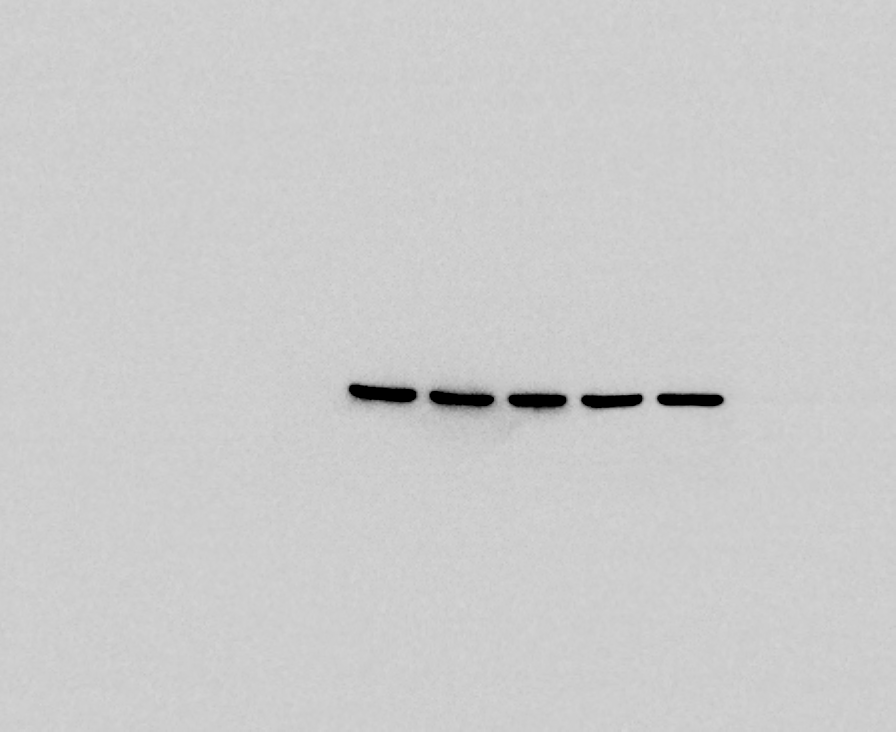


FIG 5B

DLX2


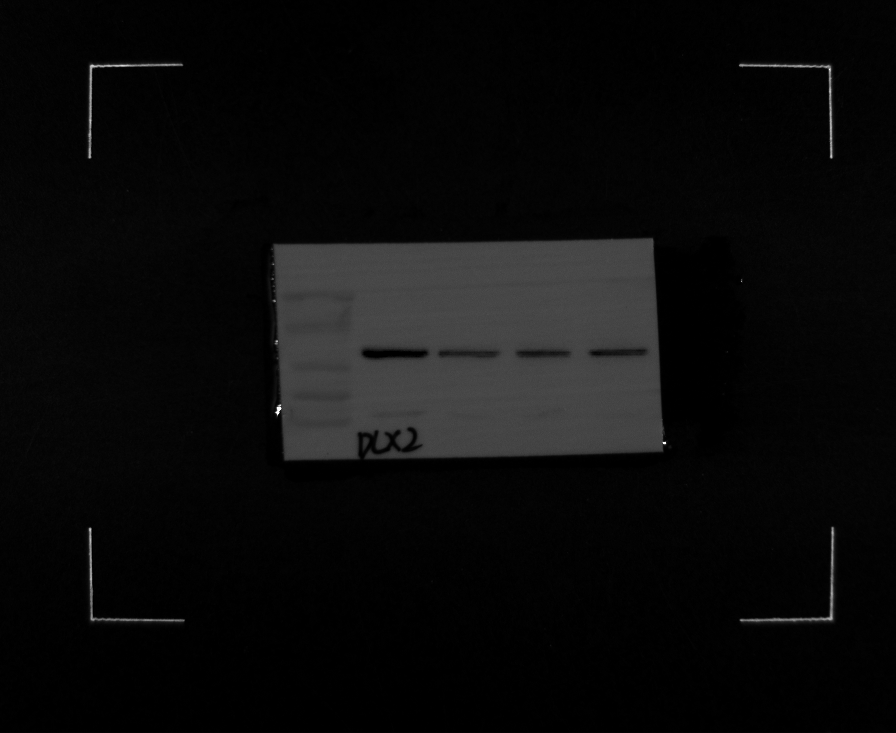

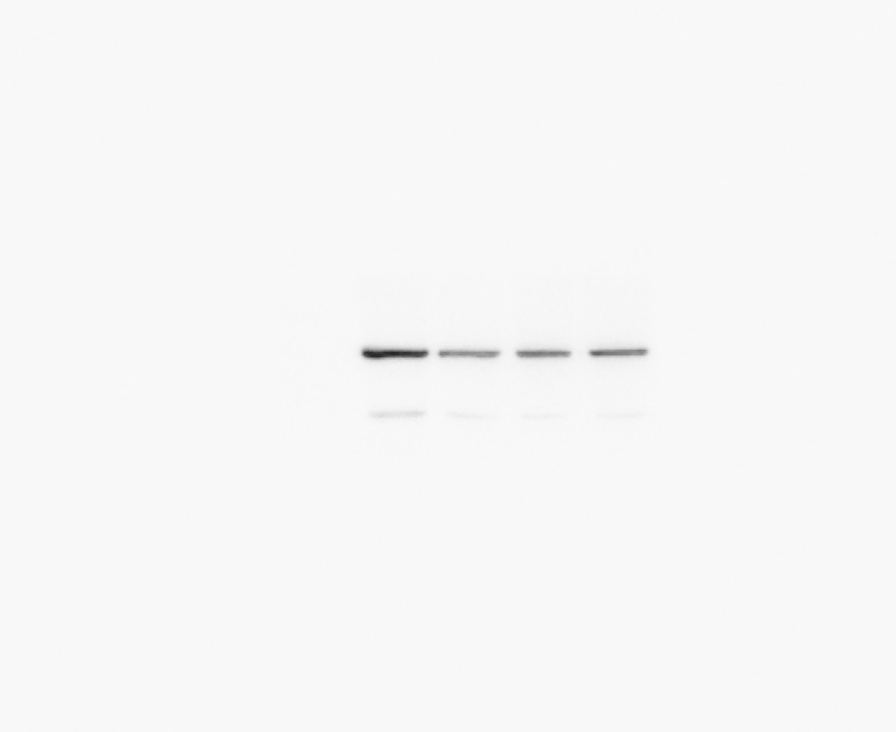


SIRT3


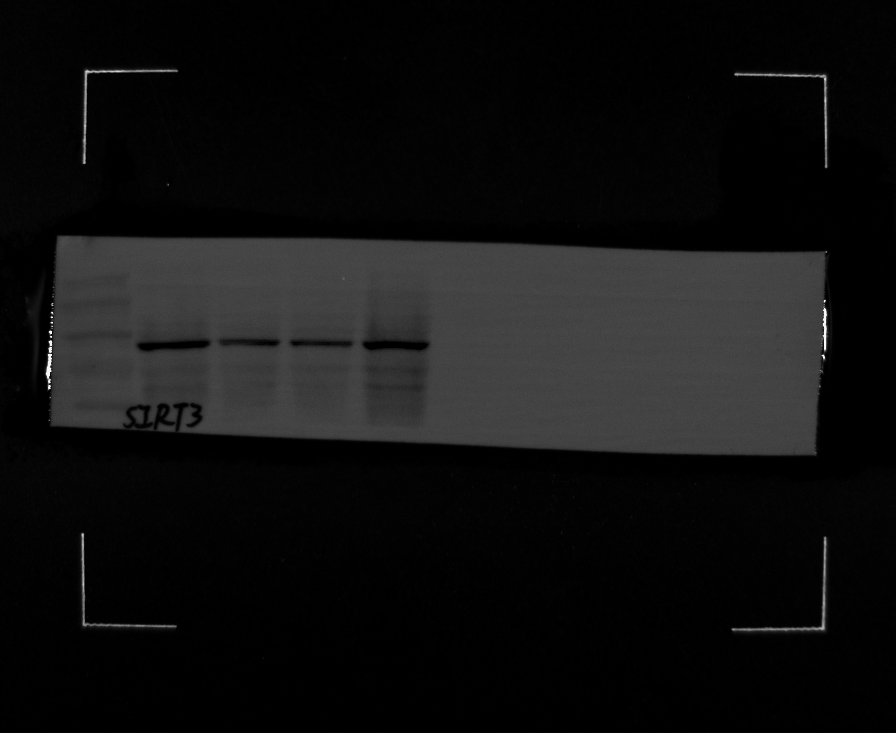

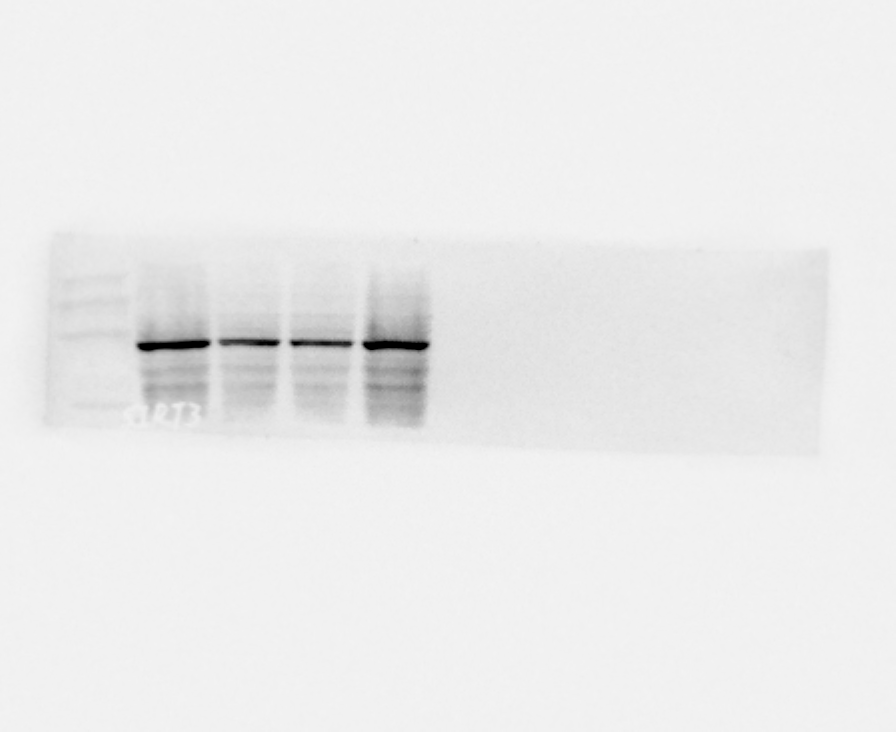


GAPDH


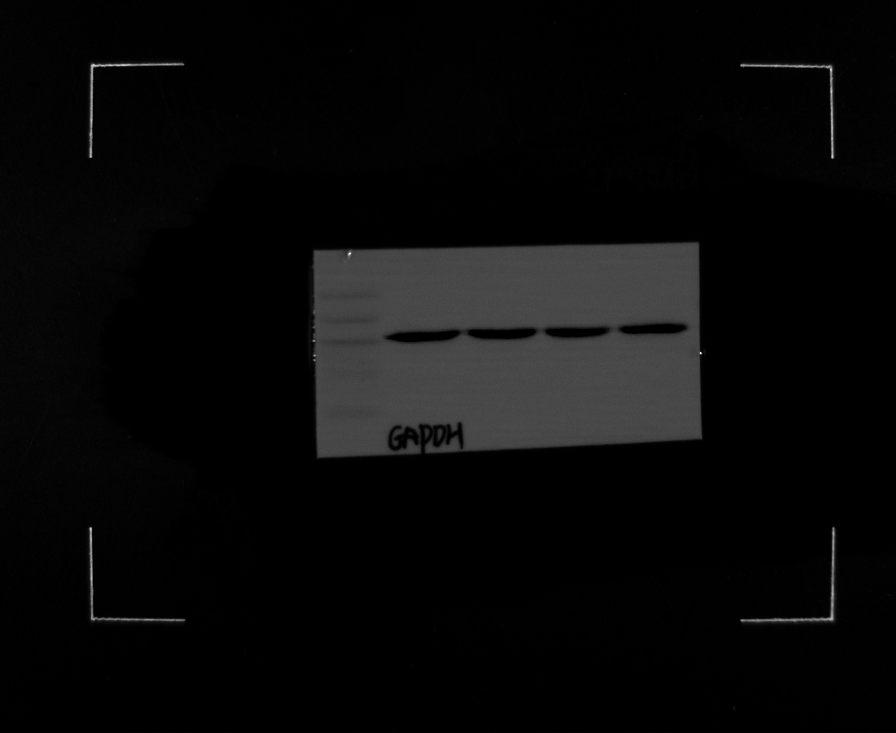

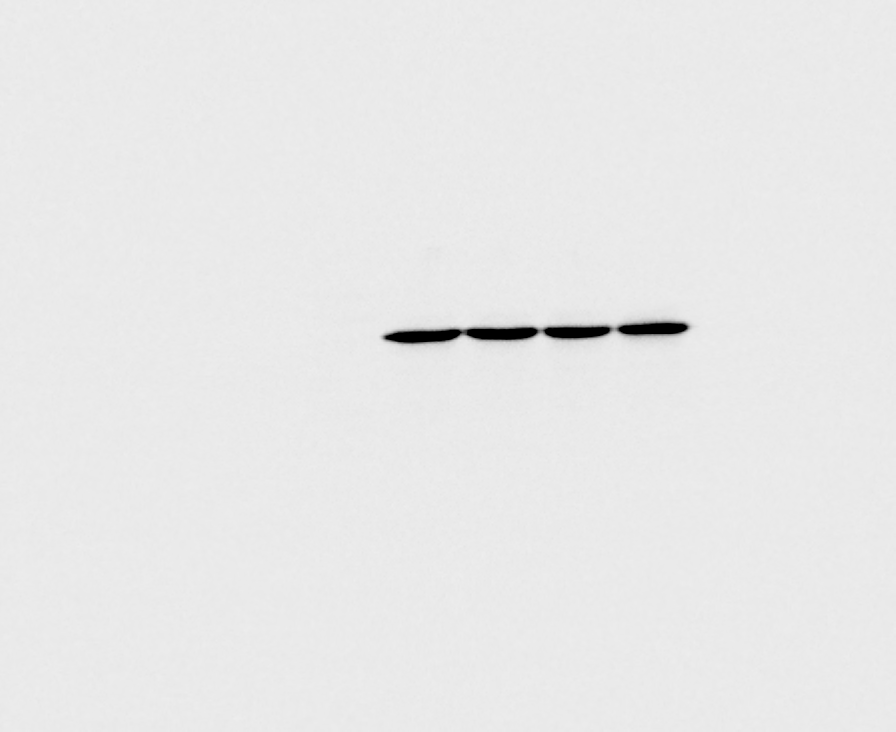


FIG 5G

OPN


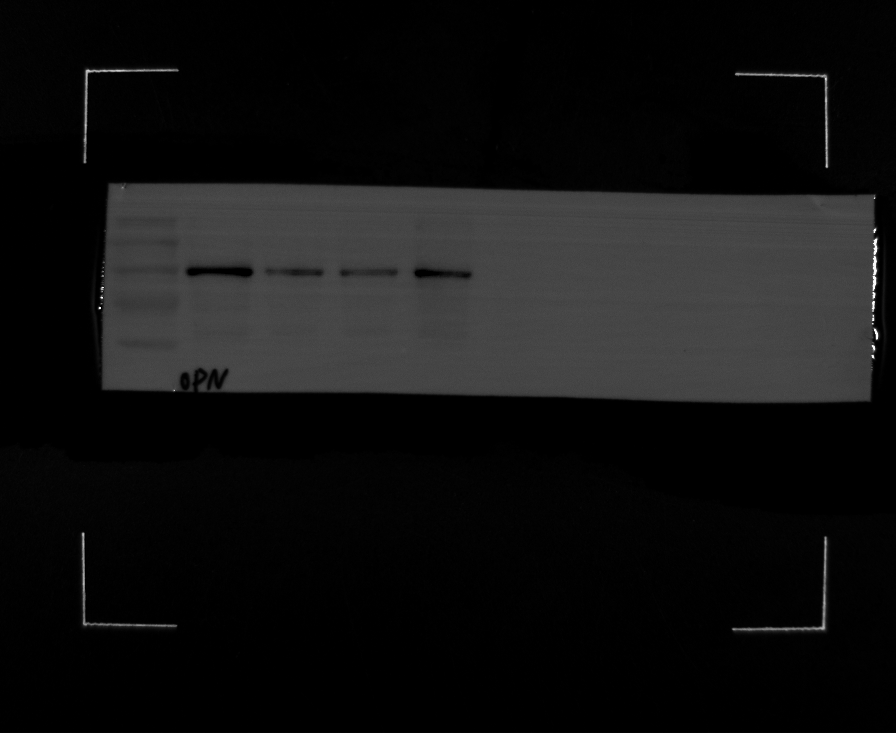

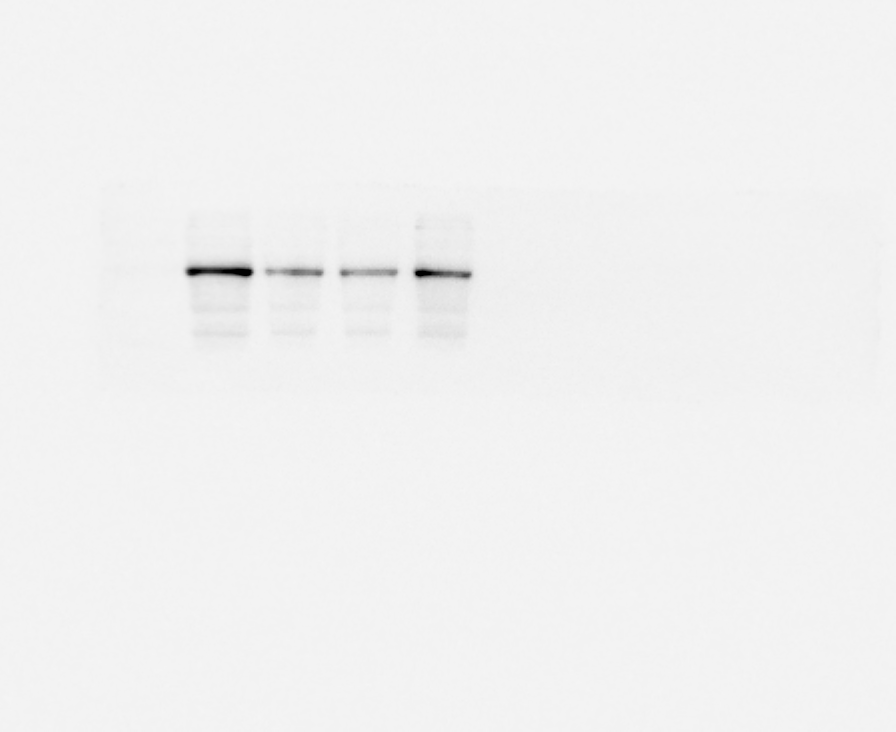


OCN


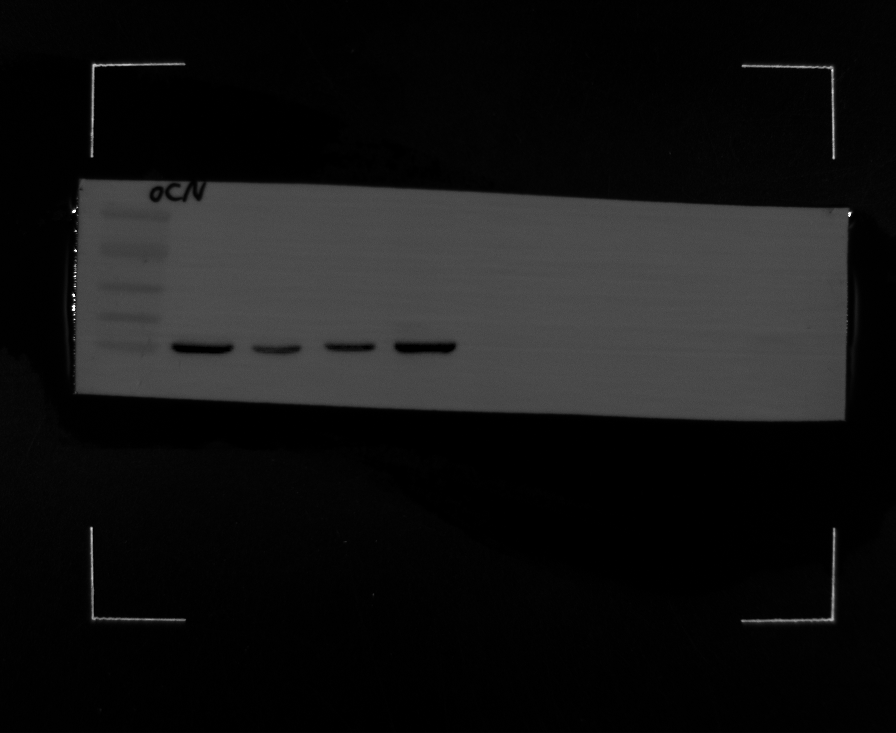

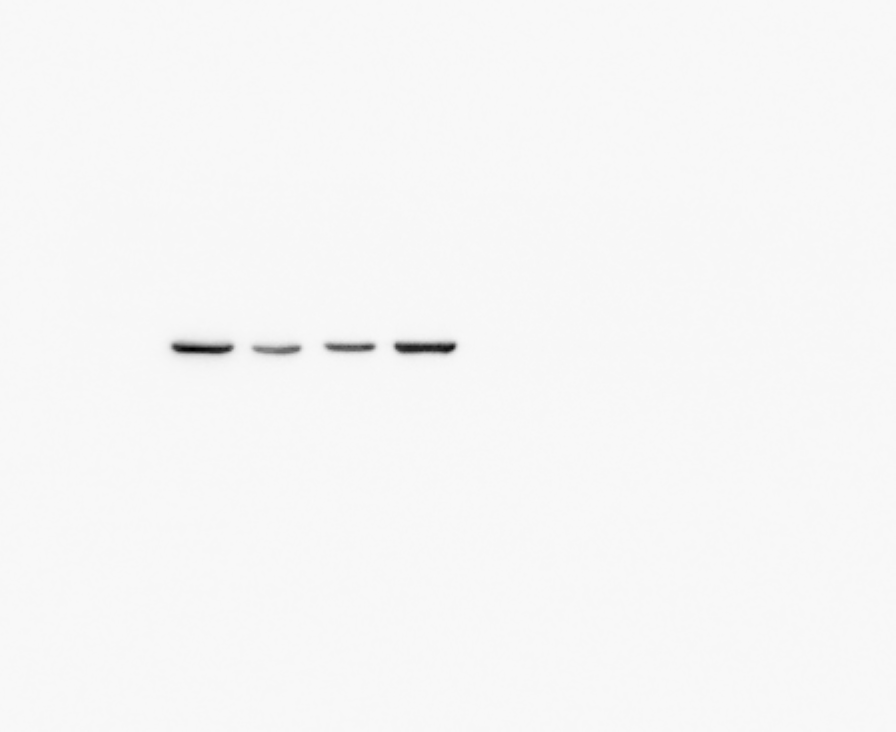


RUNX2


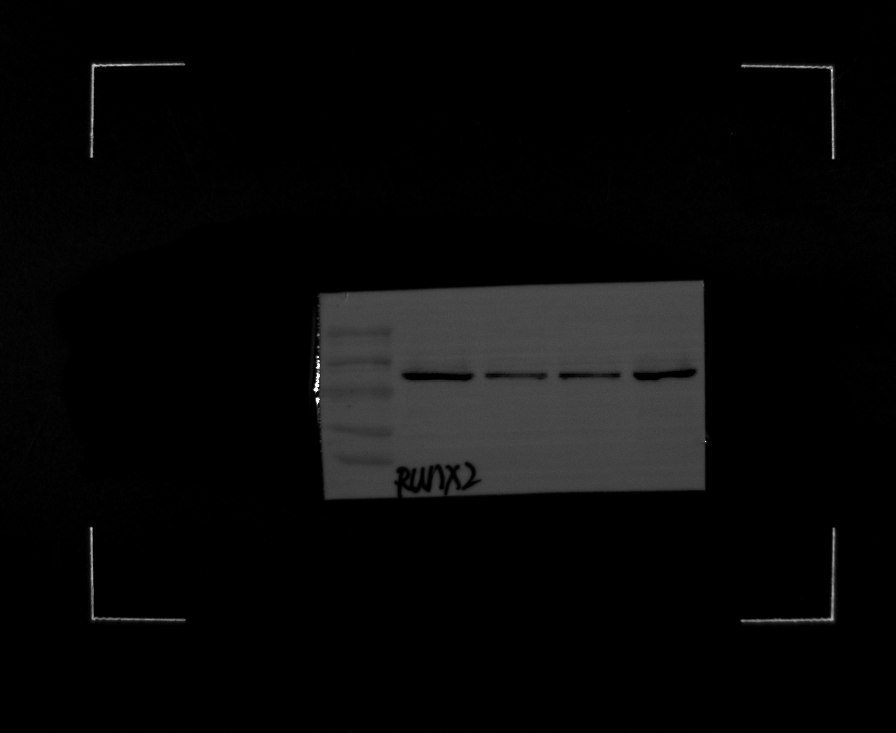

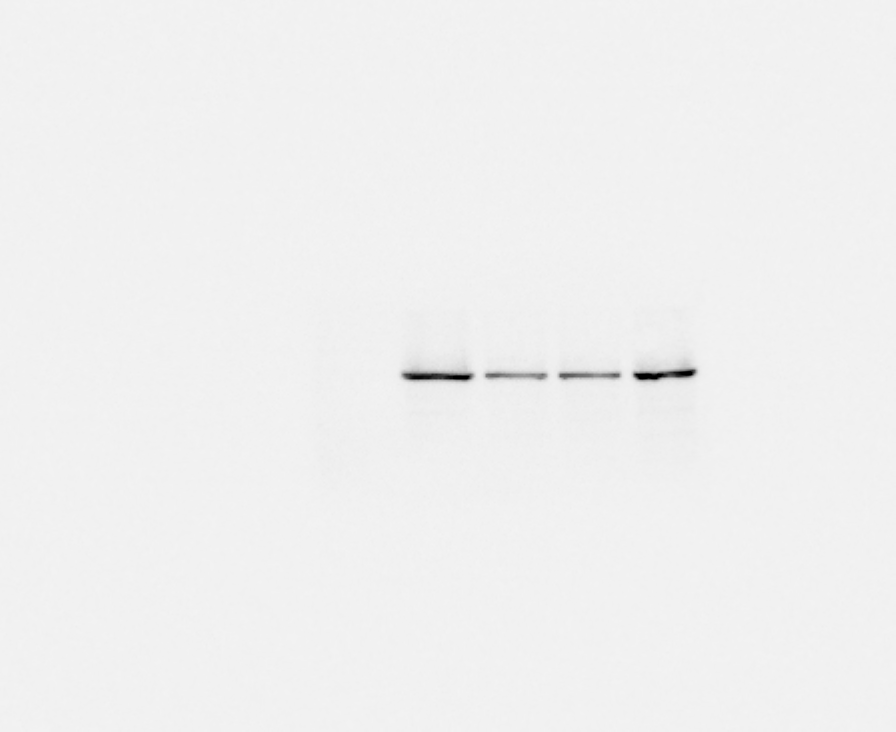


GAPDH


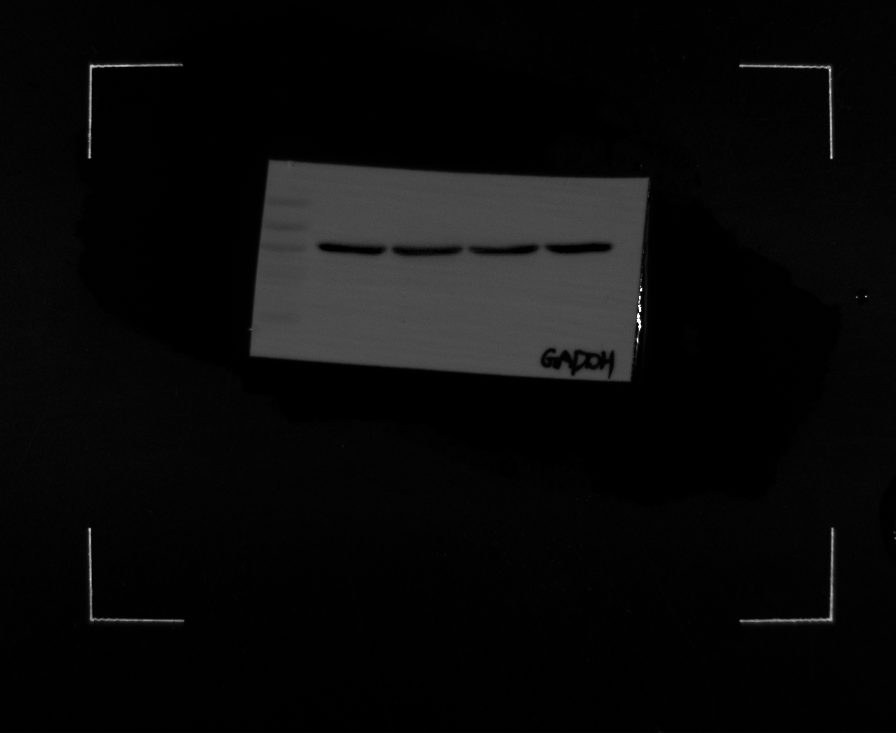

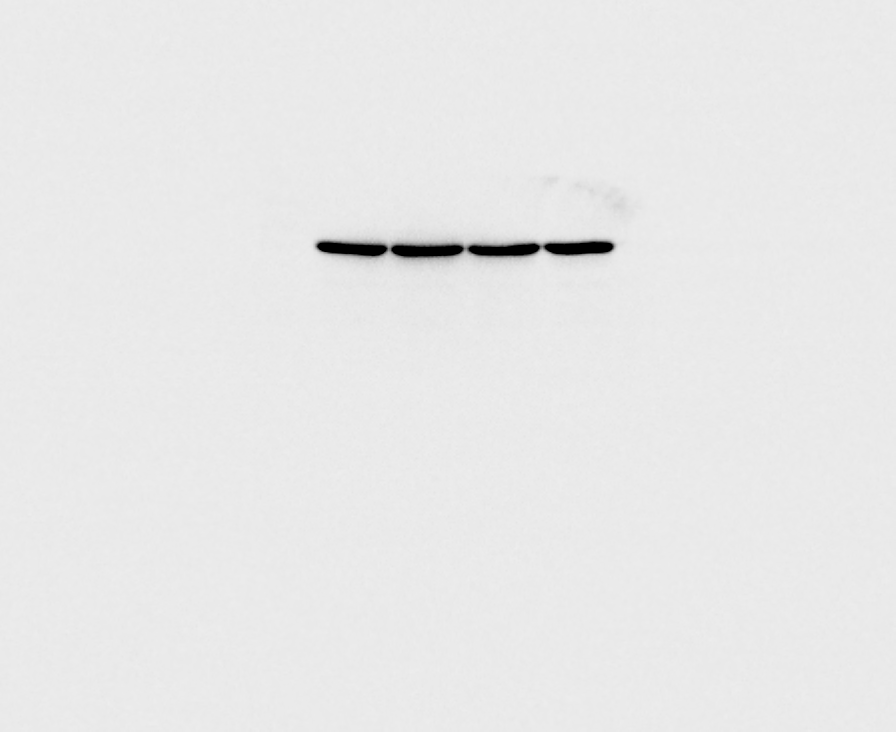


FIG 6A

SENP3


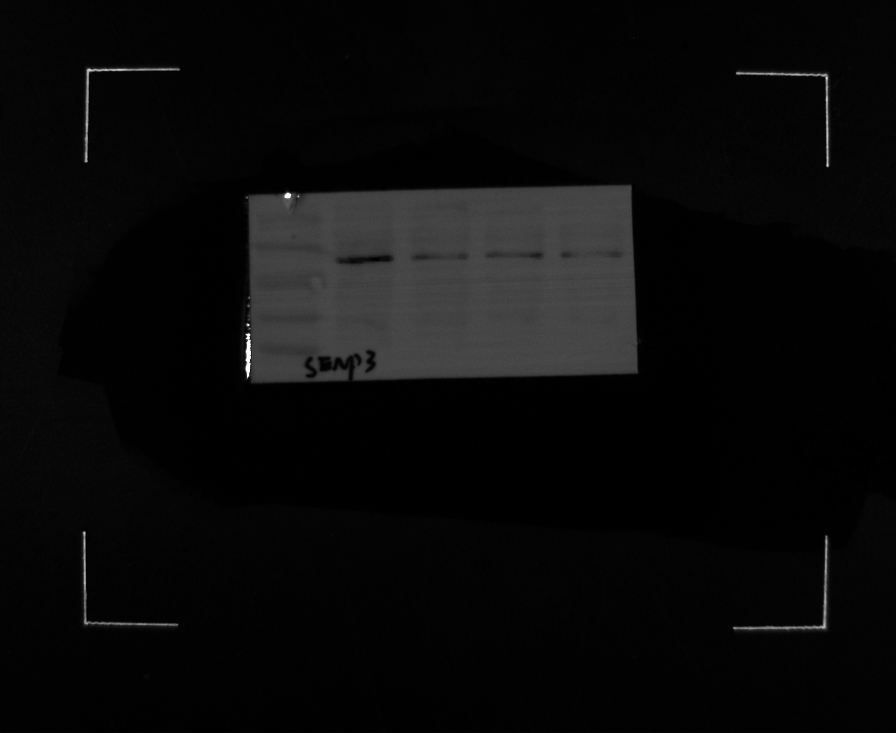

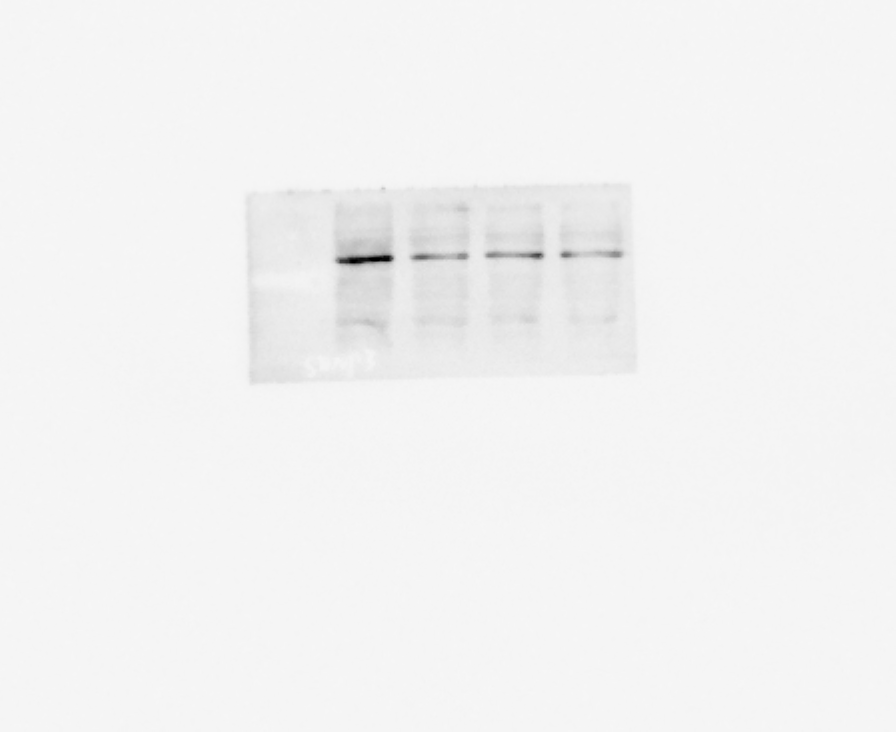


DLX2


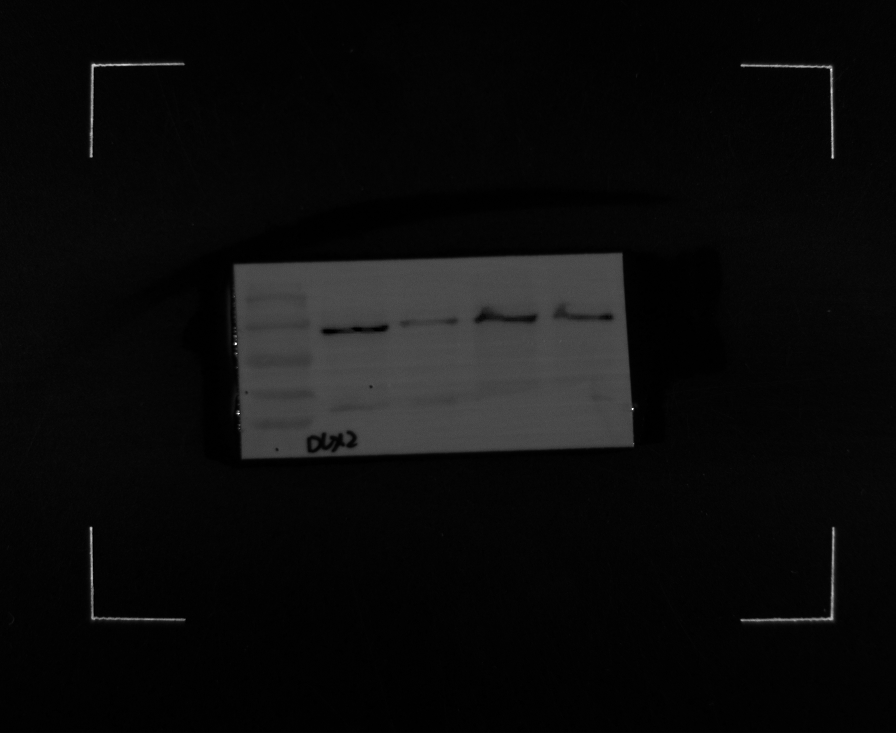

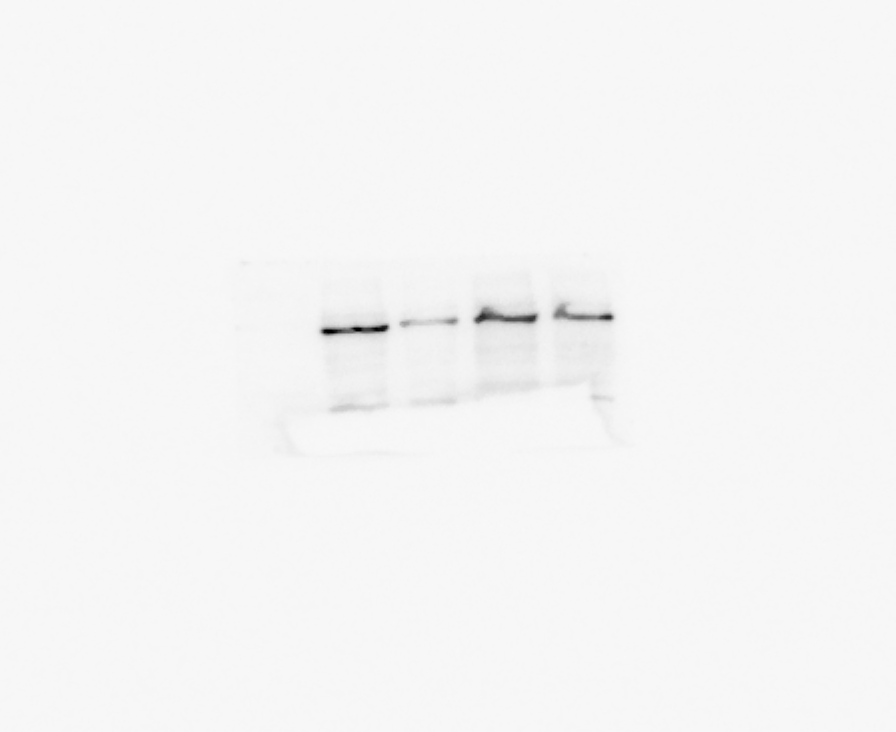


SIRT3


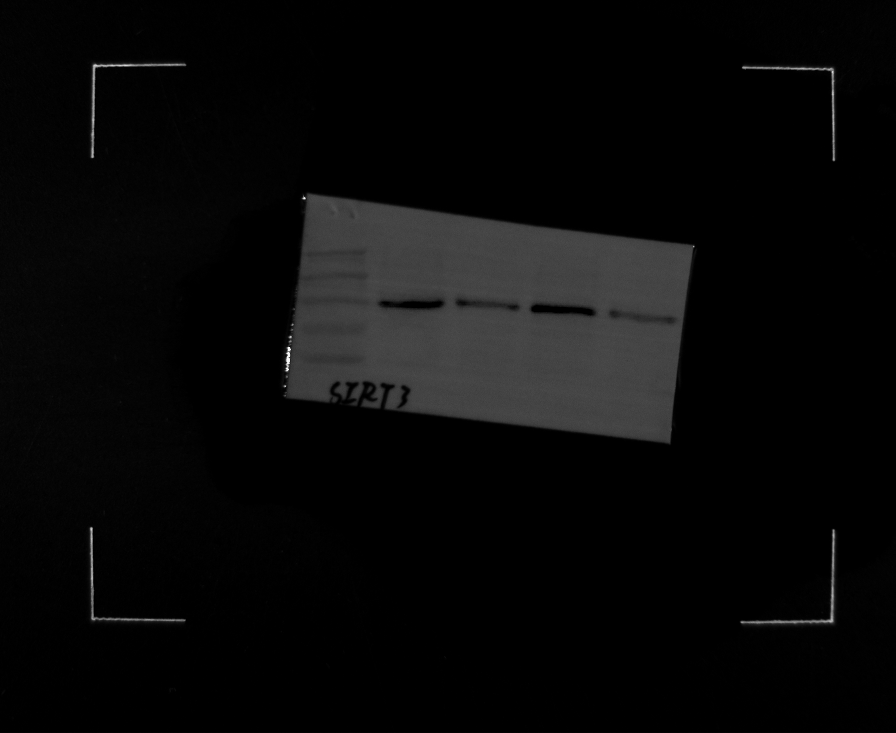

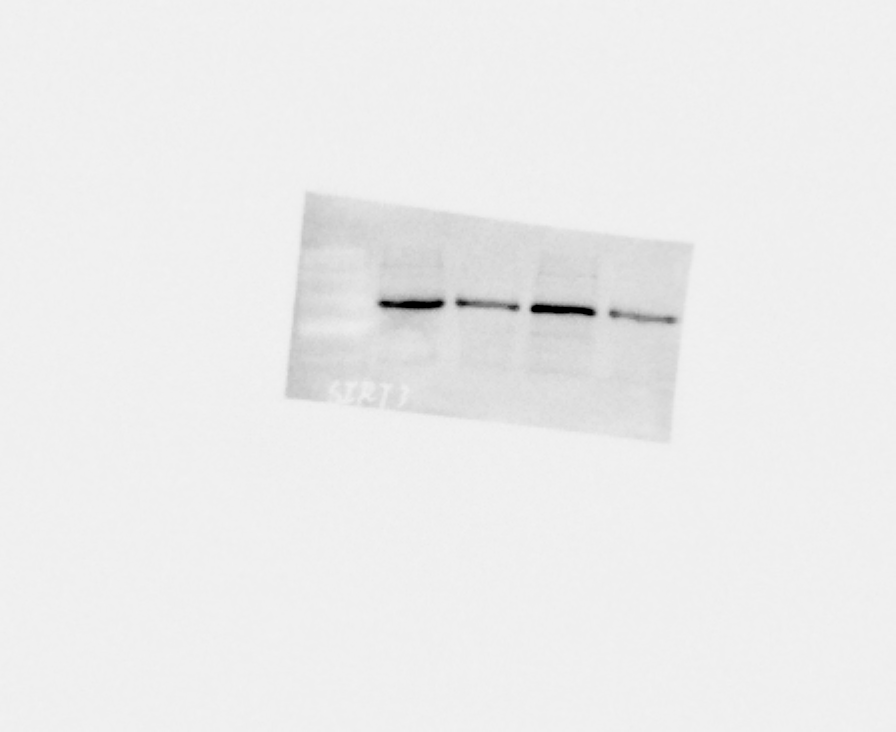


GAPDH


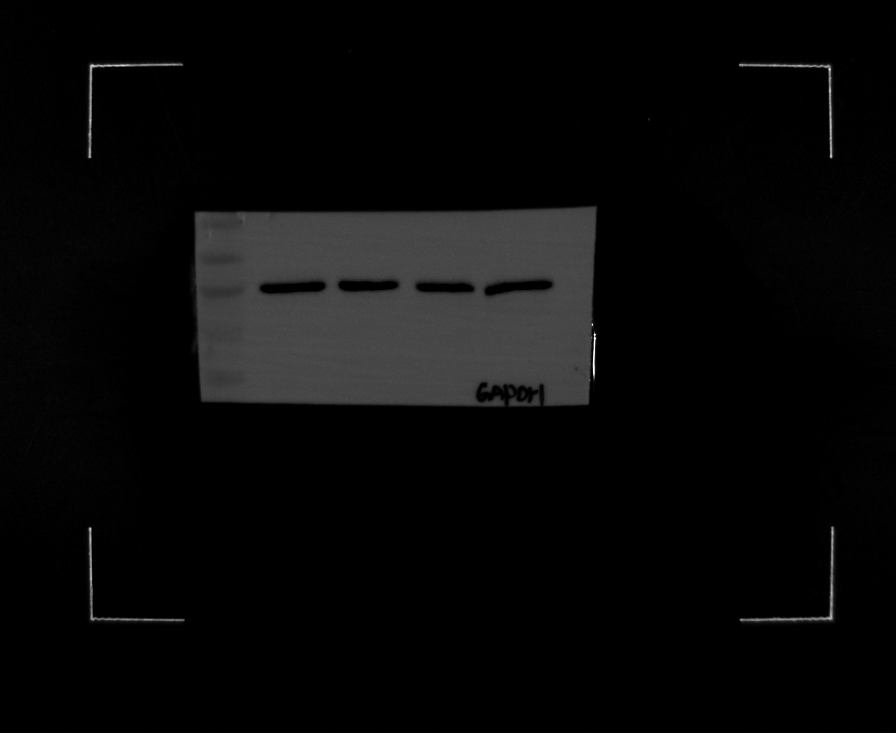

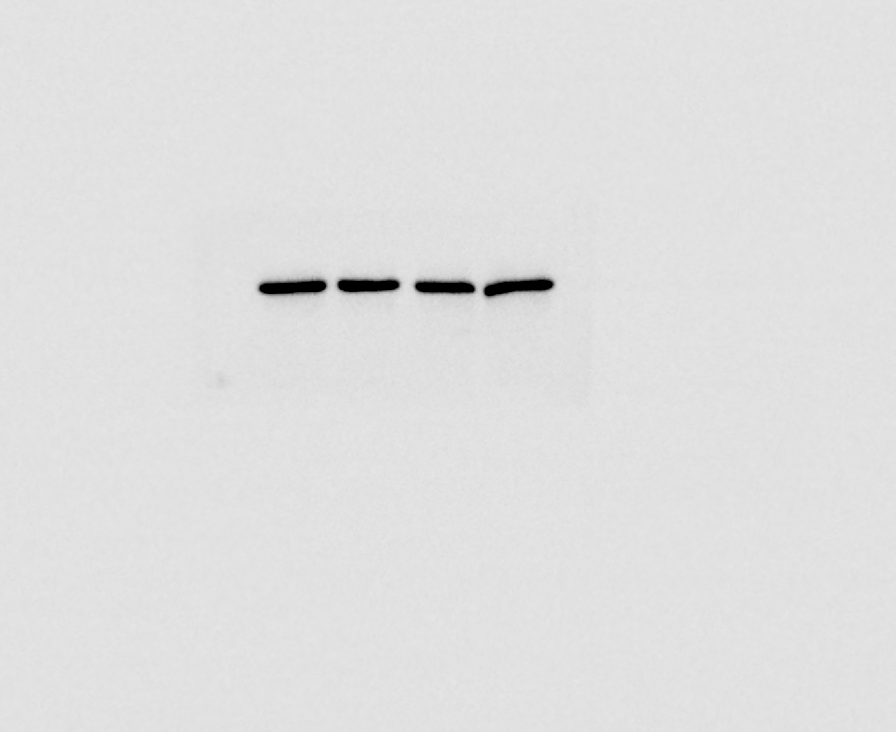


FIG 6F

OPN


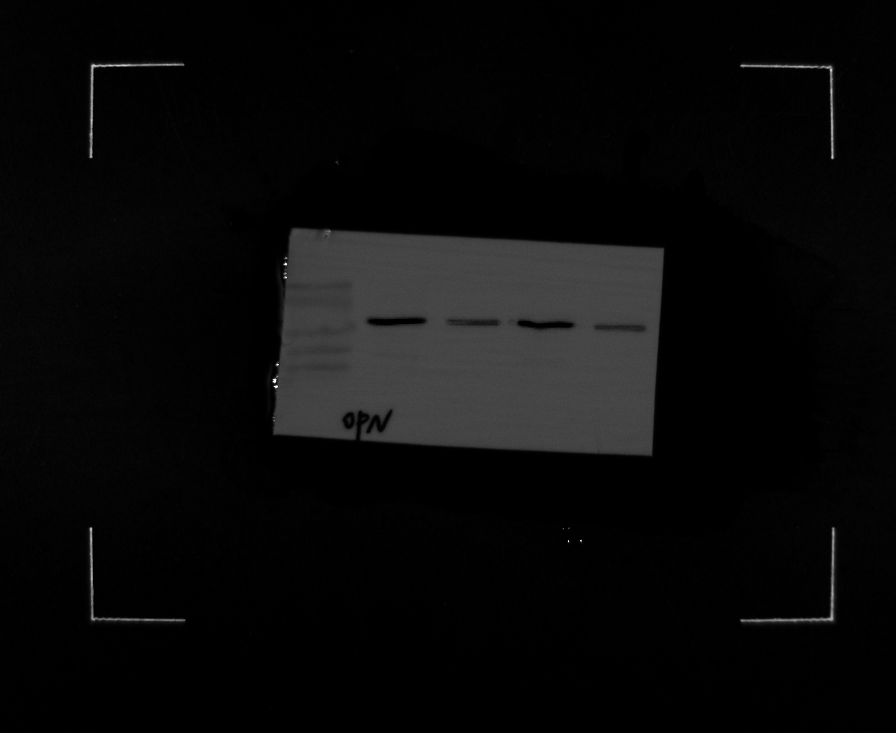

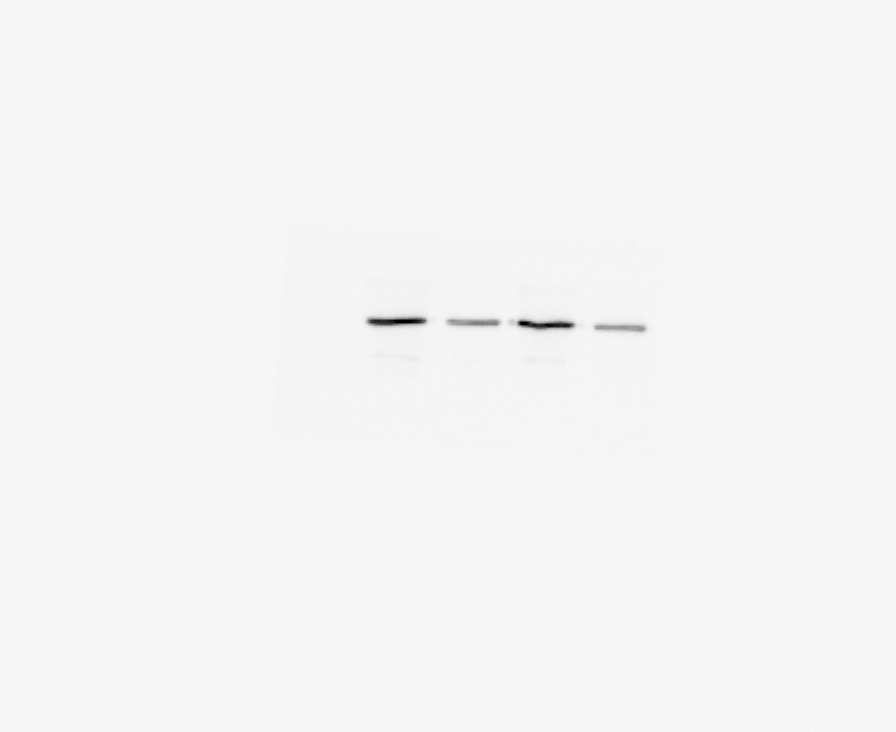


OCN


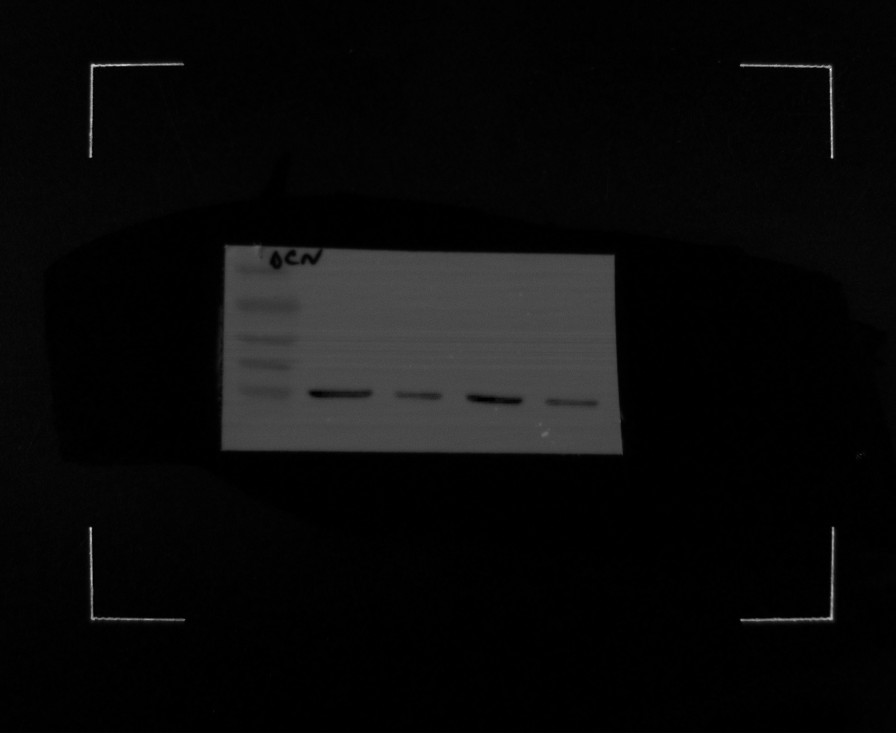

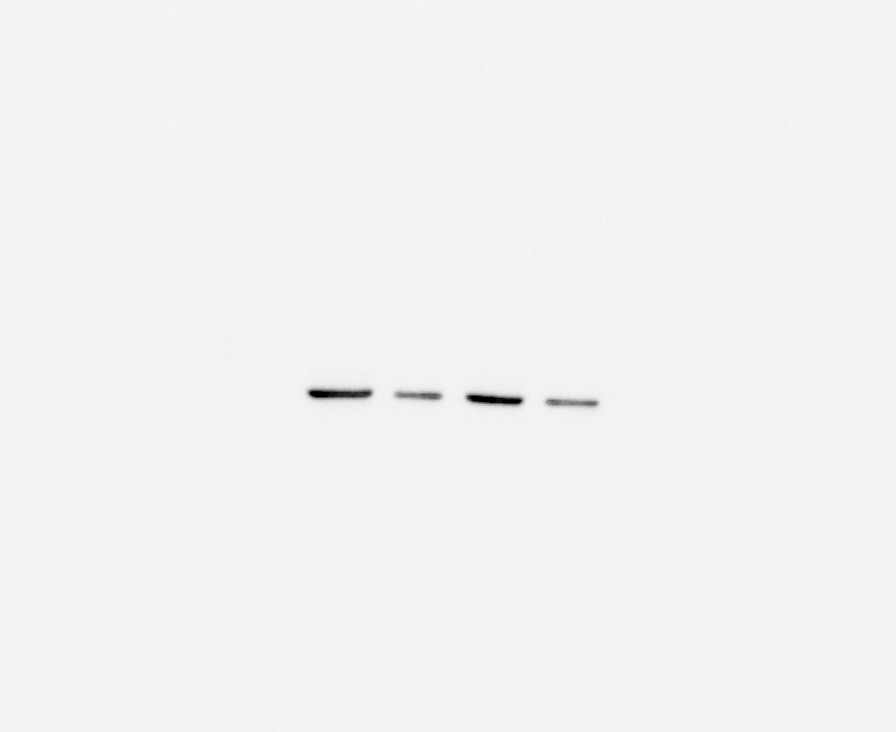


RUNX2


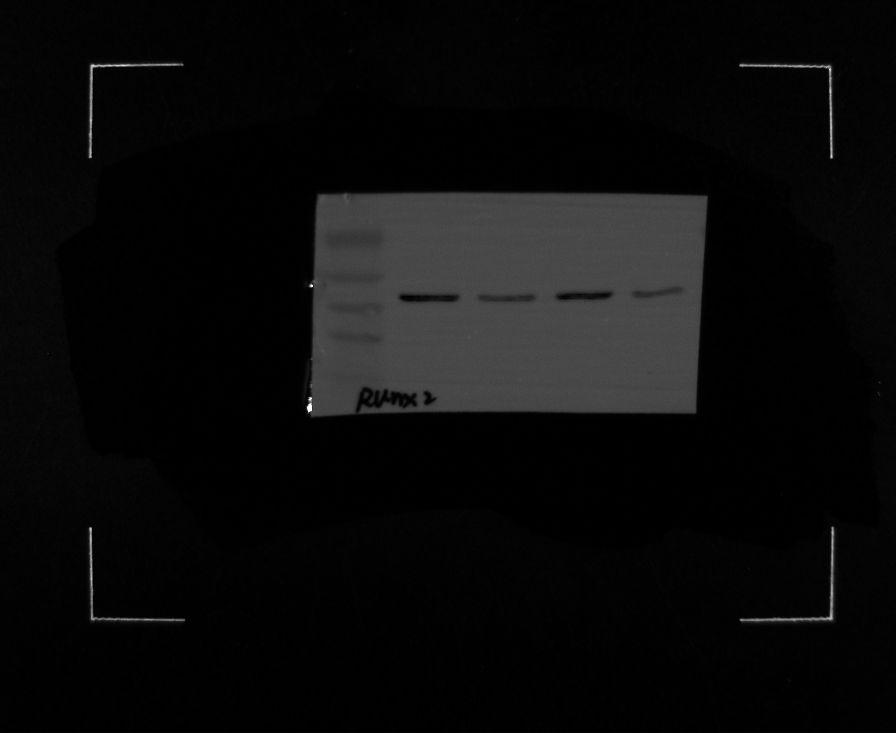

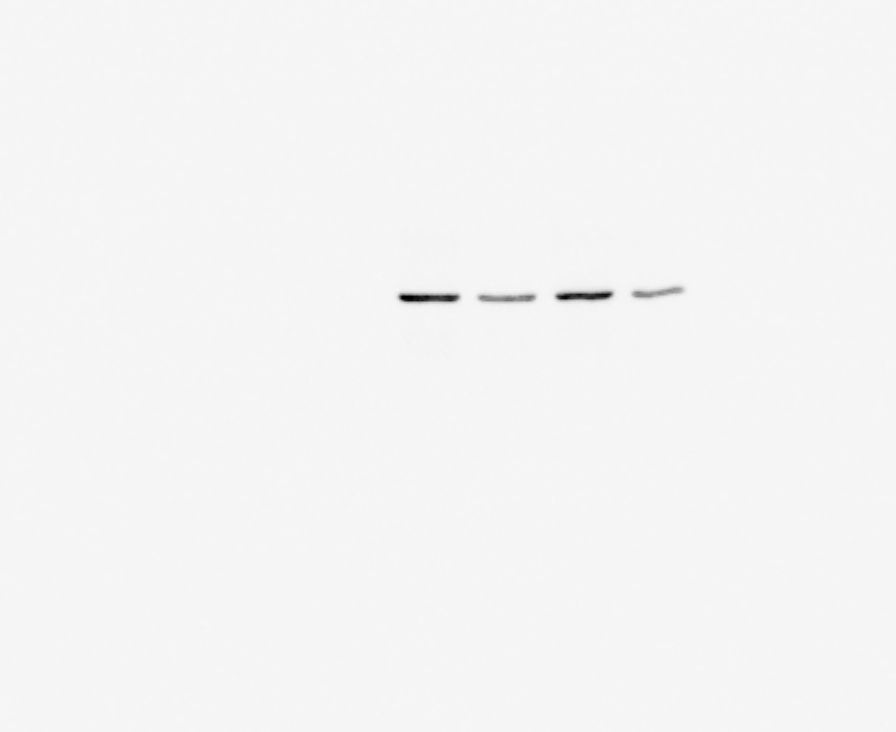


GAPDH


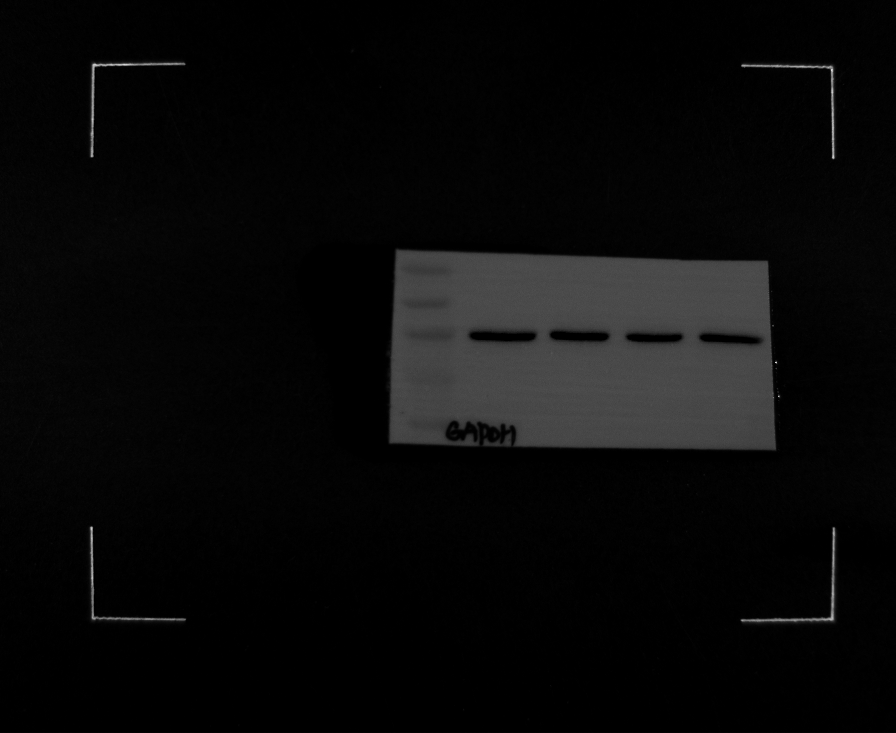

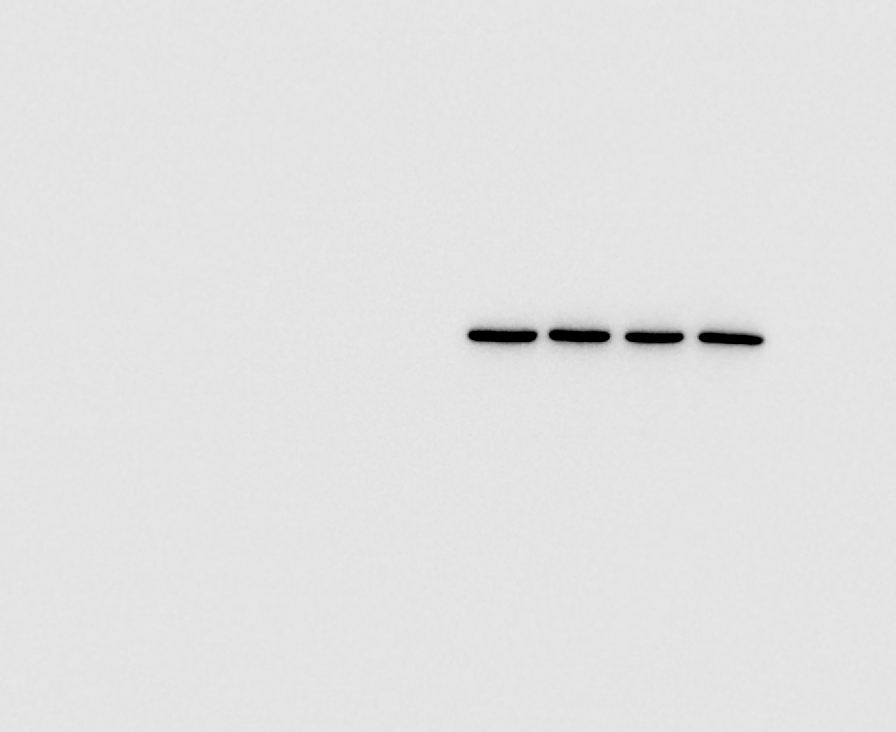


FIG7D

SENP3


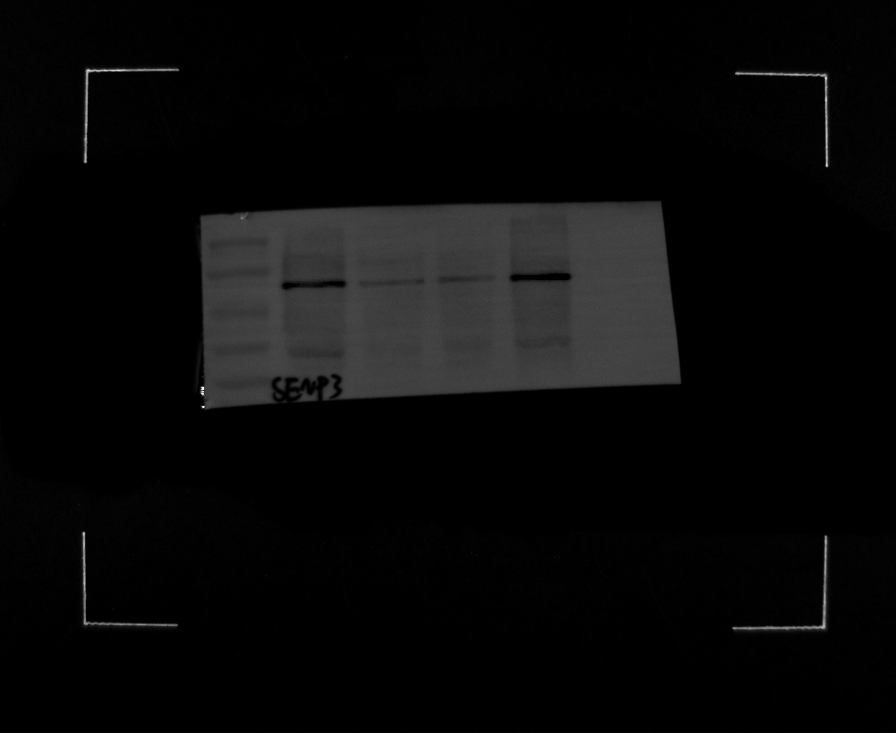

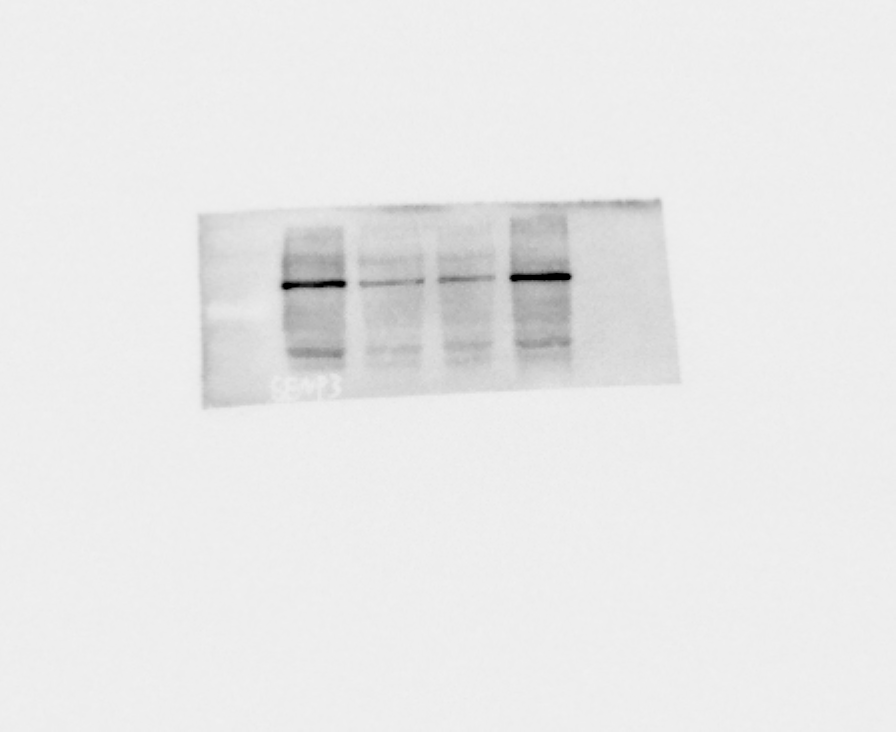


DLX2


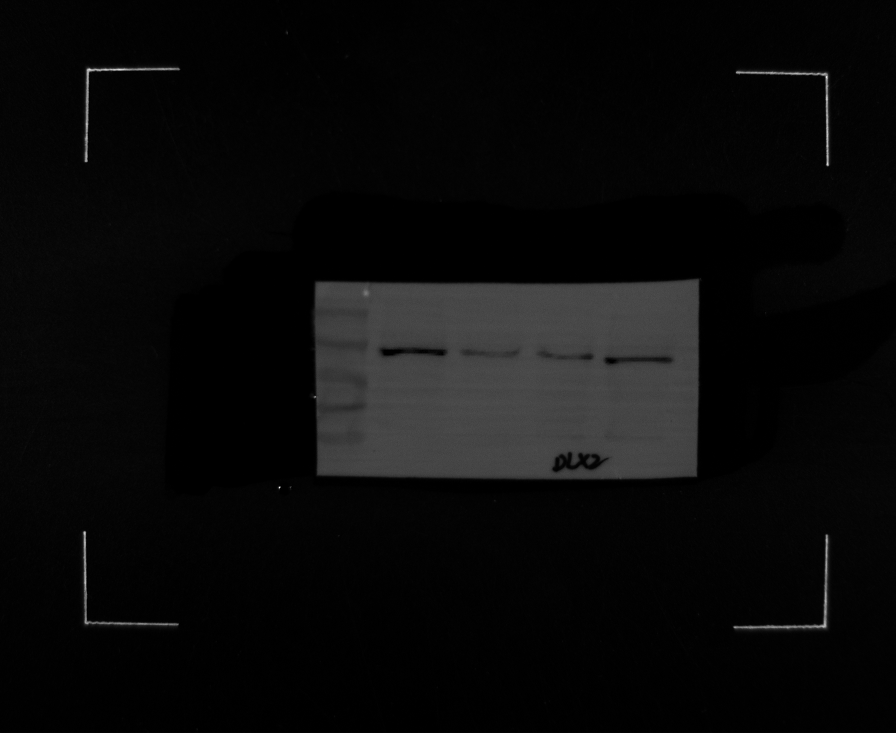

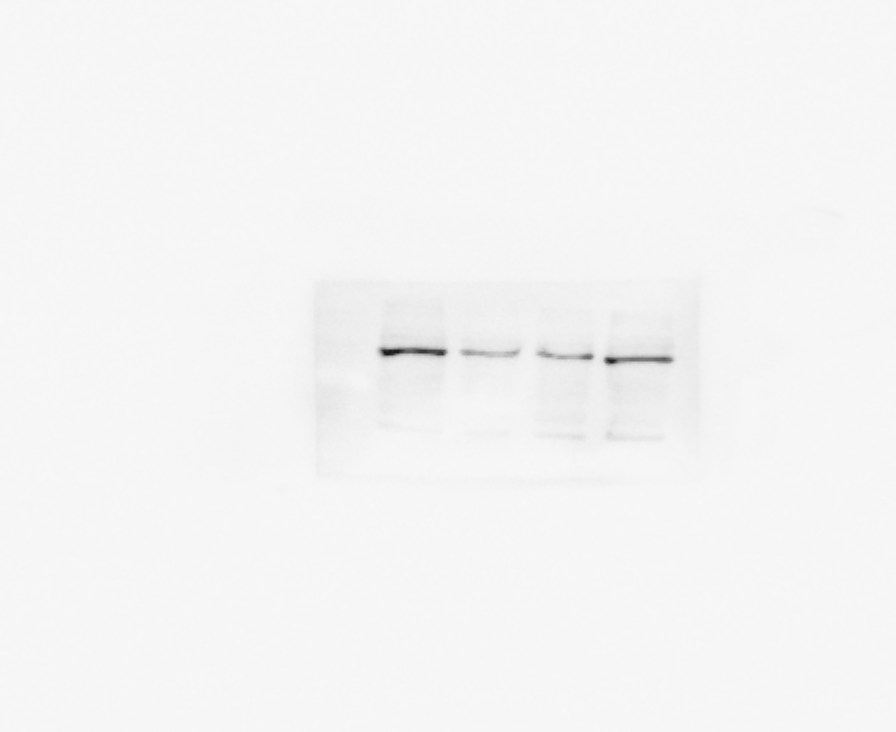


SIRT3


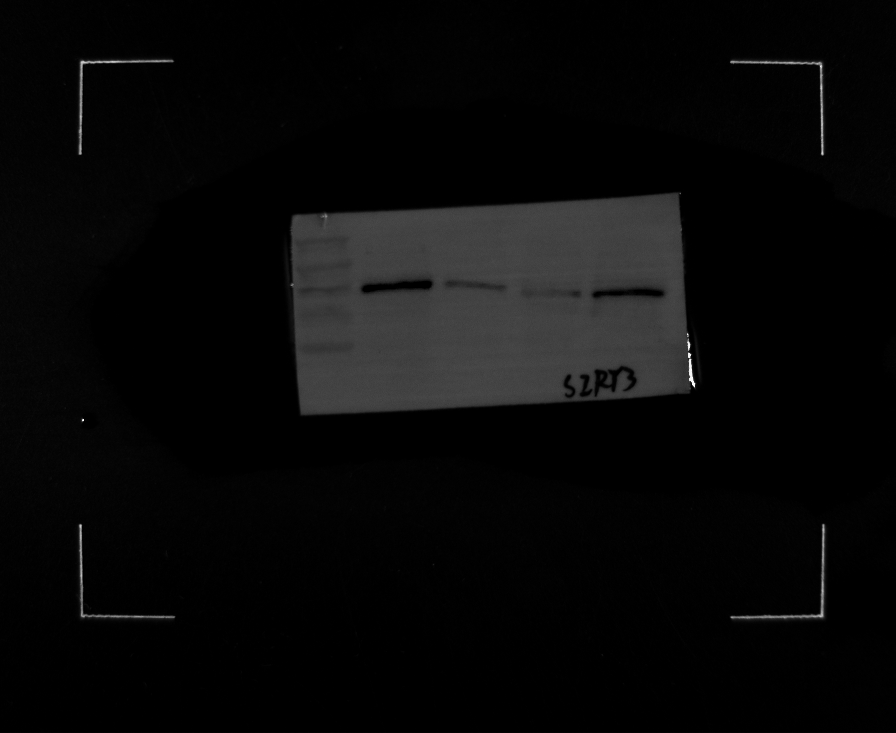

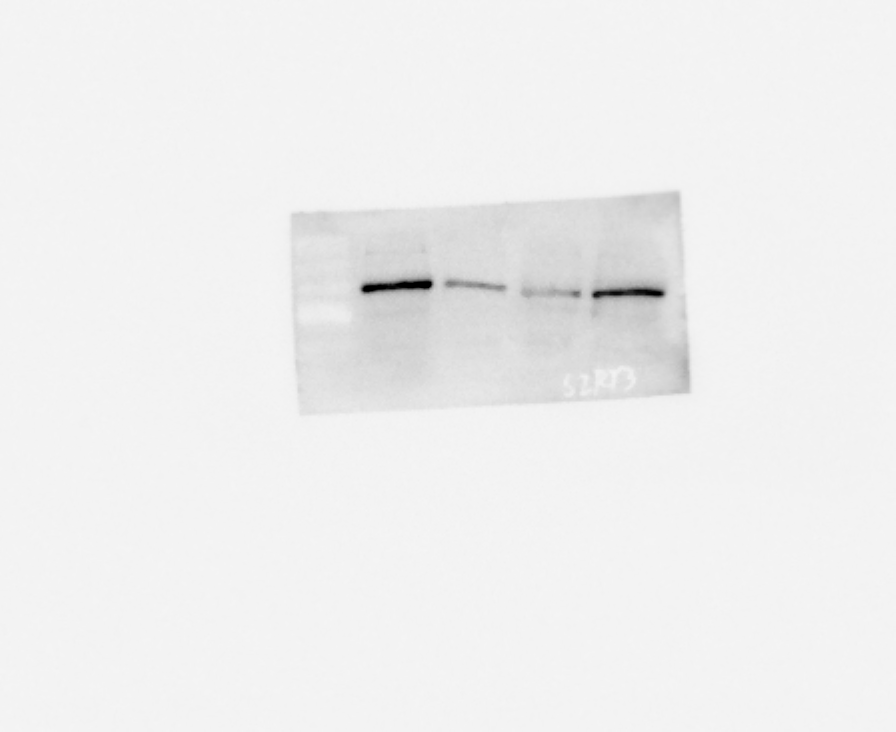


GAPDH


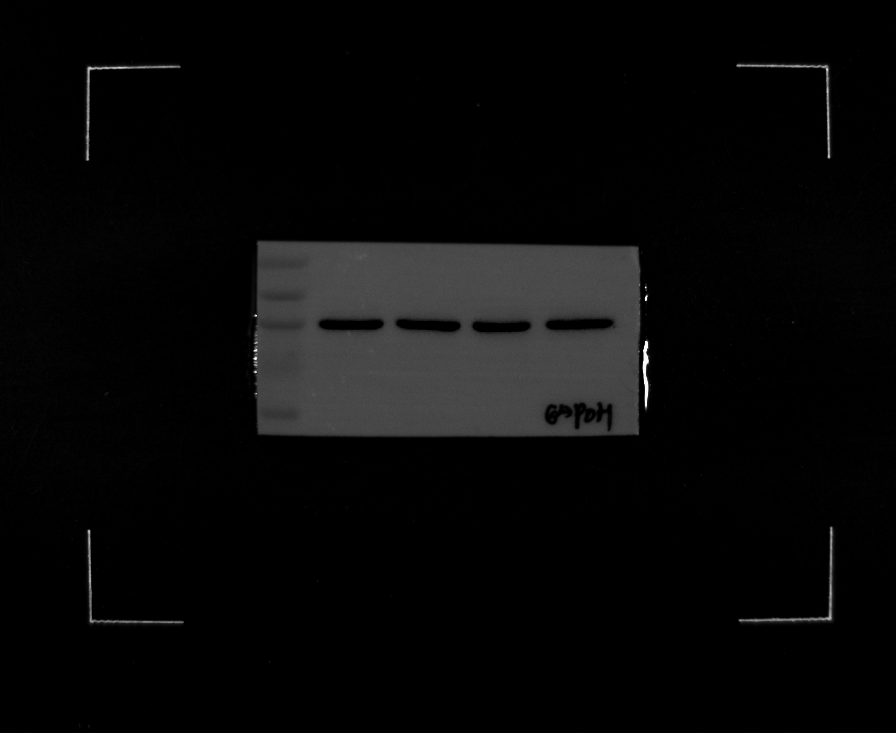

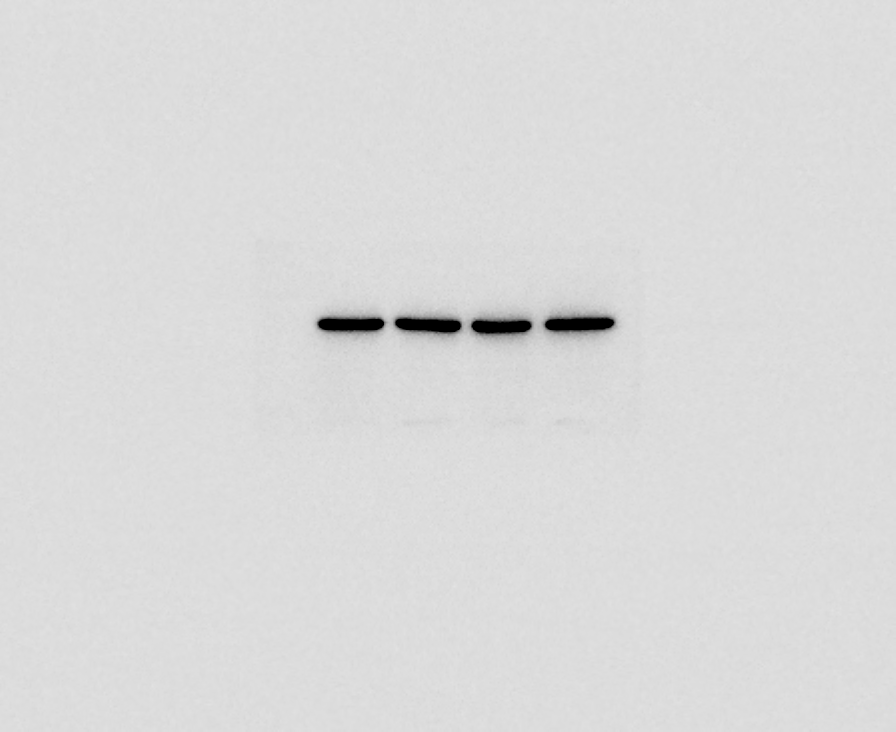


FIG7E

IP:DLX2

DLX2-SUMO2/3-SUMO2/3


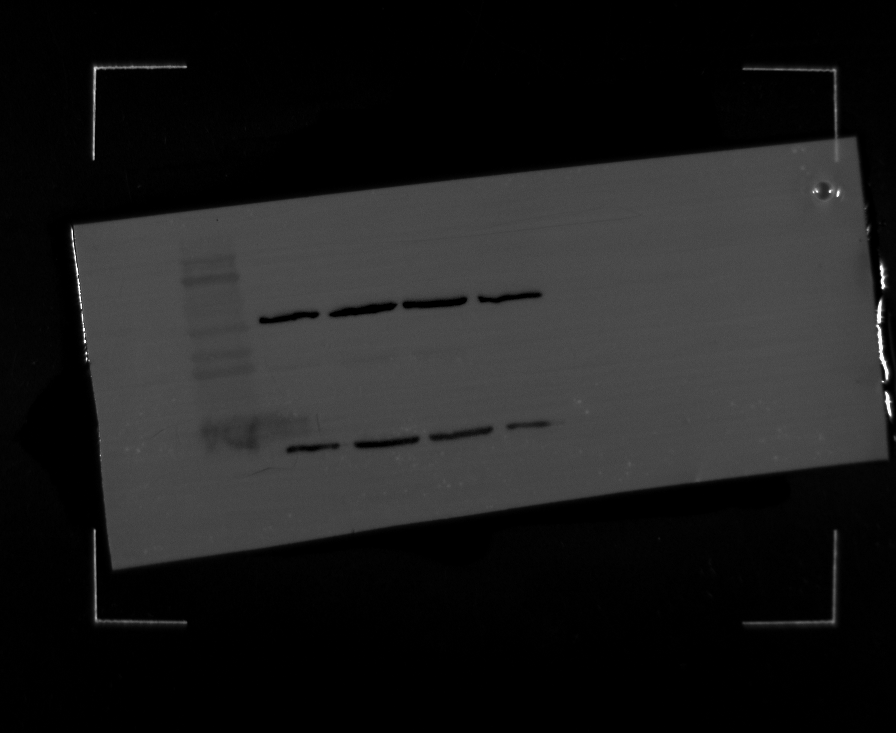

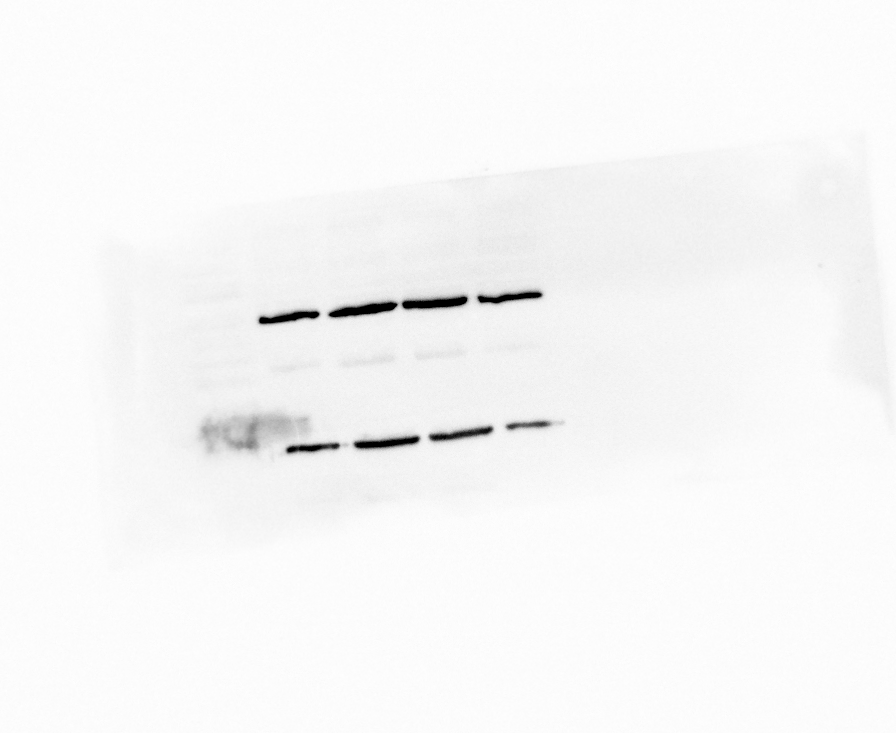


Input

DLX2


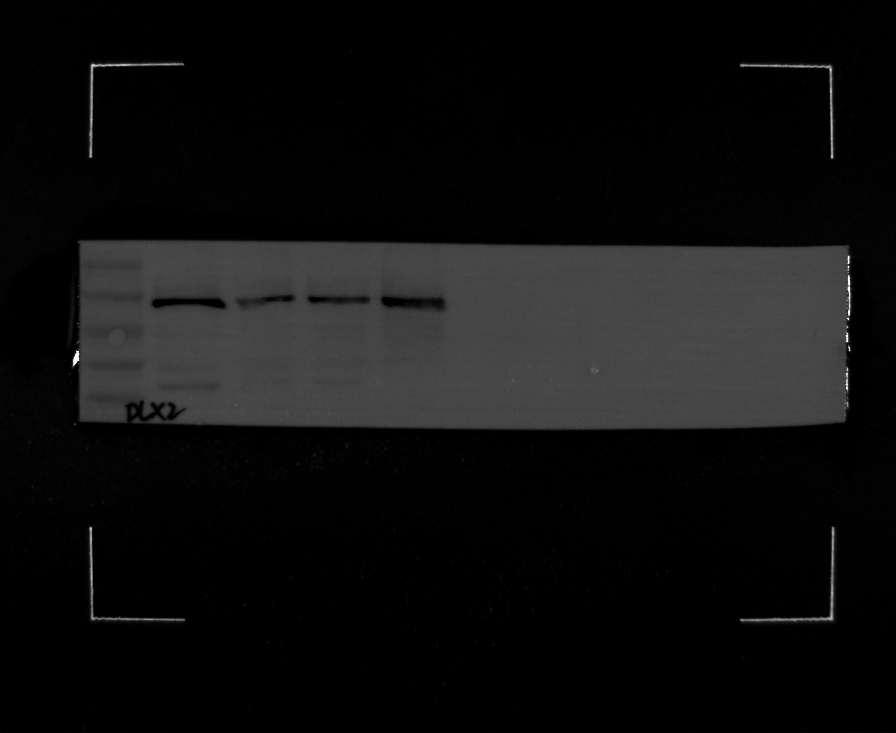

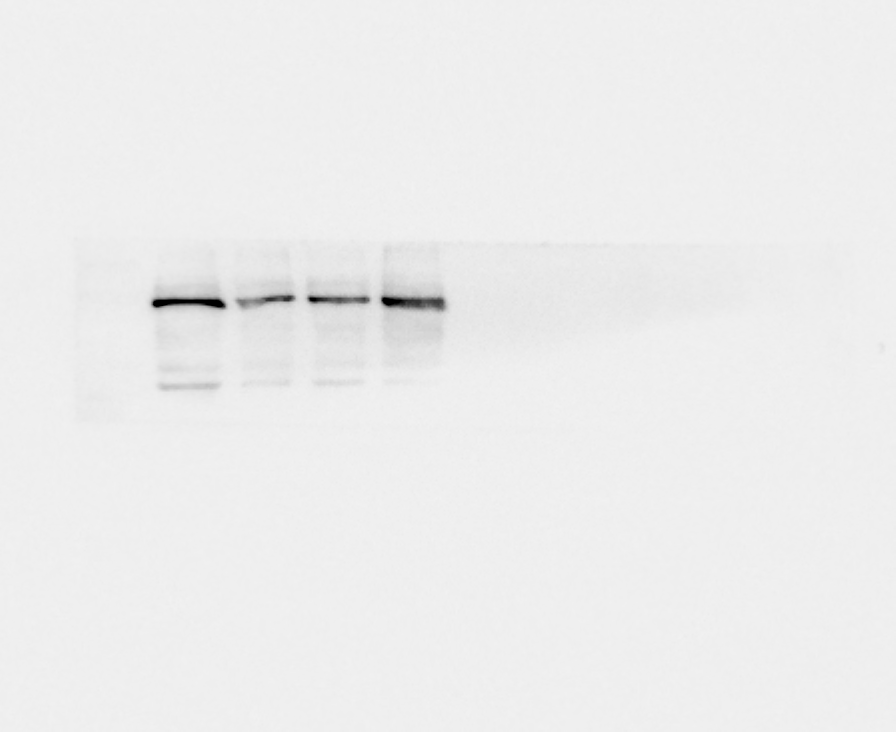


SENP3

GAPDH

FIG 7F

OPN

OCN

RUNX2

GAPDH
